# Phenylene‐Bridged Perylene Monoimides as Acceptors for Organic Solar Cells: A Study on the Structure–Property Relationship

**DOI:** 10.1002/chem.202200276

**Published:** 2022-03-19

**Authors:** Bettina Schweda, Matiss Reinfelds, Jakob Hofinger, Georg Bäumel, Thomas Rath, Petra Kaschnitz, Roland C. Fischer, Michaela Flock, Heinz Amenitsch, Markus Clark Scharber, Gregor Trimmel

**Affiliations:** ^1^ Institute for Chemistry and Technology of Materials (ICTM) NAWI Graz Graz University of Technology Stremayrgasse 9 Graz 8010 Austria; ^2^ Linz Institute of Organic Solar Cells (LIOS) Institute of Physical Chemistry Johannes Kepler University Linz Altenbergerstrasse 69 4040 Linz Austria; ^3^ Institute of Inorganic Chemistry NAWI Graz Graz University of Technology Stremayrgasse 9 Graz 8010 Austria

**Keywords:** density functional calculations, donor-acceptor systems, dyes, organic photovoltaics, perylene monoimide

## Abstract

A series of non‐fullerene acceptors based on perylene monoimides coupled in the peri position through phenylene linkers were synthesized via Suzuki‐coupling reactions. Various substitution patterns were investigated using density functional theory (DFT) calculations in combination with experimental data to elucidate the geometry and their optical and electrochemical properties. Further investigations of the bulk properties with grazing incidence wide angle X‐ray scattering (GIWAXS) gave insight into the stacking behavior of the acceptor thin films. Electrochemical and morphological properties correlate with the photovoltaic performance of devices with the polymeric donor PBDB‐T and a maximum efficiency of 3.17 % was reached. The study gives detailed information about structure–property relationships of perylene‐linker‐perylene compounds.

## Graphical abstract

**Out with the old, in with the new**: Perylene monoimide has long been in the shadow of its famous sibling: perylene diimide. And this, despite its talents! In this study, perylene monoimide takes the spotlight and it has things to tell.

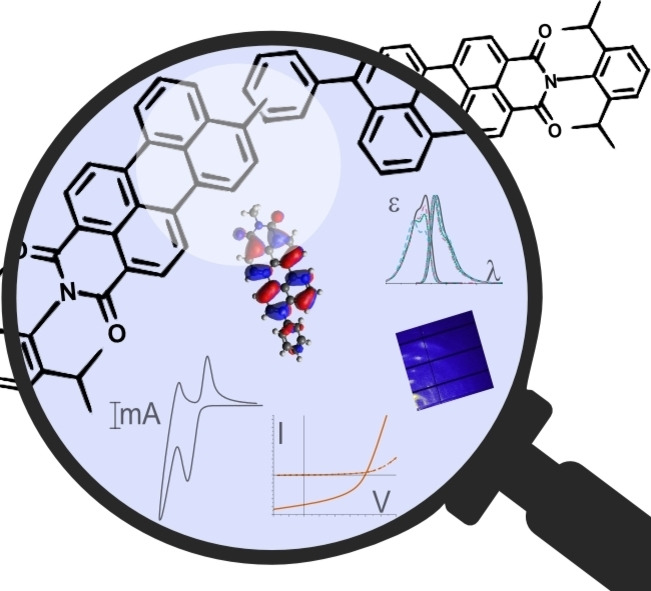

## Introduction

In recent years, small molecule non‐fullerene acceptors (NFAs) outperformed the well‐known fullerenes and their derivatives in organic solar cells.^[1]^ Dyes based on the perylene structure have had an important contribution in this achievement. In fact, perylenetetracarboxylic dibenzimidazole was already used in the first organic heterojunction solar cells by Tang, achieving an efficiency of about 1 % by that time.^[2]^ In later years, perylene diimide derivatives became more widely used. They exhibit high thermal and photochemical stability as well as strong visible light absorption.^[3]^ In order to avoid excessive π‐π stacking, most acceptor structures derive from perylene diimide forming dimers, trimers, tetramers or oligomeric and polymeric materials via linkage through ortho, bay or imide positions.^[4]^ The highest PCE achieved using a perylene diimide as building block is now over 10 %.^[5]^ For a more detailed information the reader is referred to a recent review.^[6]^ The application of perylene diimides does not stop there, as they are used, for example, in single component organic solar cells,^[7]^ organic field effect transistors (OFET),^[8]^ self‐assembly systems^[9]^ and as dyes and pigments.^[10]^

At the same time, perylene monoimide (in this work abbreviated as “P”) has attracted less research attention.^[11]^ Perylene monoimides also have a strong absorption in the visible range, but have higher lying LUMO energy levels (lower reduction potentials) than perylene diimides.^[12]^ This enables reaching of higher open circuit voltages (V_OC_) in solar cells. Due to the asymmetric structure of perylene monoimides, one more position is available for modification, namely, the peri position. This position allows to introduce π‐conjugated substituents along the long axis of the perylene core. This can be advantageous as shown for perylene monoimide‐porphyrine dyads, which had higher rates and efficiencies of energy transfer in comparison to analogue compounds based on perylene diimides.^[13]^ Perylene monoimide has been shown to be a useful building block for the construction of various functional molecules, for example, light harvesting antennas^[14]^ and arrays,^[15]^ as optoelectronic materials in organic,^[16]^ dye sensitized^[17]^ or single material solar cells^[18]^ and solar light concentrators.^[19]^ The coupling of two perylene monoimides (P) through the peri position via π‐conjugated linker molecules (L) leads to an acceptor‐donor‐acceptor (A‐D‐A) like structure P‐L‐P. A large number of high‐performing NFA molecules are based on such an A‐D‐A motif,^[20]^ consequently, studies on such structures based on perylene monoimide have already been conducted. The group of Chen screened various linker molecules based on phenyl and thiophene structures as well as small fused ring systems (based on indacenodithiophene and benzodithiophene building blocks). Despite linker variations, solar cell efficiencies did not exceed 1 % (using PCE‐10 as donor polymer).^[21]^ Cremer and Bäuerle synthesized P‐L‐P structures with oligo‐thiophene based linkers. The absorption spectra of these compounds could be broadened without influencing the HOMO/LUMO energy levels much upon increased oligomer length. Solar cell devices reached a maximum of 0.2 %, albeit here the fullerene derivative PCBM was used as acceptor.^[22]^ The group of Ma introduced alkynyl linkers and also reached roughly 1 % in solar cell devices (PBDB‐T as donor).^[23]^ Using alkylated fluorene as linker and P3HT as donor polymer, a PCE above 2 % was reported by the group of Cheng,^[24]^ while the group of Li reported efficiency values of up to 6.0 % and an impressive V_OC_ of 1.3 V after replacing the donor polymer with PTZ1.^[25]^

In our own recent work, three different linker molecules based on fluorene, carbazole and silafluorene were investigated. With all three acceptors, efficiencies over 5 % and V_OC_s over 1 V could be reached (PBDB‐T as donor polymer).^[26]^ A π‐extended linker based on tetraoctyl‐indeno[1,2‐b]fluorene in combination with the D18 polymer allowed to boost the V_OC_ to 1.4 V (with a PCE over 5 %).^[27]^ These promising results motivated us to perform a detailed structure–property relationship study on these P‐L‐P systems. To that end, we have chosen the simplest aromatic linker – benzene. This gives the possibility to investigate the influence of the various attachment positions (para vs. meta vs. ortho). Furthermore, the benzene ring can be easily modified in order to further tune the properties of the molecule, for example, the solubility. We could prepare the perylene monoimide building block in multigram scale and P‐L‐P structures were then accessed via Suzuki‐coupling. With the target materials in hand, a thorough structure–property analysis was performed. For this purpose, optical spectroscopy (in solution and thin films) and cyclovoltammetry was combined with density functional theory (DFT) computations. Furthermore, the bulk material properties were elucidated using grazing‐incidence wide‐angle X‐ray scattering (GIWAXS) and thermogravimetric analysis (TGA). Finally, the materials were tested in organic solar cells with the polymeric donor PBDB‐T and the device performance was analyzed in the light of P‐L‐P compound structural properties.

## Results and Discussion

### Synthesis

**Scheme 1:**
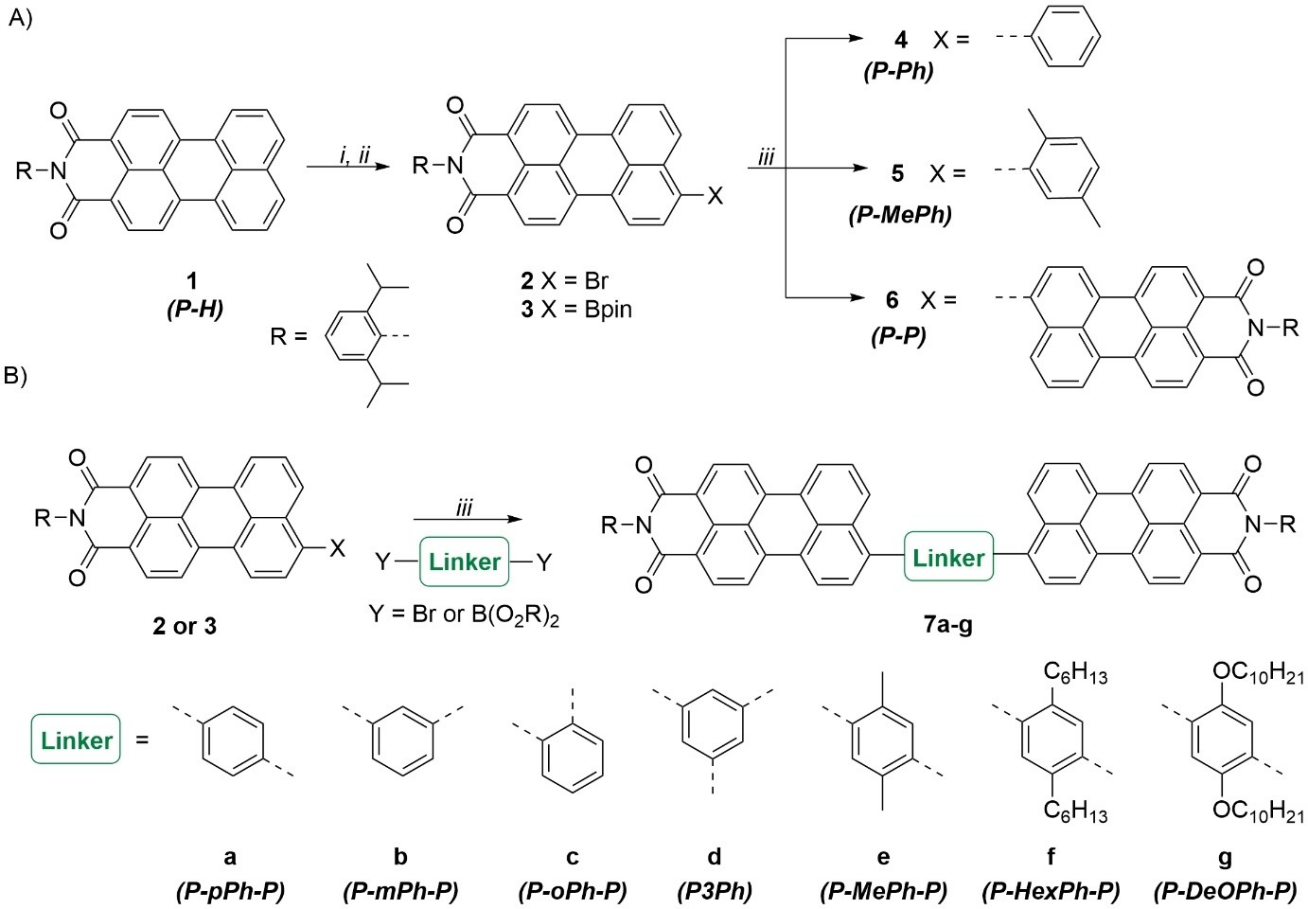
Synthesis overview. A) Synthesis route of P−X based molecules. B) Synthesis route of P‐L‐P based acceptors; *i*: Br_2_ in AcOH at r.t., *ii*: B_2_Pin_2_, Pd(dppf)Cl_2_, KOAc in 1,4‐dioxane at 80 °C, *iii*: Suzuki coupling catalyzed by Pd(PPh_3_)_4_, using 1 M K_2_CO_3_ or KF, some cases with Aliquat 336, in toluene or EtOH/THF at 80–110 °C (for more detailed information, see the compound of interest in the Supporting Information).

Scheme 1 depicts the overall synthesis scheme. Suzuki reaction was chosen as the key step in the preparation of the P‐L‐P type structures. First, the perylene **1** was prepared from the commercially available perylene tetracarboxylic acid anhydride in moderate yield following a literature known procedure (for more information see the Supporting Information).^[28]^ Bromination to compound **2** proved to proceed cleanest when done at room temperature in acetic acid using an excess of bromine.^[29]^ From the brominated product **2** the boronic acid pinacol ester derivative (**3**) was prepared via a palladium catalyzed borylation. Neither of these three synthesis steps required column chromatography for purification. Thus, these compounds could be easily prepared in a multigram scale. Bromide **2** and pinacol ester **3** were coupled with simple linker molecules (dibromides or diboronic acid derivatives) to give the P‐L‐P structures **7 a**–**g**. Generally, the Suzuki coupling reaction proceeded with mediocre yields. The coupling proceeded much better if only one C−C bond had to be produced (compounds **4**–**6**). When K_2_CO_3_ was used as a base, in combination with boronic acid linkers, protodeboronation of monolinked molecules was observed. While, if compound **3** was used as a starting material (and KF instead of K_2_CO_3_), homocoupling to compound **6** was observed. This turned out to be particularly problematic for compound **7 e**, as it has an identical retention factor with molecule **6**. This and other unidentified side products together with very bad solubility of some final compounds (e. g. **7 a**) in all common solvents make purification particularly challenging. A potential way to improve the yields of the Suzuki coupling could be the change of the catalysts.^[30]^ However, we observed that improvements can also be made if the perylene component and the linker are initially mixed in a lower stoichiometric ratio than necessary for a full conversion. Then the perylene component is added dropwise to the reaction mixture until a full conversion is obtained. This turned out to be particularly useful if the pinacol ester **3** was used in order to minimize the homocoupling to product **6**.

### Computations

In order to gain insight into the molecular geometry, electron distribution and excited state properties we performed density functional theory (DFT) computations using the Gaussian 09 program package.^[31]^ After a small benchmarking study on model compound **1** – P‐H (for more information, see DFT computations chapter in the Supporting Information) B3LYP functional and 6‐31G(d,p) basis set were chosen for geometry optimization. The minima were verified by frequencies calculation. Natural atomic orbital analysis was done according to literature.^[32]^ TD‐DFT computations were done using the basis set 6‐31+(d,p) with diffuse functions, and the first 10 excited states were always computed. For calculations empirical dispersion correction (GD3) was used.^[33]^

### Perylene monoimides (A–D structures)

**Figure 1:**
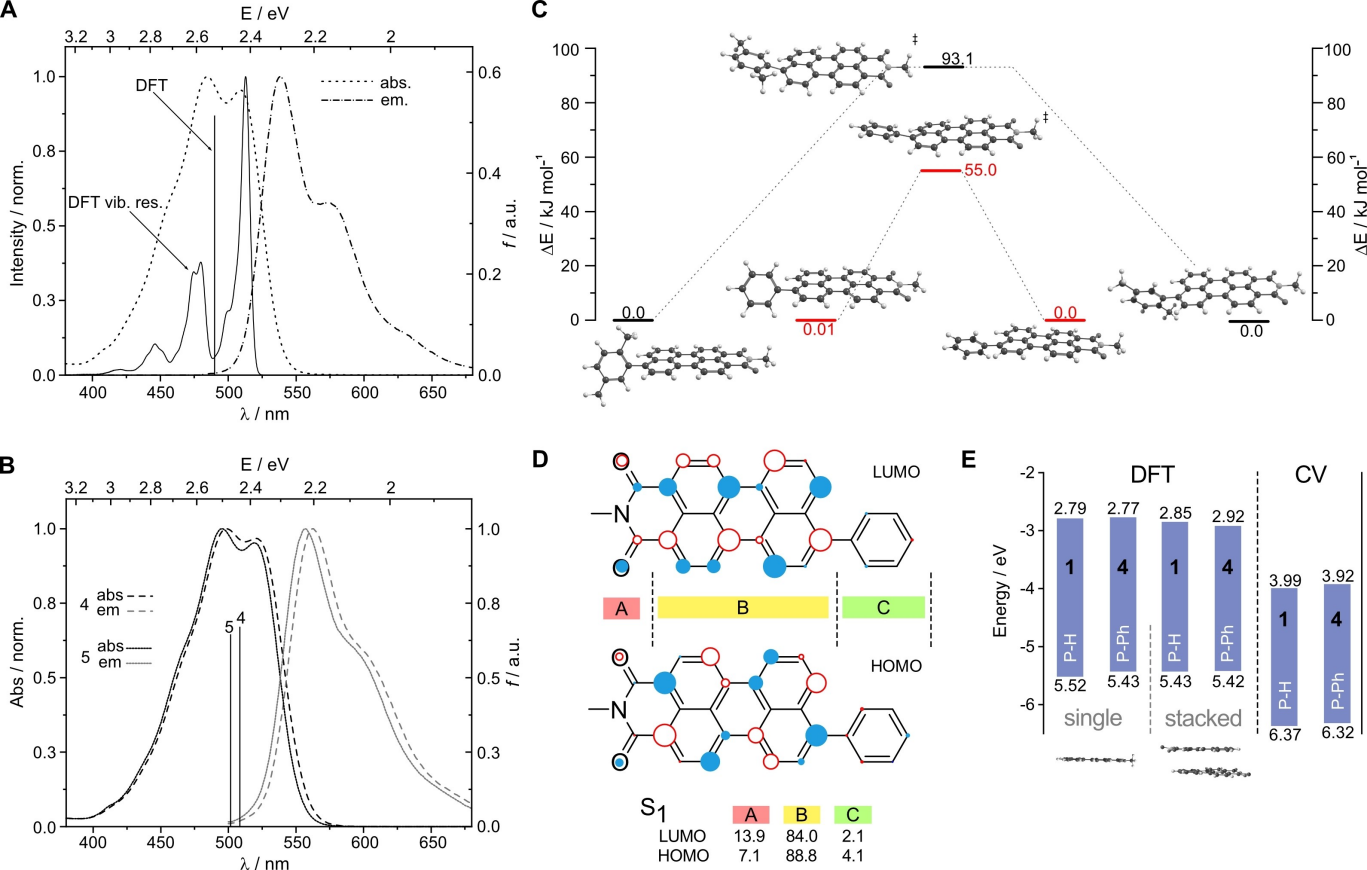
A) Absorption and emission spectra of perylene monoimide 1 (in CHCl_3_). Calculated excitation energy and oscillator strength shown by solid bar (DFT). Calculated, vibrationally resolved spectrum shown by solid line (DFT vib. res.). B) Absorption and emission spectra of perylene monoimides 4 and 5 (in CHCl_3_). Calculated excitation energy and oscillator strength shown by solid bar. C) Calculated changes in energy upon rotation of the phenyl substituent in compounds 4 and 5. D) LUMO and HOMO of perylene monoimide 4 (circle size indicates the electron density, filled and empty circles have opposite phases). The percentage of electronic density on various molecular fragments are given for the S_1_ state. E) Calculated and measured (ox/red potential vs. Fc/Fc^+^ with Fc/Fc^+^ vs. vacuum=−5.39 eV) energy levels for perylene monoimides 1 and 4. Calculations were done for a single molecule (single) and for two stacked molecules (stacked).

First, we investigated perylene monoimide **1** (P‐H) and two derivatives with P‐L structure. Here, as L unit phenyl **4** (P‐Ph) and 2,5‐dimethylphenyl **5** (P‐MePh) groups were chosen. These compounds serve as basis for the description of the larger P‐L‐P systems. In Figure 1A the absorption and emission spectra of the unsubstituted P‐H (**1**) in chloroform solution are shown. In the absorption as well as in the emission spectrum, two maxima can be recognized (at 484 and 509 nm, as well as at 538 nm and 575 nm, respectively). TD‐DFT computations of the first 10 excited states found that only the S_1_ is a bright state with an excitation energy of 2.53 eV (491 nm with oscillator strength *f*=0.51, see the solid bar in Figure 1A). The predicted excitation energy for the S_10_ state is 4.12 eV (301 nm, *f*=0.003), thus the multiple maxima in the absorption spectrum must arise from vibrational contributions as known for perylenes.^[34]^ To probe this, we computed vibrationally resolved electronic spectra.^[35]^ The results are in a very good agreement to the experimental spectrum, confirming that the observed spectral features are from vibrational contributions (solid line Figure 1A, DFT vib. res.). Also, for phenyl substituted perylene monoimides P‐Ph (**4**) and P‐MePh (**5**) one bright state was found by TD‐DFT computations, in both cases it was the S_1_ state which corresponds to a HOMO‐LUMO transition. For P‐MePh (**5**) which contains a methyl substituent in the *ortho* position of the phenyl ring, a slightly higher excitation energy than for the P‐Ph (**4**) is predicted.

In the experimental results (Figure 1B and Table 1) the same observations can be made. Nevertheless, both compounds have a red‐shifted longest wavelength absorption band maximum (519 nm and 521 nm for P‐MePh and P‐Ph, respectively) when compared to the unsubstituted P‐H (509 nm). The slightly higher excitation energy of compound **5** is due to a larger dihedral angle between the perylene core and the phenyl substituent resulting from the steric hindrance of the methyl group in the ortho position. The energetic minimum for compound **4** was found at a dihedral angle of 56°, while for compound **5** the optimal angle is slightly larger at 72°. Also, in a single crystal of compound **4** the crystal data show a similar dihedral angle of 63° (see Figure S11). In the crystal structure, two rotational isomers, resulting from the C−C bond between the perylene core and the phenyl substituent, can be recognized in an equal ratio. Both isomers have identical calculated energies, and the rotation barrier is estimated to be 55 kJ mol^−1^ (Figure 1C). The introduction of a methyl group on the phenyl linker increases the rotation barrier to 93 kJ mol^−1^. This value is in excellent agreement with the experimentally obtained value for a naphthalene with an *o*‐methylphenyl substituent (93 kJ mol^−1^).^[36]^ Thus, for compound **5** (and also compound **4**) rotation around the C−C bond is expected to be possible at room temperature. This could be confirmed by NOESY1D spectra in which a cross relaxation between the methyl protons and the aromatic protons of the linker, both in ortho position, with three perylene core protons could be seen for compound **5** (Figure S32).

**Table 1:** Experimental and computed (B3LYP‐GD3/6‐31+G(d,p)) optical data of all compounds.

Compound	Computed			Absorption in CHCl_3_		Emission in CHCl_3_				$E_{gap}^{opt}$ ^[a]^ in CHCl_3_	Abs film	Em film	Stokes shift	$E_{gap}^{opt}$ ^[b]^ in film
	state	[eV]/[nm]	f	$\lambda_{max}$ [nm]	ϵ [M^−1^ cm^−1^]	$\lambda_{max}\;$ [nm]	Δ [nm]/[eV]	$\Phi_{fl}$	$\tau_{fl}\;$ [ns]	eV	$\lambda_{max}$	$\lambda_{max}$	Δ [nm]/[eV]	[eV]
**1** (P‐H)	S_1_	2.53/491	0.51	484	39100	538	28/0.13	0.76	4.9	2.36	495	645	150/0.58	2.15
**4** (P‐Ph)	S_1_	2.44/508	0.67	499	35000	562	38/0.16	0.75	4.4	2.28	500	650	150/0.57	2.13
**5** (P‐MePh)	S_1_	2.47/502	0.65	495	39600	557	36/0.15	0.75	4.6	2.30	495	640	145/0.57	2.19
**6** (P‐P)	S_1_ S_3_	2.25/552 2.45/504	0.54 0.92	529 499	103650 70900	596	67/0.26	0.71	2.9	2.21	530	668	138/0.48	2.05
**7 a** (P‐pPh‐P)	S_1_ S_3_	2.26/548 2.49/497	1.07 0.58	531 503	109300 90300	568	37/0.15	0.71	3.2	2.25	545	672	127/0.43	2.02
**7 b** (P‐mPh‐P)	S_3_ S_4_	2.39/520 2.50/496	1.16 0.28	527 500	85700 77900	559	32/0.13	0.71	3.8	2.28	534	638	104/0.38	2.12
**7 c** (P‐oPh‐P)	S_3_ S_4_	2.24/553 2.54/487	0.03 0.83	526 490	65600 77600	564	38/0.16	0.71	5.2	2.27	526	650	124/0.45	2.12
**7 d** (P_3_‐Ph)^[c]^	n.d.	n.d.	n.d.	528 501	96700 81700	558	30/0.13	0.77	3.6	2.28	525	660	135/0.48	2.13
**7 e** (P‐MePh‐P)	S_1_ S_3_	2.40/517 2.45/506	0.70 0.80	525 499	101700 86240	554	29/0.12	0.71	3.6	2.30	525	638	113/0.42	2.17
**7 f** (P‐HexPh‐P)	S_1_ S_3_	2.37/522 2.46/503	0.77 0.72	526 500	94600 81500	556	30/0.13	0.71	3.7	2.29	530	616	86/0.33	2.18
**7 g** (P‐DeOPh‐P)	S_1_ S_3_	2.28/544 2.47/503	0.86 0.63	531 503	103000 85100	575	44/0.18	0.67	3.5	2.25	535	624	89/0.33	2.13

[a] From the intersection between the excitation and emission spectra (in CHCl_3_). [b] From the intersection between the excitation and emission spectra (in film). [c] For this compound computations could not converge; n.d.: not determined.

Frontier molecular orbitals of P‐Ph (**4**) are shown in Figure 1D. The electron density around individual atoms is shown by varying circle sizes, while filled and empty coloring indicate opposite phases (for comparison with isosurfaces, see Figure S2). A nodal plane can be recognized in the central axis of the perylene core, which extends also to the nitrogen of the imide group. Consequently, the substituent on this nitrogen will have a negligible influence on the frontier molecular orbitals and excitation energies. For calculations, we chose to have a methyl group on this position, thereby saving CPU time. In order to estimate the extent to which the phenyl substituent contributes to the frontier molecular orbitals, we used a natural atomic orbital (NAO) analysis using Multiwfn.^[32]^ To this end, we separated the molecules in three fragments ‐ imide group (A), perylene core (B) and phenyl linker (C, if present). Summing of the individual electron density around each atomic center in one fragment allows to estimate its percental contribution to the frontier molecular orbitals of the molecule. In the phenyl substituted compound **4**, the excitation from HOMO to LUMO results in an electron density increase on the imide group (of around 7 % in HOMO to 14 % in LUMO) and a decrease on the perylene core (from 89 % to 84 %, respectively). Unsubstituted P‐H (**1**) shows the same trend (Figure S2). In the HOMO of compound **4**, the phenyl substituent contains only 4 % of the electron density which is further reduced to 2 % in the LUMO. Thus, the phenyl group has a very weak electron donating effect. Since the ortho methyl substitution in compound **5** increases the dihedral angle between the perylene core and the phenyl substituent, it comes as no surprise that the contribution of the phenyl group to the HOMO and LUMO orbitals are even smaller (1.8 % and 1.0 %, respectively, see Figure S2).

The absorption maximum of the perylene monoimide **1** film is more red‐shifted (in comparison to the solution) than the absorption maxima of films made of phenyl substituted monoimides **4** and **5** (Table 1 and Figure S13A). A hindered molecular stacking imposed by these substituents in solid phase might be playing a role here. This is supported by the increased optical band gap of 2.19 eV (determined from the intersection between excitation and emission spectra) for the dimethyl phenyl substituted compound **5**, in comparison to the unsubstituted perylene monoimide **1** (2.15 eV).

Figure 1E contains the computed and via cyclic voltammetry (CV) determined energy levels of compounds **1** and **4**. CV measurements (done on films drop‐casted on a Pt electrode) show that the phenyl substituent slightly upshifts both HOMO and LUMO levels. Computations done on a single molecule (in vacuum) predict the same behavior (Figure 1E). At the same time, if computations are done for two stacked molecules (to resemble CV measurements in film), compound **4** is predicted to have a lower LUMO than compound **1**, which contrasts with CV and DFT on a single molecule. Nevertheless, the differences in absolute numbers are minimal (below 0.1 eV). Thus, for the larger A‐D‐A systems, we used DFT computations for a single molecule. To sum up, introducing a phenyl substituent red‐shifts the absorption maxima, however, they have a small influence on the absorption intensity and energy levels. As a result of the perylene core substitution, the crystallinity is reduced. The computational results for perylene monoimides fit well with the experimental observations, and we turned our attention to the P‐L‐P structures.

### Phenylene‐bridged perylenes (P‐L‐P structures)

#### Geometric considerations

**Figure 2:**
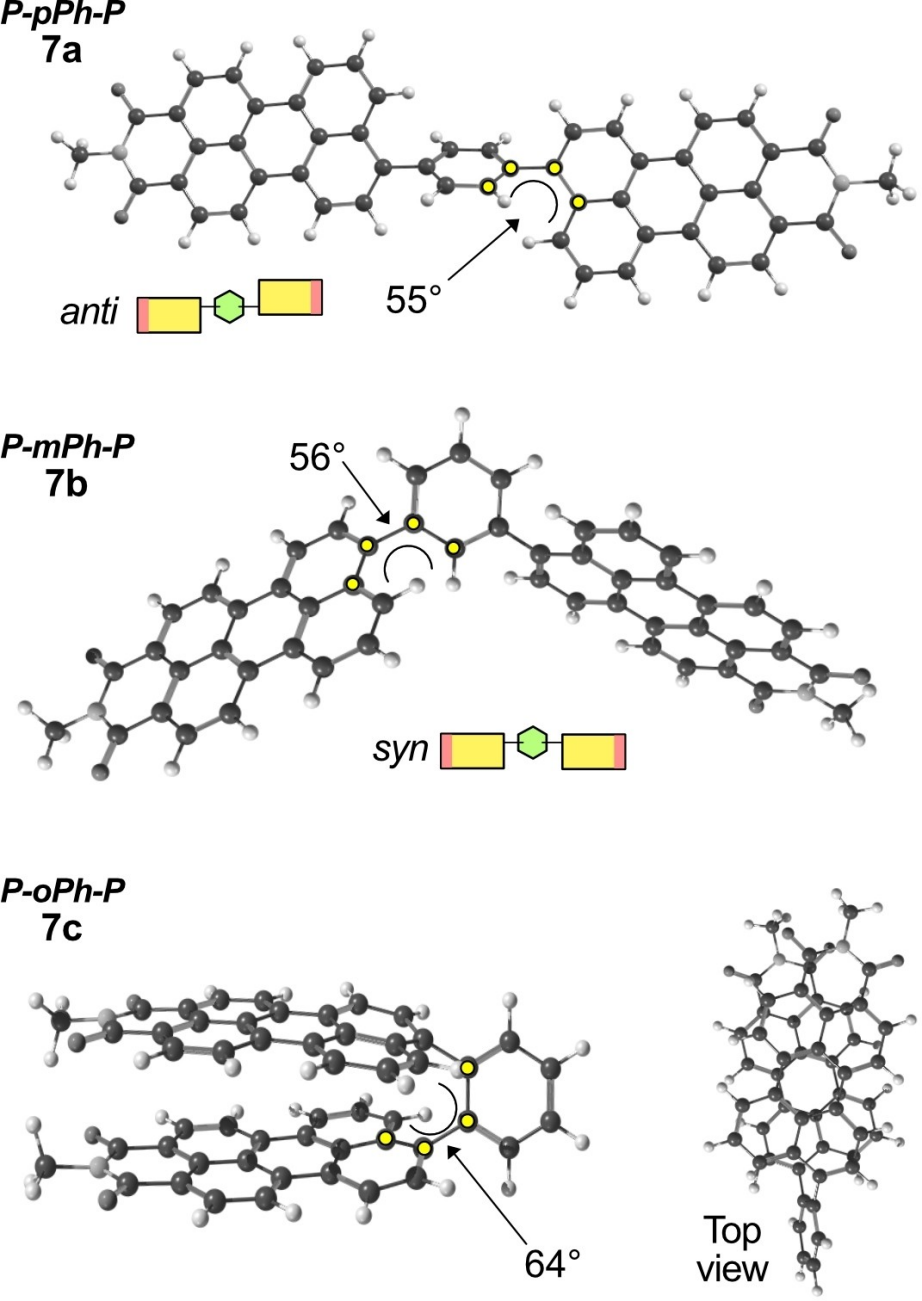
The optimal geometry of para (7a), meta (7b) and ortho (7c) compounds (DFT).

A formation of two conformers, just as described for the monoimides **4** and **5**, is expected for the P‐L‐P acceptors **7 a‐g**. If viewed relative to the phenyl linker, the perylene units can point in the same direction (*syn*) or in opposite directions (*anti*) as schematically shown in Figure 2 (under the structure of **7 a** and **7 b**). For compounds **7 a** and **7 b**, DFT computations indicate that the optimal geometries of both isomers have nearly identical energies (difference being below 1 kJ mol^−1^, see Table S1). However, in case of the ortho isomer **7 c**, the anti‐isomer is predicted to be significantly more stable (by 59 kJ mol^−1^). In the anti‐configuration, the perylene rings can adopt an energetically favorable state in which they are rotationally displaced (by 26°, see the top view in Figure 2).^[37]^ Barriers of interconversion have been determined for benzene with naphthalene substituents in all ring positions: 1,4 (para, 46 kJ mol^−1^);^[38]^ 1,3 (meta, 47 kJ mol^−1^);^[38]^ 1,2 (ortho, 82 kJ mol^−1^)^[39]^ and 1,3,5‐ (trimer, 50 kJ mol^−1^).^[38]^ It can be expected that also for compounds **7 a**–**d**, the rotation barriers are very similar, since the rest of the molecule (second fused naphthalene ring and the imide group) are located far from the C−C bond around which the rotation happens. This is supported by the ^1^H NMR spectra of compounds **7 a**, **7 b** and **7 d**, where a single set of signals is observed (Figure S33). Meanwhile compound **7 c** has very broad signals and for the compounds **7 e**–**g** two sets of signals can be seen due to their slightly higher rotation barriers. At increased temperature (80 °C) NMR signals of **7 f** show signs of coalescence (Figure S35). However, a temperature above 100 °C would be needed to reach interconversion rates high enough for the syn‐ and anti‐isomers to be indistinguishable by NMR spectroscopy, as has been shown for benzene with o‐Me‐naphthyl substituents in the para positions (rotation barrier 82 kJ mol^−1^).^[40]^ Due to the structural likeness of compounds **7 e**–**g** a similar barrier of rotation can be expected, which is in line with the computed value for the rotation of the dimethylphenyl substituent in perylene monoimide **5** (93 kJ mol^−1^, see above). The longer alkyl chains in compounds **7 f**–**g** should have a negligible effect on the rotation due to their flexibility. In fact, the ROESY spectrum of compound **7 f** shows cross peaks between the alkyl chain protons and the perylene core, indicating that in solution they can come in close contact. Summarizing, compounds **7 a**–**g** can exist as two isomers (syn and anti), but these isomers can interconvert at room temperature albeit with different rates. The differences in geometry of these compounds correlate with their solubilities (see below).

### Spectral properties

**Figure 3:**
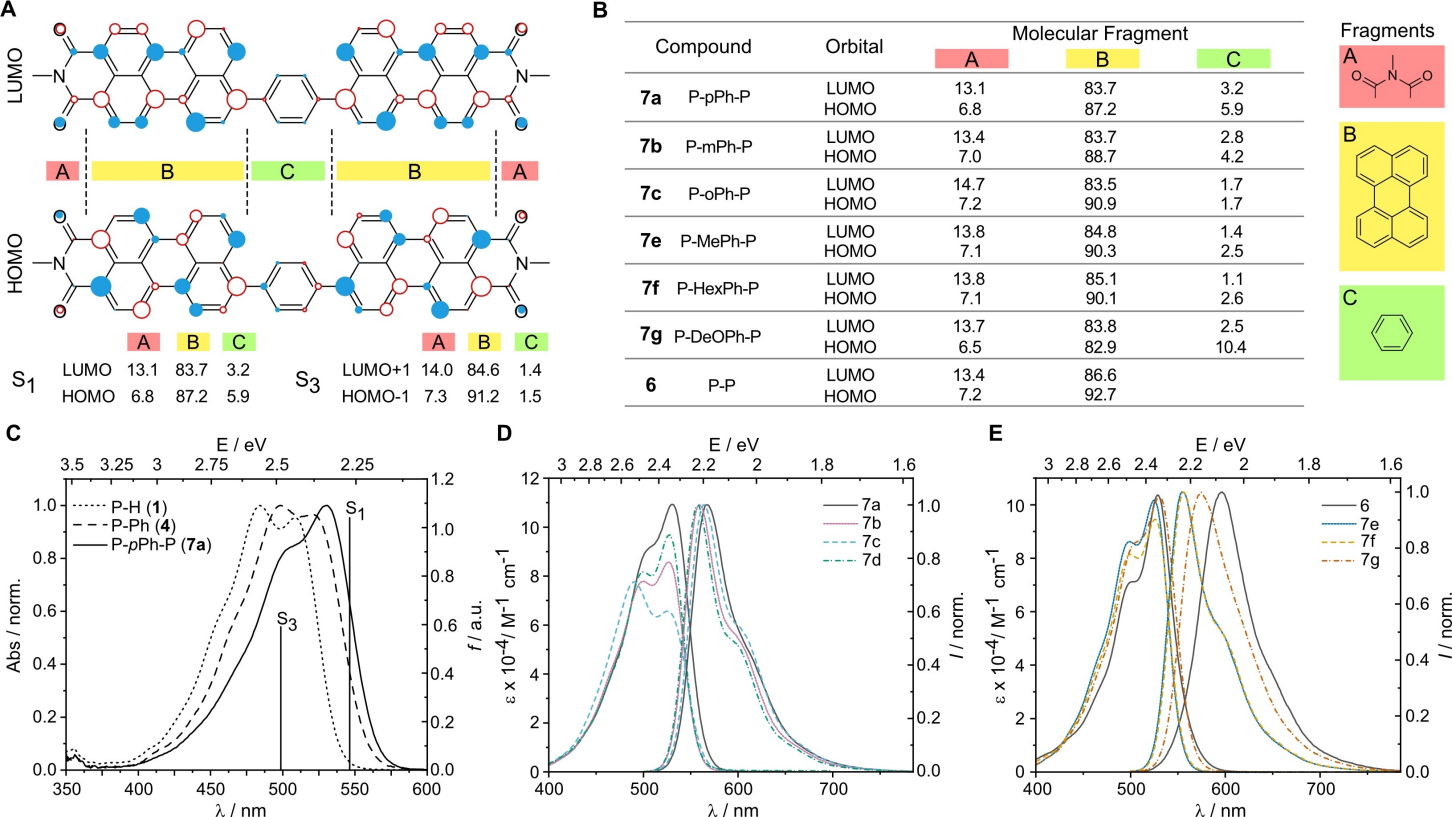
A) LUMO and HOMO of compound 7a (circle size indicates the electron density, filled and empty circles have opposite phases). The percental electronic density on various molecular fragments are given for the S_1_ state. B) The electron density in percent on the phenyl linker for compounds 6, 7a–c,e–g in HOMO and LUMO. C) Absorption spectra of compounds 1, 4 and 7a. D) Absorption and emission spectra of 7a–7d (in CHCl_3_). E) Absorption and emission spectra of 6 and 7e–7f (in CHCl_3_).

Compound **7 a** has a red‐shifted absorption maximum (solid line in Figure 3C) located at 531 nm, if compared to compound **4** (dashed line in Figure 3C), which has only one perylene unit linked to the phenyl group. A large shoulder is located at shorter wavelength (503 nm), which roughly fits to the absorption maximum of the unsubstituted perylene monoimide **1** (dotted line in Figure 3C). According to the computations, the first maximum corresponds to the first excited state S_1_ and the second maximum to S_3_ (S_2_ is a dark state). The S_1_ state is a HOMO‐LUMO transition, while the S_3_ state is a HOMO‐1 to LUMO+1 transition (Figure 3A). Analysis of the electron distribution in these orbitals reveals that the phenyl linker has a weak electron donating character in the S_1_ transition (change from 5.9 % to 3.2 % going from HOMO to LUMO). The imide group gains its electron density not only from the phenyl linker, but also from the perylene core, just as described for compound **4** (see above). In the S_3_ transition the electron density on the phenyl linker practically does not change, but the imide group again withdraws the electron density from the perylene core. Thus, the S_1_ can be assigned to be a charge transfer (CT, phenyl linker and perylene core→imide group) and S_3_ to be a locally excited (LE, perylene core→imide group) state. Since the S_3_ transition is a LE state, it comes as no surprise that the transition energy is similar to the one of perylene monoimide **1** (Table 1). Changing to meta (**7 b**) and ortho (**7 c**) substituted phenyl linkers leads to slightly blue‐shifted absorption maxima and reduced molar absorption coefficients (Figure 3D). The shorter wavelength absorption band (LE state) becomes the dominant one for the ortho compound **7 c**. This can be explained by the reduced conjugation between the phenyl linker and the perylene units, as confirmed by DFT computations (see the calculated electron density distribution in Figure 3B). The electron density on the phenyl linker in HOMO and LUMO is 4.2 % and 2.8 % for the meta (**7 b**) isomer, while for the ortho (**7 c**) isomer it is 1.7 % in both orbitals. However, a more detailed analysis of the excited state electron density and orbital compositions is not feasible for these compounds. This is because syn*‐* and anti‐isomers do not have the same bright and dark states anymore (e. g. for *syn*‐**7 b** the S_1_ is dark, while for *anti*‐**7 b** the S_1_ is a bright state). Furthermore, the states themselves are composed of more complex HOMO‐1, HOMO, LUMO and LUMO+1 transitions in various ratios. Nevertheless, it can be seen that for lower energy states, HOMO to LUMO transitions are dominant, while for higher energy transitions HOMO‐1 and LUMO+1 are dominant. The electron density change on the phenyl linker is larger for the lower energy states, which is in line with the results for **7 a**. For detailed information on excited state energies and contributions, see the Supporting Information (Figures S2–S8). The shape of the absorption spectrum of the trimeric **7 d** resembles that of the meta isomer **7 b**, however the molar absorption coefficient is larger as a result of the additional perylene moiety. For this compound TD‐DFT computations using a basis set with diffuse functions could not converge, but the results without basis set with diffuse functions are similar to **7 b** (Figure S9). Emission spectra of compounds **7 a**–**d** are very similar, with **7 b** and **7 d** having a smaller Stokes shift (32 and 30 nm, respectively) than the para (**7 a**) and ortho (**7 c**) isomers (37 and 38 nm, respectively). These values are very similar to the substituted perylene monoimides **4** and **5** (Stokes shift of 38 and 36 nm). Also, the fluorescence quantum yields are in the same order of magnitude ($\Phi_{Fl}∼$ 0.7). Fluorescence lifetimes of para, meta and trimeric compounds are shorter ($\tau_{Fl}∼$ 3 ns) than those of the perylene monoimides ($\tau_{Fl}∼$ 4 ns), while the longest lifetime was determined for the ortho isomer ($\tau_{Fl}∼$ 5 ns). The longer lifetime of the ortho isomer can be explained by its structural rigidity.^[41]^

Interestingly, compound **6**, which consists of two perylene units connected by a C−C bond (without a phenyl linker) has an absorption spectrum nearly identical to that of **7 a** (Figure 3E). Also, computations show similar S_1_ and S_3_ excited state energies, however, fail to accurately predict the oscillator strength ratio (Table 1). The S_1_ transition is mainly HOMO‐LUMO, with a small contribution from HOMO‐1 to LUMO+1 and in the S_3_ state the same orbitals are involved, but with reversed contribution. However, due to the absence of a phenyl linker, both transitions resemble locally excited states (Figure S8B). Thus, this compound can be viewed as dimeric perylene monoimide. The fluorescence quantum yield of this dimer is in the same order as for other compounds ($\Phi_{Fl}∼$ 0.7), but it has the highest Stokes shift (67 nm) and the shortest fluorescence lifetime ($\tau_{Fl}$ <3 ns) from all the compounds in this study. In a recent paper by Song et al. it was shown that the destiny of molecule **6** in excited state depends on the solvent.^[42]^ In non‐polar solvents (cyclohexane) the angle between both parts of the molecule is reduced (planarization), while in polar solvents (acetonitrile) a symmetry breaking charge transfer is observed (one perylene unit becoming positively charged, while the other becomes negative). The latter process results in a larger Stokes shift. Our measurements are done in chloroform (Stokes shift: 67 nm, Table 2), which in its polarity stand closer to cyclohexane than to acetonitrile (Stokes shifts: 52 nm and 97 nm, respectively).^[42]^ Thus, it could be expected that also in chloroform the dominant excited state process is the planarization with the charge delocalization happening over the entire molecule. All other compounds (**7 a**–**g**) in this study have a smaller Stokes shift than compound **6**, also suggesting that the excitation energy is equally distributed over the entire molecule.^[43]^ The optical properties of compounds **7 e** and **7 f**, which contain methyl and hexyl groups on the phenyl linker (with perylene moieties in para position), are nearly identical since the alkyl chains are not a part of the conjugated π‐system. Thus, also the computation results of both compounds are identical. However, just as could be seen for perylene monoimides **4** and **5**, also here the ortho‐alkyl (ortho in regard to the perylene moieties) substituted compounds are predicted to have a larger dihedral angle between the phenyl linker and the perylene core (Figure S7). Thus, in comparison to the unsubstituted para‐linked **7 a**, methyl and hexyl side chains containing compounds have a higher excitation energy. This can be seen also in the experimental data, as both compounds have blue‐shifted absorption maxima and slightly lower molar absorption coefficients. The Stokes shift for the alkyl substituted compounds is smaller than their unsubstituted analogue **7 a**, probably as a result of the reduced rotational freedom. For both compounds (**7 e** and **7 f**) the S_1_ state corresponds to a HOMO‐LUMO transition, while the S_3_ state is HOMO‐1 to LUMO+1 (S_2_ state being dark). Analysis of the electron density on the phenyl linker reveals that for both compounds S_1_ has a CT character (Figure 3B). These results are identical to compound **7 a**. The situation changes if the substituent on the phenyl linker is a stronger electron donor, as in the case of compound **7 g**, which contains decyloxy groups. The electron donating effect can well be seen if the electron density on the linker is analyzed – in the HOMO it is around 10 %, while in the LUMO it is only 2.5 % (Figure 3B). Since the S_1_ state is mainly a HOMO‐LUMO transition (Figure S8A), it can be concluded that this compound has the strongest CT character from all the compounds in this study. Same as in case of **7 a**, the second transition (S_3_ state) is HOMO‐1 to LUMO+1, which has LE character on the perylene core. Both compounds have near‐identical solution absorption spectra, but the fluorescence spectrum of **7 g** demonstrates a larger Stokes shift than the other A‐D‐A systems in this study. The fluorescence lifetime of all para linked compounds (**7 a**, **7 e**–**g**) is very similar ($\tau_{Fl}∼$ 3 ns), but the fluorescence quantum yield ($\Phi_{Fl}$ <0.7) of **7 g** is slightly lower than those of **7 a**,**e**–**f** ($\Phi_{Fl}$ >0.7).

**Table 2:** Energy levels of compounds 1, 6, 7a‐g.

Compound	CV^[a]^		
	HOMO [eV]	LUMO [eV]	Electrochemical band gap [eV]
**1** (P‐H)	−6.37	−3.99	2.38
**6** (P‐P)	−6.30	−4.03	2.27
**7 a** (P‐pPh‐P)	−6.35	−4.41	1.94
**7 b** (P‐mPh‐P)	−6.29	−3.97	2.32
**7 c** (P‐oPh‐P)	−6.38	−3.97	2.41
**7 d** (P_3_‐Ph)	−6.22	−4.13	2.09
**7 e** (P‐MePh‐P)	−6.36	−3.98	2.38
**7 f** (P‐HexPh‐P)	−6.40	−3.85	2.55
**7 g** (P‐DeOPh‐P)	−6.31	−3.92	2.39

[a] Film measurements. HOMO/LUMO values are obtained from the ox/red potentials and calculated using the following equation: $E_{HOMO/LUMO\;}=\;-\left(4.75+\;E_{onset\;vs.\;NHE}^{ox/red}\right)$ . Measurements were done with freshly casted films for oxidation and reduction potentials each and were calibrated with a Fc/Fc^+^ redox couple (taking Fc/Fc^+^ vs. NHE as 0.64 V).

To conclude, the absorbance bands of all compounds have the same maximum, however, the molar absorbance coefficient value depends on the substitution pattern. Here, the para‐linked compounds, as well as compound **6** have the highest absorption coefficient. Opposite to compound **6**, the presence of a phenyl linker leads to a charge‐transfer character in these molecules, as wanted for A‐D‐A compounds.

Optical measurements of thin films help to elucidate the behavior of the molecules in solid state, which is important for the application in solar cells. For example, a strong bathochromic shift of the absorption maximum is indicative for a head‐to‐tail interaction of the transition dipoles between adjacent molecules (J‐aggregation), while a hypsochromic shift is typical for H‐aggregates (side‐by‐side interaction of the transition dipoles).[^3^, ^44^] The dimeric compound **6** has the same absorption maximum as in solution (530 nm, see Table 1 and Figure S13), suggesting an isotropic distribution of the molecules in the film.^[45]^ This is in contrast with its monomer, perylene monoimide **1**, which had 10 nm red‐shift in film. The introduction of the phenyl linker (**7 a**) gives the molecule more conformational freedom, resulting in a 14 nm red‐shift of the solid state absorption maximum and the smallest optical band gap from the compounds in this study. Adding alkyl substituents on the phenyl linker again disrupts the π‐π interactions in the solid state. As a result, the solid state absorption maxima of compounds **7 b**–**f** are nearly the same as in solution while the optical band gaps, relative to **7 a**, are increased. Thus, the solid state absorption measurements do not indicate the formation of a highly ordered molecular structure.

### GIWAXS characterizations

**Figure 4:**
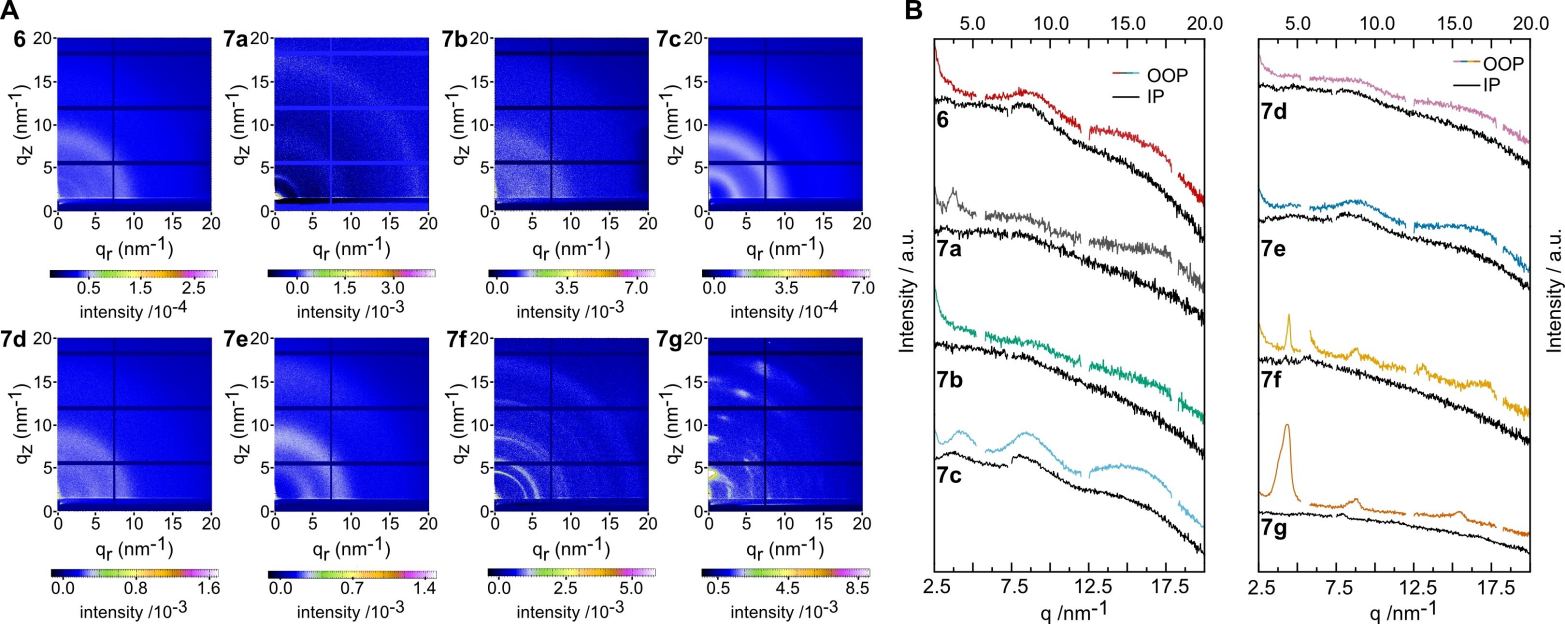
A) 2D GIWAXS patterns of compounds **6**, **7 a**–**g**. B) 1D line‐cuts cuts in the in plane (IP) and out‐of‐plane directions (OOP).

To investigate the crystallinity and molecular packing of the acceptor molecules (**6**, **7 a**–**7 g**), 2D grazing incidence wide angle X‐ray scattering (GIWAXS) measurements of drop casted films have been performed. Contrary to the highly crystalline perylene monoimide (compound **1**, Figure S12), the dimeric P‐P (**6**) shows only weak and diffuse diffraction patterns suggesting a very low crystalline nature. The same observations as for compound **6** can be made for molecules **7 b** and **7 d** (Figure 4). Still, a semicircle‐like feature at about 8.5 nm^−1^ can be recognized, being most presumably the 200 lamellar peak (corresponding to a distance of 0.74 nm). A second feature, which could stem from additional two‐dimensional order of the molecules, is located between 3.5 and 5 nm^−1^, with the position slightly varying in these compounds. The expected π‐π stacking peak is barely recognizable, meaning that only a weak stacking is present between the molecules due to their twisted nature.^[15]^ In compound **7 c** lamellar stacking distances similar to **6**, **7 b** and **7 d** are found. However, the features become much more pronounced. Even though the molecule shows a higher crystallinity, the homogeneous intensity distribution over the semicircles proposes mixed face and edge‐on orientation of the molecules in the thin film.

The para‐substituted compound **7 a** has retained the same diffraction features as the perylene monoimide **1** (Figure S12), although the signal intensity is lower. A semicircular feature can be recognized at 17.5 nm^−1^ indicating a π‐π stacking distance of 0.34 nm. The lamellar d‐spacing distance of compound **7 a** is slightly higher than that of perylene monoimide **1** and its phenyl substituted derivative **4** (peaks at 3.3 nm^−1^ corresponding to a spacing distance of 1.9 nm for **7 a** vs. peaks at 4.6 nm^−1^ corresponding to a spacing distance of 1.4 nm for **1** and **4**). From the other molecules, which contain two perylene monoimide units, only compound **6** has the same d‐spacing distance. In both compounds no preferred orientation is observed. The addition of methyl groups on the phenyl linker (**7 e**) results in a more defined diffraction peak in out‐of‐plane direction disclosing the tendency towards a face on orientation in this sample. Also, this diffraction peak is located at 15.3 nm^−1^, which is lower than for **7 a**, indicating a larger d‐spacing of 0.41 nm. The shifting of the π‐π stacking peak is also observed when compounds **4** and **5** are compared. However, compound **5** shows no favouredorientation. Upon elongation of the alkyl chains (**7 f**) the semicircles are more distinct and have a much higher intensity. However, even though the crystallinity seems to be higher for this molecule, no preferred orientation is observed. When an oxygen atom is implemented in the linker (**7 g**), the GIWAXS pattern reveals isolated features indicating a highly ordered packing. In the out‐of‐plane direction, an intense lamellar diffraction peak at 4.4 nm^−1^, corresponding to a *d*‐spacing of 1.43 nm and higher order lamellar diffraction features are visible. Moreover, a π‐π stacking distance of 0.41 nm^−1^ is revealed. The observed highly ordered packing in **7 g** could be beneficial for vertical charge transport within the absorber layer.

### Electrochemistry

Knowledge about molecular energy levels of new materials is essential for their application as organic semiconductors. To that end, we performed cyclic voltammetry measurements on films and analyzed the obtained values (Table 2) with the aid of the computed data (see also, Figures S14–S24 for cyclic voltammograms and Table S3 for ox/red potentials and DFT computations).

The HOMO levels (derived from the oxidation potentials) of para linked compounds **7 a** and **7 e** are nearly identical to each other and do not differ from the perylene monoimide **1**. If the phenyl linker contains longer hexyl chains (**7 f**), the oxidation potential is slightly higher (lower HOMO). The opposite effect ‐ lowered oxidation potential (upshifted HOMO) can be observed if the linker contains the stronger electron donating decyloxy substituent (**7 g**). Dimeric perylene monoimide **6** has a lower oxidation potential than compound **1**, as has been found also in solution experiments.[^22^, ^46^] The meta linked compound **7 b** has a nearly identical HOMO level to dimeric **6**, while the addition of one more perylene unit (compound **7 d**) upshifts the HOMO level. The energy levels of the ortho linked compound **7 c** are very close to those of perylene monoimide **1**. Ortho substituents in benzene are the least electronically coupled, which might be the reason for the near identical energy levels of both compounds (**7 c** resembling dimeric **1**). Yet, the reduction potential (LUMO level) of the meta substituted compound **7 b** is the same as that of compound **7 c**. If a third perylene units is added to the structure (**7 d**) or the linker is connected in para positions (**7 a**) the reduction potential is lowered (LUMO downshifted). These differences in reduction potentials are likely arising from sterical/film packing effects. A similar order of reduction potential decrease has been found for *p*,*m*,*o*‐terphenyls and 1,3,5‐phenylbenzene (*E*_red_ *p*‐terphenyl<1,3,5‐phenylbenzene<*m*‐terphenyl<*o*‐terphenyl).^[47]^ However, the very low solubility (see below) of compound **7 a** made it difficult to obtain a smooth film for the measurement. Thus, we expect that the significantly lower LUMO level of this compound is a measurement uncertainty. Computations (Table S3) place the LUMO level between the compounds **7 b** and **7 d**. Thus, the real LUMO value probably lies around −4.0 eV (similar to compound **7 e**). Hexyl chains containing compound **7 f** has an upshifted LUMO level when compared to the methyl substituted compound **7 e**. Since both compounds have a nearly identical π‐system (computed HOMO‐LUMO levels of both compounds are the same), experimentally obtained differences are expected to stem from to the film packing effects. In compounds with the discussed P‐L‐P structure, the electronic effects of the linker moiety has a minimal influence on the reduction potential since the first reduction happens by the transfer of two electrons to both perylene units simultaneously.^[22]^ Thus, it comes as no surprise that the electron donating groups on the phenyl linker (compound **7 g**) do not influence the reduction potential much (LUMO is slightly downshifted in comparison to **7 f**, but is still higher than that of **7 e**). Summarizing, energy levels in these P‐L‐P systems are less determined by conjugation and electronic effects than by sterical and film packing properties. In absolute numbers the LUMO and HOMO levels of these compounds only minimally deviate from the parent perylene monoimide (**1**). This can also be seen in the voltages obtained from the solar cell devices (see below).

### Thermal properties and solubility

The thermogravimetric analysis (TGA) shows the high thermal stability of perylene monoimide‐based compounds (Table 3 and TGA plots of all compounds are shown in the Supporting Information, Figures S14–S24). The unsubstituted monoimide **1** has a 5 % weight loss at over 350 °C. When a phenylene or dimethylphenylene (compounds **4** and **5**) is added in peri position, the decomposition begins earlier (166 and 237 °C). At the same time, the phenyl linked **7 a** shows the highest decomposition temperature of 501 °C, followed by **7 e** (489 °C), **7 f** (454 °C) and **7 g** (431 °C). The lower thermal stability of the phenyl substituted monoimides (compounds **4** and **5**) might originate in their asymmetrical structure.^[48]^ With the increasing steric hindrance from the para over meta to ortho coupled compounds, the stability is decreased. Here, the meta isomer **7 b** (492 °C) is similar to the para isomer **7 a**, while the ortho isomer **7 c** is less thermally stable (236 °C). The same trend has been observed for *p*,*m*,*o* triphenyls.^[49]^ Therefore, the trimeric compound **7 d** has the lowest thermal stability (163 °C).

**Table 3:** Solubility in chloroform and toluene and thermal stability.

Compound	Solubility [mg mL^−1^]^[a]^		Thermal stability [°C]^[d]^
	CHCl_3_^[b]^	Toluene^[c]^	
**1** (P‐H)	14	3	355
**4** (P‐Ph)	13	≥9	166
**5** (P‐MePh)	26	≥2	237
**6** (P‐P)	≥26	≥3	482
**7 a** (P‐pPh‐P)	<0.1	<1	501
**7 b** (P‐mPh‐P)	6	<1	492
**7 c** (P‐oPh‐P)	37	1	236
**7 d** (P_3_‐Ph)	≥70	2	163
**7 e** (P‐MePh‐P)	2	<1	489
**7 f** (P‐HexPh‐P)	≥40	6	454
**7 g** (P‐DeOPh‐P)	33	4.5	431

[a] Determined optically, by dilution of saturated solutions. With≥sign indicated samples where solutions appeared to not be completely saturated; [b] 24 °C; [c] 26 °C; [d] Thermal stability defined as the temperature at which a 5 % weight loss is observed in the TGA measurement.

For the application in solar cells, solubility is one of the key issues. The solubility can be increased by structural modifications, such as the introduction of alkyl chains or by increasing the twisting of the molecule. The lowest solubility of all compounds in CHCl_3_ has the para‐phenyl linked **7 a** (Table 3, <0.1 mg mL^−1^). This can be explained by its highly planar structure, which allows it to adopt a strong crystal packingAlready a small hindrance of the planarization (methyl groups in the ortho positions of the phenyl linker) can improve the solubility by a 100‐fold (up to 2 mg mL^−1^). If hexyl chains are used instead, the solubility is more than one order of magnitude larger (≥40 mg mL^−1^). In toluene, compound **7 a** has a more similar solubility to the methyl substituted **7 e**, while the hexyl substituted compound again has the best solubility. Similar solubilities are observed also for **7 g** with dodecyloxy substituents. Increased twisting in the meta and ortho compounds has a positive effect on their solubilities (6 mg mL^−1^ for **7 b** and 37 mg mL^−1^ for **7 c**). The perylene monoimide dimer **6**, which has no phenyl linker, also follows this trend, showing a very high solubility of ≥26 mg mL^−1^. The highest values are reached by the 1,3,5‐substituted trimeric compound **7 d** (≥70 mg mL^−1^).

### Photovoltaic properties

**Figure 5:**
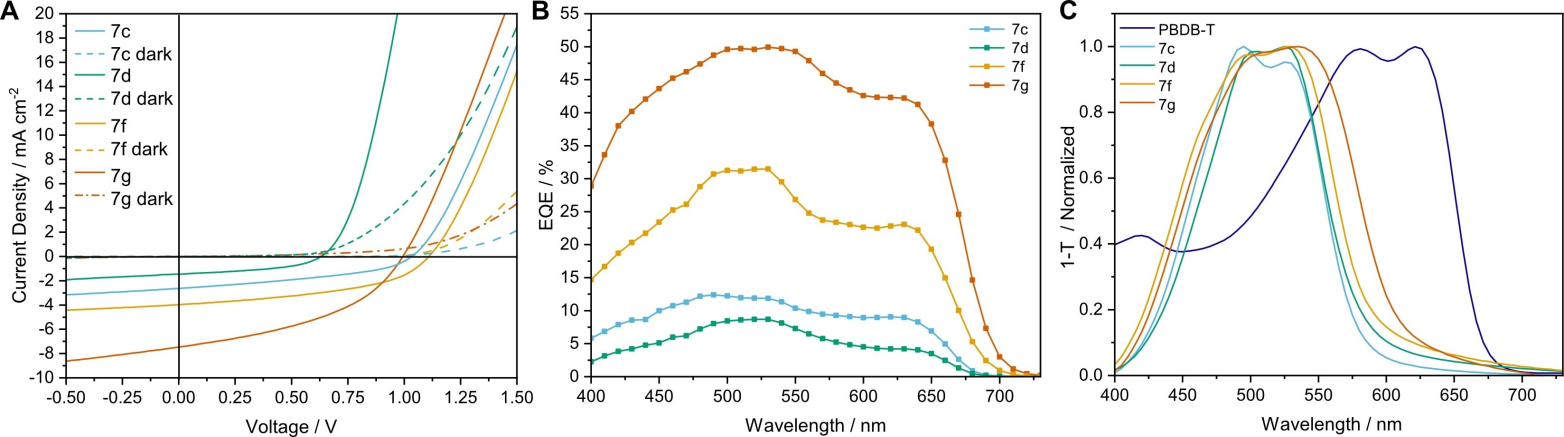
A) *J*–*V* characteristics of solar cells based on compounds 7c, 7d, 7f and 7g. B) EQE spectra of the corresponding blends and C) UV‐Vis film absorption spectra of 7c, 7d, 7f and 7g and PBDB‐T.

Finally, we investigated the photovoltaic performance of the new perylene monoimide based materials using PBDB‐T as donor polymer (for the structural formula see Figure S47). Solar cells with an inverted device structure were built as follows: indium tin oxide (ITO)/zinc oxide (ZnO)/PBDB‐T:acceptor/MoO_3_/Ag. The active layer blends were prepared from solutions in chloroform with a concentration of the donor of 10 mg mL^−1^. The donor was mixed with the acceptor in a weight ratio of 1 : 1. Thermal annealing of the active layer at 150–160 °C (10 min) was advantageous leading to an increased PCE for compounds **7 c**, **7 f** and **7 g**; **7 d** yielded a lower PCE upon annealing (Table S5–8). In Table 4 the device parameters of the average and best solar cells are summarized and typical *J*–*V* curves are shown in Figure 5A. Due to the low solubility (<10 mg mL^−1^) of compounds **7 a**, **7 b** and **7 e**, it was not possible to prepare absorber layers with sufficient thickness and good film quality (see Figure S48) and consequently, their performance in solar cells could not be evaluated.

**Table 4:** Characteristic parameters and absorber layer thicknesses of solar cells based on 7c, 7d, 7f and 7g: PBDB‐T blends; best values in brackets.

Compound	*V*_OC_ [V]	*I*_SC_ [mA cm^−2^]	FF [%]	PCE [%]	Annealing [°C]	Layer Thickness [nm]
**7c** (P‐oPh−P)^[a]^	1.01±0.02 (1.04)	2.38±0.16 (2.62)	41±0.52 (40)	0.98±0.06 (1.08)	150	142±5
**7d** (P_3_‐Ph)^[b]^	0.67±0.03 (0.69)	1.53±0.18 (1.70)	47±0.8 (46)	0.48±0.07 (0.54)	150	140±5
**7f** (P‐HexPh‐P) ^[a]^	1.11±0.01 (1.12)	3.90±0.16 (3.97)	46±0.56 (46)	1.97±0.04 (2.02)	160	140±5
**7g** (P‐DeOPh‐P) ^[a]^	0.99±0.02 (1.00)	6.79±0.47 (7.46)	43±0.55 (43)	2.86±0.23 (3.17)	160	136±5

[a] Average values over 10 solar cells, [b] Average values over 5 solar cells.

The active layer blends based on the investigated novel non‐fullerene acceptors exhibit characteristic large open circuit voltages, reaching values around 1 V. Compound **7 d** is an exception (V_OC_ 0.67 V), which is probably due to its lower thermal stability around the annealing temperature. The V_OC_ of not annealed cells was 0.91 V (Table S4, layer thickness 65 nm). Influence of the active layer morphology most likely plays a role in the reduced V_OC_, since non‐annealed cells with a 144 nm active layer had a V_OC_ of 0.80 V (Table S6). At the same time, thinner solar cells made from **7 c** reached V_OC_ values up to 1.16 V (Table S5). A detailed investigation of the processing conditions and the active layer morphology would be necessary to draw further conclusions about these correlations. Nevertheless, the V_OC_ values reached with these acceptors are comparable to our previous work using the same donor.^[26]^

The photocurrents of compounds **7 c** and **7 d** are similar and both lower compared to para‐linked **7 f** and **7 g**. This might be a consequence of the increased twisting in the ortho and meta configuration (**7 c** and **7 d**, respectively), as increased twisting angles for perylene diimide acceptors are known to have a negative effect on their crystallinity.^[50]^ Reference devices produced from compound **6** showed an increased photocurrent (*I*_SC_=3.42 mA cm^−2^, Table S4 and Figure S49) and had an overall better performance in solar cells than the compounds **7 c** and **7 d**. Pristine films of all three materials had a similar packing (see GIWAXS data in Figure 4), thus the differences in solar cell performance stems most presumably from the interaction with the polymer donor. Here, the linear structure of the compound **6** might be profitable. This is also consistent with the solar cell performance shown by compounds **7 f** and **7 g**. As seen in the GIWAXS data (Figure 4), the more planar geometry of **7 f** and **7 g** enables a better stacking, which is important for charge carrier transport. That yields a higher *J*_SC_ and thus, a higher PCE for solar cells based on these acceptors. The relatively high short circuit current density (7.46 mA cm^−2^) allows compound **7 g** to reach the highest PCE of 3.17 % within this series of acceptors. The EQE spectra (Figure 5B) confirm the same efficiency trend as obtained from the *J*–*V* curves and an equal contribution from the donor and acceptor component can be seen for all devices (see also the film absorption spectra in Figure 5C). The integrated *J*_SC_ values determined from the EQE spectra are consistent with the photocurrent values obtained from the *J*–*V* curves (integrated J_SC_ 1.64, 0.95, 4.22 and 7.73 mA cm^−2^ for compounds **7 c**, **d**, **f**, **g** respectively). The reduced values for the compounds **7 c** and **7 d** are probably due to the instability of the solar cells in air during the EQE measurements.^[26]^

The performance of these P‐L‐P acceptors in solar cells allows making the following conclusions. The increased electron donating strength of the phenyl linker (“L”) has a positive impact, as shown by the best performing compound **7 g**. According to DFT computations, it also has the strongest charge transfer character from the phenyl linker to the imide groups. Moreover, the increasing PCE values (**7 c**<**f**<**g**) correlate well with the increasing calculated electron donating strength of the phenyl linker in the various compounds (see Figure 3B). To that end, if the electron donating character of the phenyl linker is close to none (ortho substituted **7 c**), the morphological properties of the acceptor become more important. This is demonstrated by the control device built with compound **6** (no phenyl linker, more linear structure), which had a similar performance to compounds **7 c** and **7 d**. Thus, elongation of the molecule with a para substituted phenyl linker that also contains alkyl chains (compounds **7 f** and **7 g**) is advantageous to the solar cell performance. This is due to a combination of two factors, the stronger electron donating character of the linker and an improved morphology.

## Conclusion

In this study, a detailed structure–property analysis of P‐L‐P dyes has been performed. These dyes were prepared using Suzuki reaction. Even though side reactions of the Suzuki coupling made the purification challenging in some cases, all compounds could be obtained in reasonable yields. In comparison to perylene monoimide, the P‐L‐P dyes have a red‐shifted absorption (ca. 20 nm) and a significantly increased molar absorption coefficient. Changing of the linker's substitution pattern (para, ortho, meta) allows a fine‐tuning of the spectral and electrochemical properties of these materials. Alterations in the molecular structure also influences the crystallinity of the compounds, thus their solubilities and thermal stabilities vary greatly. The relatively low reduction potentials of these molecules makes them suitable to be used as non‐fullerene acceptors in organic photovoltaics and thus solar cells based on absorber layers containing PBDB‐T as donor and the synthesized compounds as acceptor were investigated. A clear correlation of the linker electron donating strength (determined by DFT computations and NAO analysis) and device efficiencies could be recognized. The best performing solar cells were made from compounds where the perylene monoimides were in para positions. The highest power conversion efficiency (3.17 %) was achieved with the compound based on the strongest electron donating linker used in this study, the decyloxy substituted benzene. Overall, the results obtained in this study provide guidelines for tailoring perylene based P‐L‐P dyes for specific applications.

## Experimental Section

Detailed description of experimental procedures is provided in the Supporting Information.^[51]^

Deposition Numbers 2144711 (for 1), 2144712 (for 4) contain the supplementary crystallographic data for this paper. These data are provided free of charge by the joint Cambridge Crystallographic Data Centre and Fachinformationszentrum Karlsruhe Access Structures service.

## Conflict of interest

The authors declare no conflict of interest.

## Supporting information

As a service to our authors and readers, this journal provides supporting information supplied by the authors. Such materials are peer reviewed and may be re‐organized for online delivery, but are not copy‐edited or typeset. Technical support issues arising from supporting information (other than missing files) should be addressed to the authors.

Supporting Information
