# Supplementary material for: Phenylene‐Bridged Perylene Monoimides as Acceptors for Organic Solar Cells: A Study on the Structure–Property Relationship
Source: Chemistry. 2022 Mar 19;28(23):e202200276. doi: 10.1002/chem.202200276 (PMC9313791; doi:10.1002/chem.202200276)
Supplement: Supplementary file 1 — Supporting Information [file CHEM-28-0-s001.pdf]

Chemistry–A European Journal

Supporting Information

Phenylene-Bridged Perylene Monoimides as Acceptors for Organic Solar Cells: A Study on the Structure–Property Relationship

Bettina Schweda, Matiss Reinfelds,* Jakob Hofinger, Georg Bäumel, Thomas Rath, Petra Kaschnitz, Roland C. Fischer, Michaela Flock, Heinz Amenitsch, Markus Clark Scharber, and Gregor Trimmel*

Table of Contents

S1. General information 2

S2. DFT computations 4

Benchmarking 4

Result summary 4

S3. Crystal structure data 14

S4. GIWAXS 17

S5. Optical, thermal and electrochemical data 18

Electrochemical properties 30

S6. Synthesis procedures 30

Perylene monoimide building block 30

Suzuki couplings 32

S7. NMR spectra 37

S8. Solar cell fabrication and characterization 58

Device Optimization 59

References 61

S1. General information

Materials and purification: Reagents and solvents were purchased from Merck, Acros, TCI, abcr, Fluorochem and Fisher Scientific and used as received. The polymer for solar cell production - PBDB-T (poly[(2,6-(4,8-bis(5-(2-ethylhexyl)thiophen-2-yl)-benzo[1,2-b:4,5-b']dithiophene))-alt-(5,5-(1',3'-di-2-thienyl-5',7'-bis(2-ethylhexyl)benzo[1',2'-c:4',5'-c']dithiophene-4,8-dione)], CAS: 1415929-80-4) was purchased from 1-material. TLC was done on TLC Silica gel 60 F_{254} (aluminium sheets, Merck). Column chromatography was done on self-filled columns (Macherey Nagel silica gel 0.04-0.063 mm) using the "Selekt" flash chromatography instrument (Biotage).

NMR spectra were measured on 300 MHz Bruker Avance III and 500 MHz Varian INOVA in $CDCl_3$ and Toluene-d8. Spectra were referenced to solvent peaks: ^{1}H 7.26 and ^{13}C 77.16 for $CDCl_3$; ^{1}H 2.08 (methyl group) for Toluene-d8. NOESY1D (transient NOE) experiments were used, in which the selective inversion pulse has been replaced by a DPFGSE inversion sequence.

High resolution mass spectra (HRMS) were recorded on the 'Micromass MALDI micro MX' mass spectrometer from Waters. The matrix was Dithranol or trans-2-[3-(4-tert-Butylphenyl)-2-methyl-2-propenylidene]malononitrile (DCTB) with a concentration of 10 mg mL^{-1} in THF; the sample had a concentration of 1 10 mg mL^{-1} in DCM in a mixing ratio matrix/sample = 7/2. The reference material used was polyethylene glycol (PEG). The data analysis was done with the MassLynx V4.1 software.

Thermogravimetric analysis (TGA) was done on the 'STA 449 C' from NETZSCH. The samples were measured in an aluminum pan with helium as protective gas. The flow rate was 50 mL min^{-1} and the heating rate 10 K min^{-1}.

The **determination of solubility** was done via optical spectroscopy. To that end, a saturated solution was prepared by stirring the sample in 100 µL of the corresponding solvent for 1 h (at room temperature). Then the sample was filtered using syringe filter (PTFE membrane, pore size 0.41 µm, 4 mm diameter). At least 30 µL of saturated solution could be obtained this way. Without any further delay, 10 µL of this solution was transferred to 15 mL of $CHCl_3$. The concentration of the sample was then determined (using the molar absorption coefficient) by UV-Vis spectrometer and this value was used to calculate the solubility. In case of very well soluble compounds (e.g. P-P, **6**) the obtained values should be viewed as the lower border of the solubility as it was difficult to unambiguously determine (visually) whether undissolved particles are still present in the vial (during preparation of the saturated solution). In this case the values in the table are denoted with ≥.

Cyclic voltammetry (CV) measurements were performed using Jaissle Potentiostat–Galvanostat IMP 88 PC-100. A three-electrode setup was used where two Pt-plates served as working and counter electrodes and Ag/AgCl wire as reference electrode. The materials were drop casted on the working electrode and measured using 0.1 M tetrabutylammoniumhexafluorophosphate ($TBAPF_6$) in acetonitrile (MeCN) as the electrolyte solution (in nitrogen filled glovebox, with scan speed of 50 mV s^{-1}). Two measurements were done: one starting from zero to positive voltages, the other one starting from zero to negative voltages. For each measurement a freshly casted film was used. Calibration was done using Fc/Fc^+ redox couple. The HOMO and LUMO energy levels were calculated using the following equation: $E_{HOMO/LUMO} = -(4.75 + E^{ox/red}_{onset\ vs.\ NHE})$,[1] taking Fc/Fc^+ vs. NHE as 0.64 V.[2]

Optical measurements in $CHCl_3$ solution were done using the Shimadzu spectrophotometer UV-1800 (UV-Vis); Fluorescence (excitation and emission spectra) measurements were done using the F-7000 FL Spectrophotometer (Hitachi). Luminescence lifetimes were determined using time-correlated single

photon counting on a FluoroLog 3 spectrofluorometer (Horiba Scientific) equipped with an NIR-sensitive R2658 photomultiplier (Hamamatsu) and DeltaHub module controlling a NanoLED (456 nm) laser diode. Lifetimes were calculated using the monoexponential decay model (although every data set was controlled also with biexponential decay model) in OriginPro 2021. The relative quantum yields were determined on the same spectrofluorometer and were calculated against the standard 'Fluoreszenzorange' from Kremer Pigmente (perylene diimide with $\Phi_{\mathrm{fl}} = 0.95$). Film measurements were done with Lambda 1050 double-beam UV-Vis-NIR and Shimadzu UV-1800 spectrometers, fluorescence excitation and emission spectra with PTI QuantaMaster 40 device.

Optical microscopy was done using the light microscope 'BX60' from Olymp with a camera (Olymp) attached. The active layer was magnified 200x and 500x.

Grazing Incidence Wide Angle X-ray Scattering (GIWAXS): Samples for the measurements were prepared via drop coating from chloroform solutions on silicon substrates followed by an annealing step of 10 min at 100 °C in inert atmosphere. The measurements were carried out at the Austrian SAXS Beamline 5.2L of the electron storage ring ELETTRA Trieste at a photon energy of 8 keV. For the detection of the GIWAXS images, a Dectris Pilatus3 1M detector was used set to a sample detector distance of 294 mm. The angular calibration of the detector was carried out using silver behenate powder (d-spacing: 58.38 Å). All measurements have been performed with a grazing angle of 1.1°. In order to enhance the visibility of the weak diffraction the image a blank wafer has been subtracted. The in-plane line-cuts taken at the Yoneda wing (qr) and out-of-plane line-cuts (qz) along the Ewald sphere have been determined with the automatic data processing software SAXSDOG.[3] The images were processed using the IGOR Pro 4.32, Wavemetrics and FIT2D.

Single-crystal X-ray Diffraction: For single crystal X-ray diffractometry suitable crystals were covered with a layer of silicone oil. Under a microscope a single crystal was selected, mounted on a glass rod on a copper pin, and placed in the cold N_2 stream provided by an Oxford Cryosystems cryometer (T=100 K). XRD data collection was performed on a Bruker APEX II[4] diffractometer with use of Mo Kα radiation (λ= 0.71073 Å) from an IµS microsource and an APEX II CCD area detector. Data integration was carried out using SAINT.[4] Empirical absorption corrections were applied using SADABS.[5]
The structure was solved by the dual space algorithm implemented in SHELXT.[6] Fourier analysis and refinement were performed by the full-matrix least-squares methods based on F^2 as implemented in SHELXLE.[7]
The space group assignments and structural solutions were checked and evaluated using PLATON.[8]
All non-hydrogen atoms were refined anisotropically. All hydrogen atoms were placed in calculated positions corresponding to standard bond lengths and angles using riding models. CIF files were edited, validated and formatted with the programs encifer[9] and Olex2.[10]
Structural plots and figures were generated with MERCURY.[11]

S2. DFT computations

Benchmarking

A

Functional	Basis Set	HOMO	LUMO	Bandgap
wB97XD	6-31G(d,p)	-7.26	-1.23	-6.02
M062X		-6.66	-2.09	-4.57
CAM-B3LYP		-6.67	-1.73	-4.94
B3LYP ($CHCl_3$)		-5.51	-2.81	-2.69
PBE0		-5.77	-2.75	-3.02
B3LYP		-5.52	-2.79	-2.73
B3LYP (GD3)		**-5.52**	**-2.79**	**-2.73**
B3LYP (dimer, GD3)		-5.43	-2.76	-2.67
PBE0	cc-pVDZ	-5.88	-2.88	-3.00
	def2-TZVP	-5.97	-2.98	-2.99
B3LYP	cc-pVDZ	-5.65	-2.94	-2.71
	def2-TZVP	-5.80	-3.10	-2.70
CV (film)		-6.37	-3.99	-2.38

B

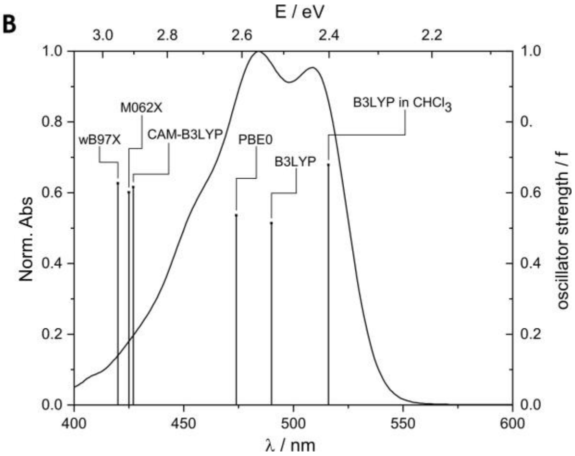

Figure S1: Calculated and experimental results for P-H **1** (for calculations, methyl group was used on the imide nitrogen). *A* – energy levels as calculated by various functionals and basis sets, CV result (measured in thin film). *B* – energy of the S_0-S_1 transition and corresponding oscillator strength for the indicated functionals, using 6-31G+(d,p) basis set.

Choice of functional and basis set:
Range separated hybrid functionals like CAM-B3LYP, wB97XD and global hybrid functional M062X together with 6-31G(d,p) basis set yields overestimated the HOMO-LUMO band gaps (Figure S1A; experimental result from CV at the bottom of the table), as well as overestimated excitation energies (Figure S1B). At the same time, hybrid functionals – PBE0 and B3LYP provide more realistic results. With these two functionals also other basis sets were tested e.g. Dunnings double zeta cc-pVDZ and Karlsruhe triple zeta def2-TZVP. In both cases the results are very similar to those obtained with Pople's double zeta 6-31G(d,p) basis set. Computation times with Pople and Dunnings basis sets were comparable, while Karlsruhe basis sets needed significantly (15x) more time. Somewhat increased calculation time is not surprising, since the def2-TZVP is a triple zeta basis set. However, also for the TD-DFT computations the def2-TZVP basis set needed more time than the triple zeta 6-311G+(d,p) basis set (36 h vs. 11 h using the B3LYP functional). Qualitatively both basis sets provide very similar results. The excitation energies obtained with B3LYP (and basis sets with diffuse functions) overlays better with the experimental absorption spectrum of P-H (**1**), thus for all other computations we choose to use the B3LYP functional with 6-31G(d,p) basis set for geometry optimization, rotational energy scan and NAO analysis, and 6-31G+(d,p) basis set for TD-DFT calculations (including vibrationally resolved absorption spectrum). Furthermore, an empirical dispersion correction is added (GD3) to account for π- π interactions which is expected for the compounds in this study.

Result summary

In the following figures a summary of the most important computational results are given for each molecule. Figures contain the molecular structure and, for compounds, which have a phenyl substituent/linker, the dihedral angle between the perylene core and the substituent/linker is indicated (from geometry optimization in B3LYP-GD3/6-31G(d,p) level of theory).

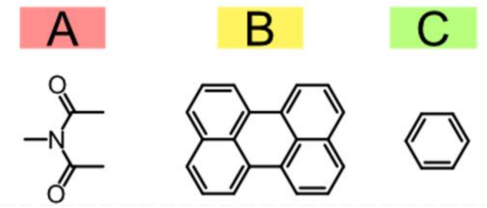

In order to elucidate the electronic contribution of the phenyl linker to the acceptor-donor-acceptor system (where the donor is a phenyl linker and the acceptor is perylene monoimide) we *separated* the molecules into three parts: the imide group (part A, red), perylene core (part B, yellow) and phenyl substituent/linker (part C, green). The percentual distribution of the electron density (natural atomic orbitals) of these three parts are provided for the relevant molecular orbitals. Molecular orbitals are indicated by "H" for HOMO, by "L" for LUMO and by an additional number for higher and lower orbitals (e.g. "H-1" meaning HOMO-1). Also the isosurfaces and the orbital energies are provided (all calculated using B3LYP-GD3/6-31G(d,p) level of theory). Finally, a summary of TD-DFT computation results (computed on a B3LYP-GD3/6-31G+(d,p) level of theory) are provided in the following table. For TD-DFT computations first 10 excited states are calculated, and with few exceptions, only the bright excited states are reported (with oscillator strengths above 0.1).

Table S1: An energy comparison for the optimized geometry for compounds **7a-c,e,g**, anti- and syn-isomers. For a comparison, also results without the empirical dispersion correction are shown.

Compound	Empirical dispersion for geometry optimization	Isomer		Δ kJ mol^{-1}
		syn (Hartree)	anti (Hartree)	
P-pPh-P (**7a**)	no	-2408.4004260	-2408.4006400	0.56
	yes	-2408.4979370	-2408.4980410	0.27
P-mPh-P (**7b**)	no	-2408.400134	-2408.400255	0.32
	yes	-2408.498086	-2408.497954	0.35
P-oPh-P (**7c**)	no	-2408.395605	-2408.395023	1.53
	yes	-2408.501262	-2408.52387	59.36
P-MePh-P (**7e**)	no	-2486.983107	-2486.983271	0.43
	yes	-2487.090807	-2487.090880	0.19
P-DeOPh-P (**7g**)	no	-2637.380054	-2637.379893	0.42
	yes	-2637.489776	-2637.489821	0.12

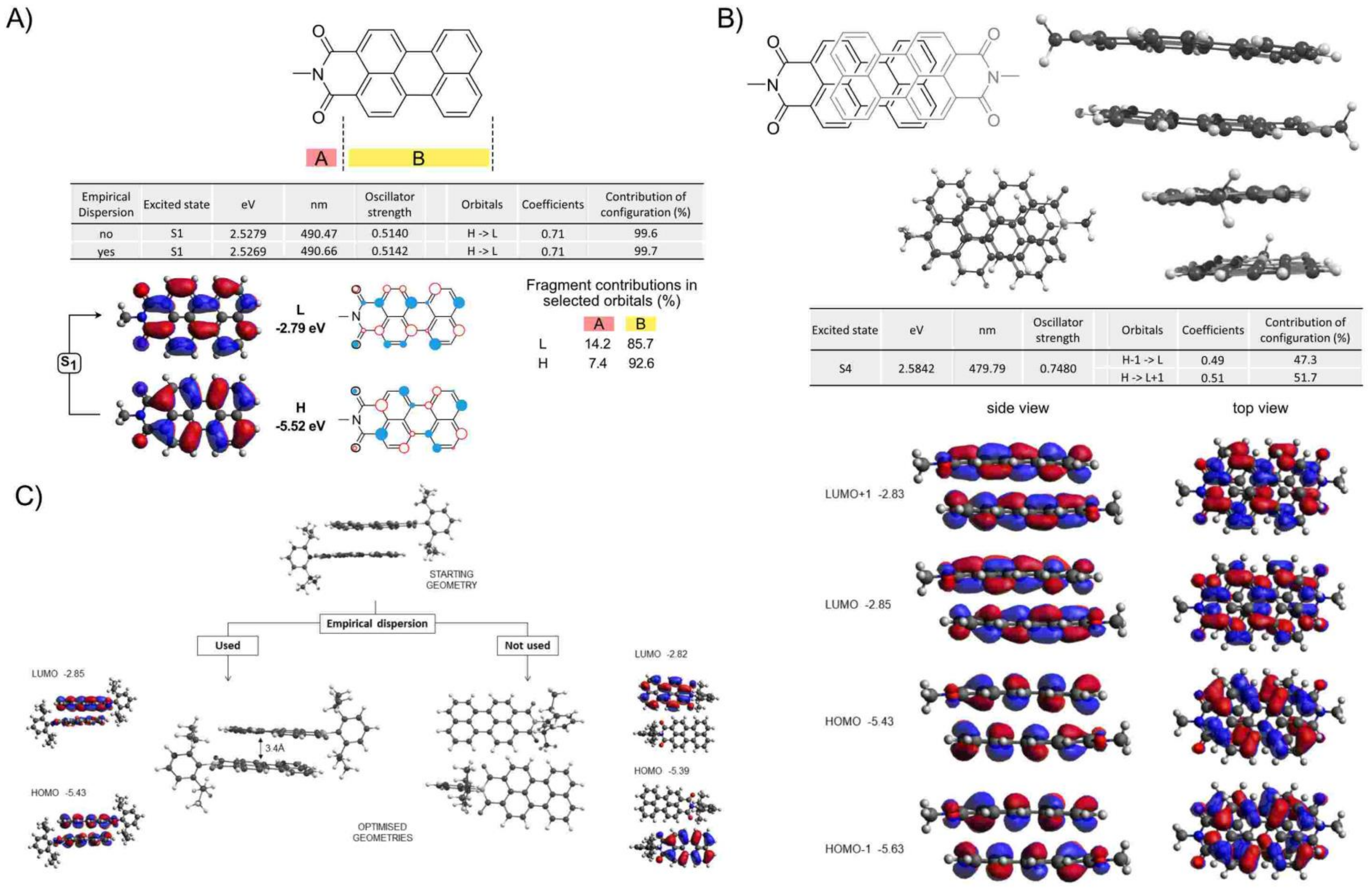

Figure S2: Computation data of perylene monoimide (P-H, compound **1**): *A* - monomer; *B* – stack of two monomers; *C* – Stack of two monomers with a full substituent on the imide nitrogen. In the A and C also results obtained without the use of the empirical dispersion correction are displayed.

A)

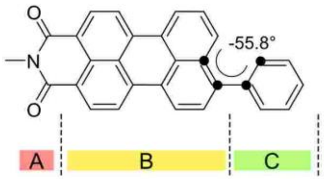

Isomer	Empirical Dispersion	Excited state	eV	nm	Oscillator strength	Orbitals	Coefficients	Contribution of configuration (%)
left	no	S1	2.4423	507,66	0.6708	H -> L	0.71	99.6
left	yes	S1	2.4390	508.34	0.6725	H -> L	0.71	99.6
right	yes	S1	2.4385	508.44	0.6727	H -> L	0.71	99.6

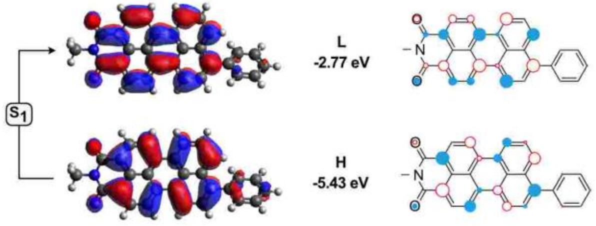

Fragment contributions in selected orbitals (%)

	A	B	C
L	13.9	84.0	2.1
H	7.1	88.8	4.1

B)

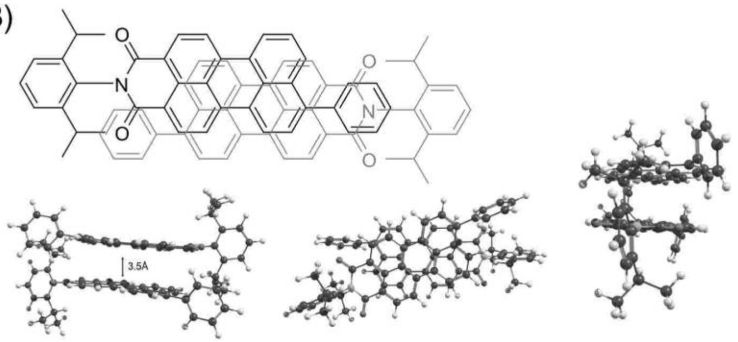

Excited state	eV	nm	Oscillator strength	Orbitals	Coefficients	Contribution of configuration (%)
S4	2.5081	494.34	1.1120	H-1 -> L	0.46	42.3
				H -> L+1	0.53	56.7

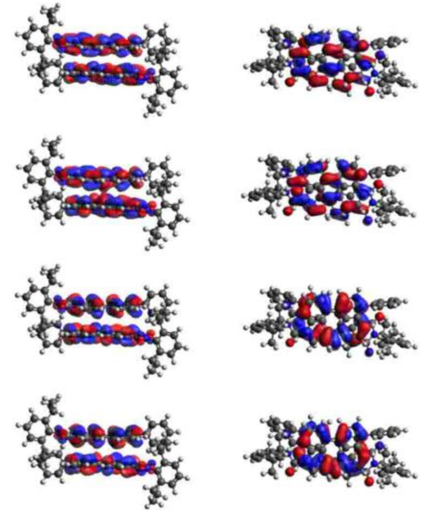

C)

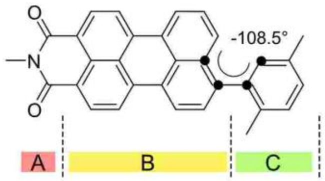

Isomer	Empirical Dispersion	Excited state	eV	nm	Oscillator strength	Orbitals	Coefficients	Contribution of configuration (%)
right	no	S1	2.4780	500.33	0.6401	H -> L	0.71	99.6
right	yes	S1	2.4712	501.72	0.6459	H -> L	0.71	99.5
left	yes	S1	2.4712	501.72	0.6459	H -> L	0.71	99.5

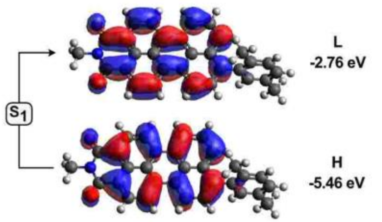

Fragment contributions in selected orbitals (%)

	A	B	C
L	14.1	84.8	1.0
H	7.2	90.9	1.8

Figure S3: Computation data of P-Ph (**4**) monomer (part A) and dimer (part B); *C* – P-MePh (**5**). In the A and C also results obtained without the use of the empirical dispersion correction are displayed.

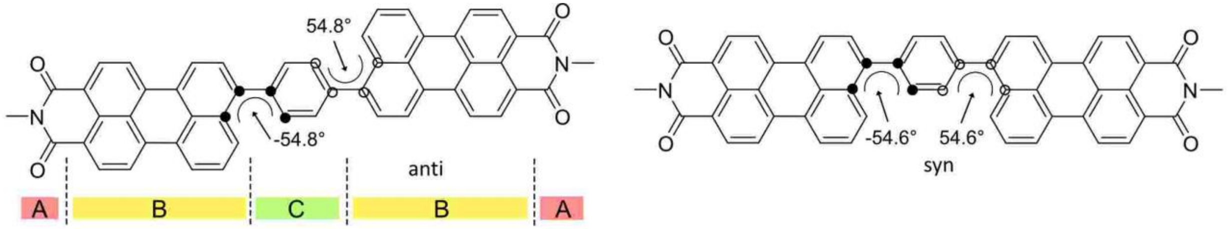

Conformation	Empirical Dispersion	Excited state	eV	nm	Oscillator strength	Orbitals	Coefficients	Contribution of configuration (%)
anti	no	S1	2.2708	546,00	1.0572	H -> L	0.71	99.5
		S3	2.4953	496.86	0.5834	H-1 -> L-1	0.71	99.6
syn	no	S1	2.2713	545.88	1.0405	H -> L	0.71	99.5
		S3	2.4879	498.35	0.5908	H-1 -> L-1	0.71	99.6
anti	yes	S1	2.2636	547.73	1.0667	H -> L	0.71	99.5
		S3	2.4956	496.82	0.5796	H-1 -> L-1	0.71	99.6
syn	yes	S1	2.2647	547.47	1.0476	H -> L	0.71	99.5
		S3	2.4876	498.41	0.5893	H-1 -> L-1	0.71	99.6

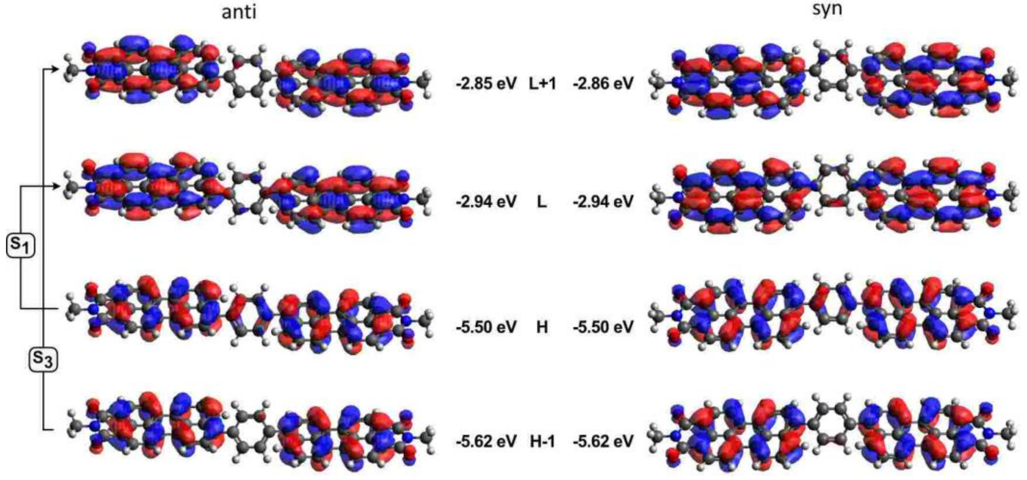

Fragment contributions in selected orbitals (anti isomer) (%)

	A	B	C
L+1	14.0	84.6	1.4
L	13.1	83.7	3.2
H	6.8	87.2	5.9
H-1	7.3	91.2	1.5

Figure S4: Computation data of P-pPh-P (**7a**). A comparison of syn- and anti-isomers. In table also results obtained without the use of the empirical dispersion correction are displayed.

Conformation	Excited state	eV	nm	Oscillator strength	Orbitals	Coefficients	Contribution of configuration (%)
syn	S1	2.3436	529.03	0.0210	H-1 -> L	0.25	12.5
					H -> L	0.58	66.4
					H -> L+1	-0.31	19.4
	S3	2.3850	519.86	1.1635	H-1 -> L	0.40	31.6
					H-1 -> L+1	-0.19	7.1
					H -> L+1	0.54	59.0
	S4	2.5015	495.63	0.2832	H-1 -> L	-0.28	15.8
					H-1 -> L+1	0.45	40.8
					H -> L	0.35	24.7
					H -> L+1	0.30	18.3

Conformation	Excited state	eV	nm	Oscillator strength	Orbitals	Coefficients	Contribution of configuration (%)
anti	S1	2.3503	527.51	0.3770	H-1 -> L+1	0.12	3.0
					H -> L	0.54	58.7
					H -> L+1	0.43	36.5
	S3	2.4031	515.93	0.8524	H-1 -> L	- 0.39	30.5
					H-1 -> L+1	0.57	63.9
					H -> L	- 0.16	4.9
	S4	2.5026	495.42	0.2601	H-1 -> L	0.44	38.8
					H-1 -> L+1	0.35	24.8
					H -> L	0.26	13.0
					H -> L+1	- 0.34	22.9

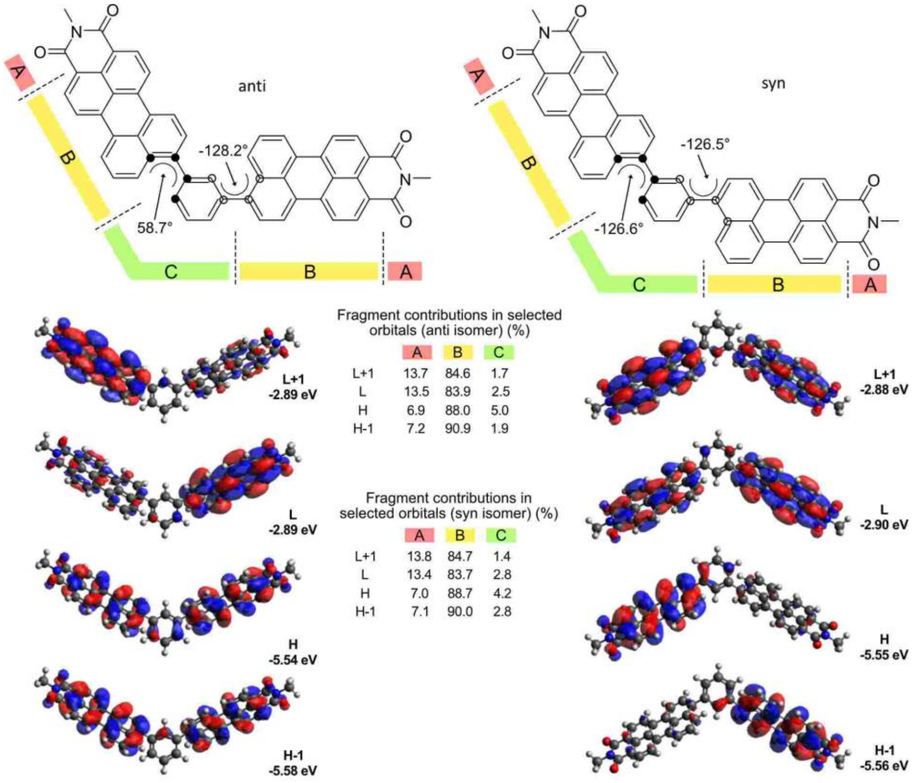

Figure S5: Computation data of P-mPh-P (**7b**). A comparison of syn- and anti-isomers.

Conformation	Excited state	eV	nm	Oscillator strength	Orbitals	Coefficients	Contribution of configuration (%)
syn	S1	2.1744	570.20	0.0432	H-1 -> L	-0.21	8.9
					H-1 -> L+1	0.15	4.2
					H -> L	0.65	84.2
					H -> L+1	-0.11	2.3
	S3	2.3824	520.42	0.2736	H-1 -> L	-0.21	9.0
					H-1 -> L+1	0.62	76.2
					H -> L	-0.23	10.6
					H -> L+1	-0.14	3.9
	S4	2.5213	491.75	0.8128	H-1 -> L	0.53	56.9
					H-1 -> L+1	0.11	2.5
					H -> L+1	-0.44	38.7

Conformation	Excited state	eV	nm	Oscillator strength	Orbitals	Coefficients	Contribution of configuration (%)
anti	S1	2.0580	602.45	0.0003	H-1 -> L	0.57	63.8
					H -> L+1	-0.42	36.0
	S3	2.2411	553.24	0.0259	H-1 -> L	0.42	35.8
					H -> L+1	0.56	63.7
	S4	2.5441	487.35	0.8302	H-1 -> L+1	0.51	52.3
					H -> L	0.48	46.5

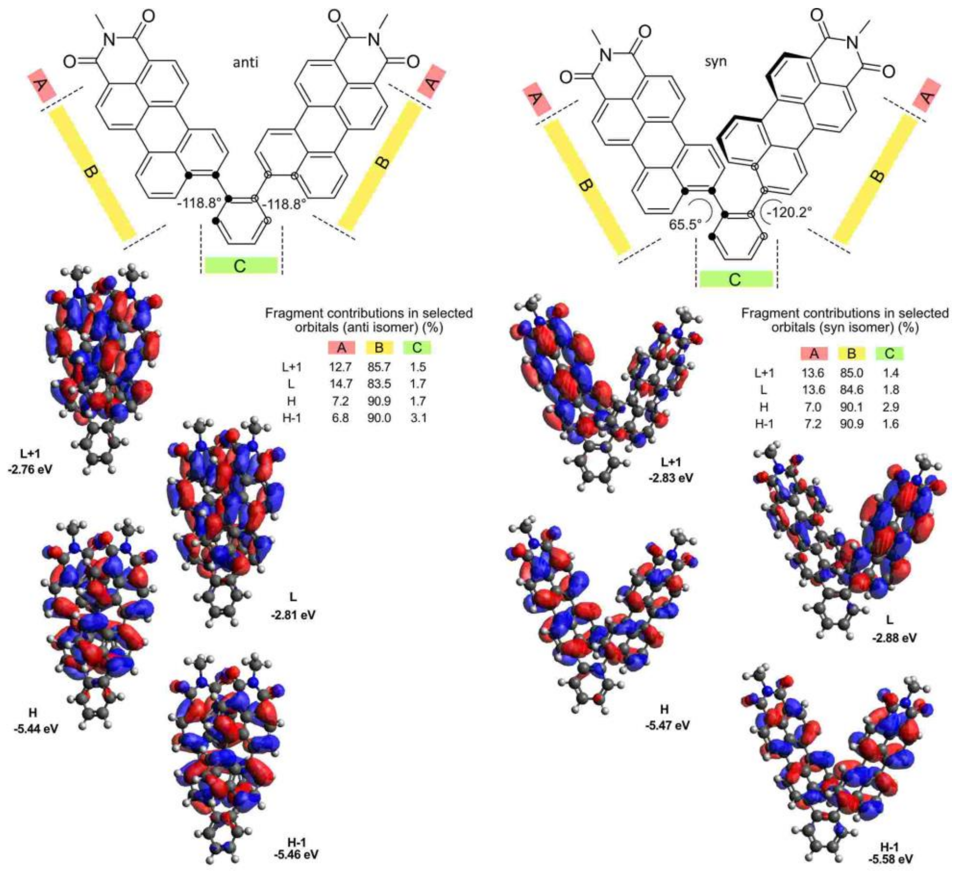

Figure S6: Computation data of P-oPh-P (**7c**). A comparison of syn- and anti-isomers.

A)

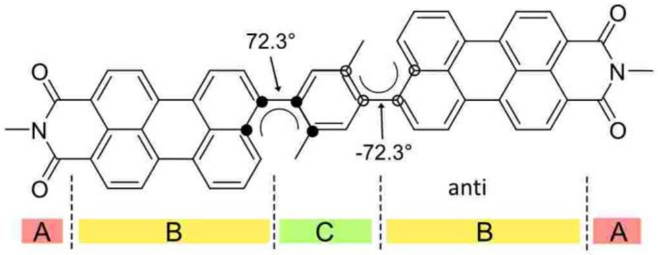

Excited state	eV	nm	Oscillator strength	Orbitals	Coefficients	Contribution of configuration (%)
S1	2.3977	517.09	0.7030	H -> L	0.70	99.1
S3	2.4490	506.26	0.8044	H-1 -> L-1	0.71	99.3

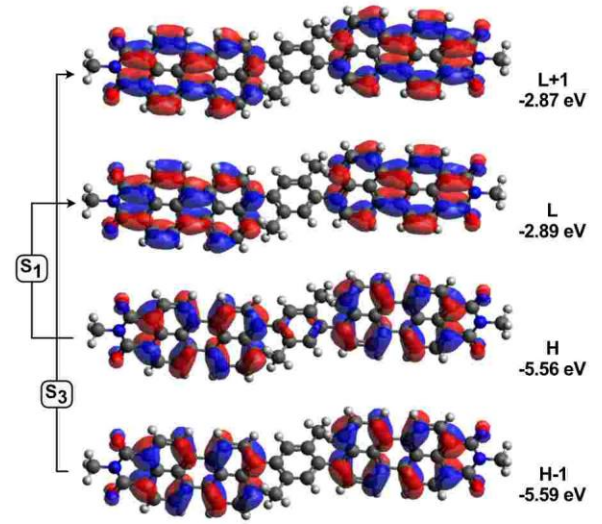

Fragment contributions in selected orbitals (%)

	A	B	C
L+1	14.0	85.2	0.8
L	13.8	84.8	1.4
H	7.1	90.3	2.5
H-1	7.3	91.7	1.0

B)

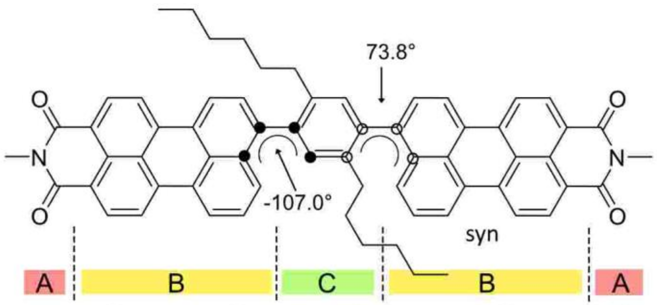

Excited state	eV	nm	Oscillator strength	Orbitals	Coefficients	Contribution of configuration (%)
S1	2.3721	522.67	0.7687	H -> L	0.71	99.4
S3	2.4638	503.21	0.7205	H-1 -> L-1	0.71	99.5

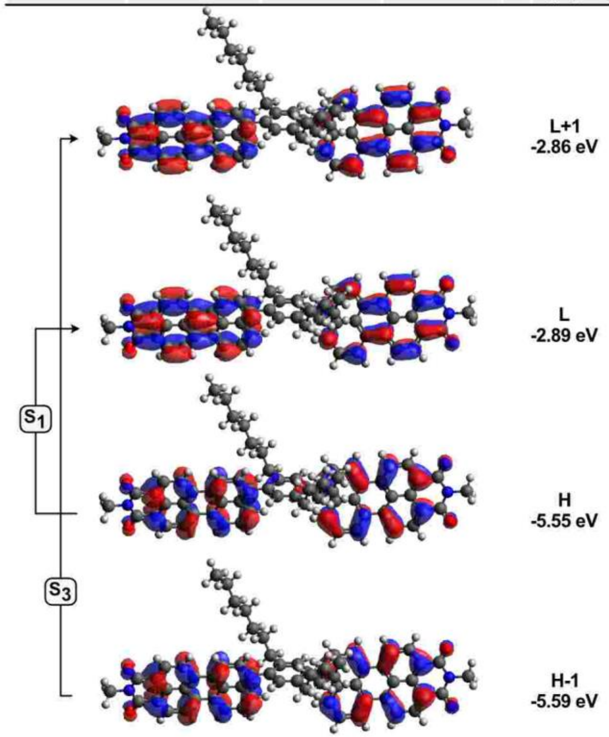

Fragment contributions in selected orbitals (%)

	A	B	C
L+1	14.1	85.2	0.7
L	13.8	85.1	1.1
H	7.1	90.1	2.6
H-1	7.3	91.6	0.9

Figure S7: Computation data of P-MePh-P (**7e**) and P-HexPh-P (**7f**).

A)

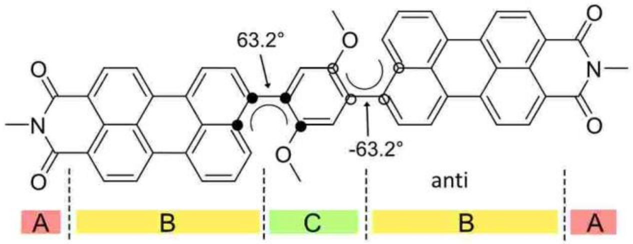

Excited state	eV	nm	Oscillator strength	Orbitals	Coefficients	Contribution of configuration (%)
S1	2.2779	544.29	0.8583	H-2 -> L	-0.14	3.9
				H -> L	0.69	95.7
S3	2.4650	502.97	0.6263	H-1 -> L-1	0.71	99.4

Fragment contributions in selected orbitals (%)

	A	B	C
L+1	14.3	84.7	1.0
L	13.7	83.8	2.5
H	6.5	82.9	10.4
H-1	7.3	91.5	1.2
H-2	0.9	13.3	82.8

Natural transition orbitals for excited state S_1

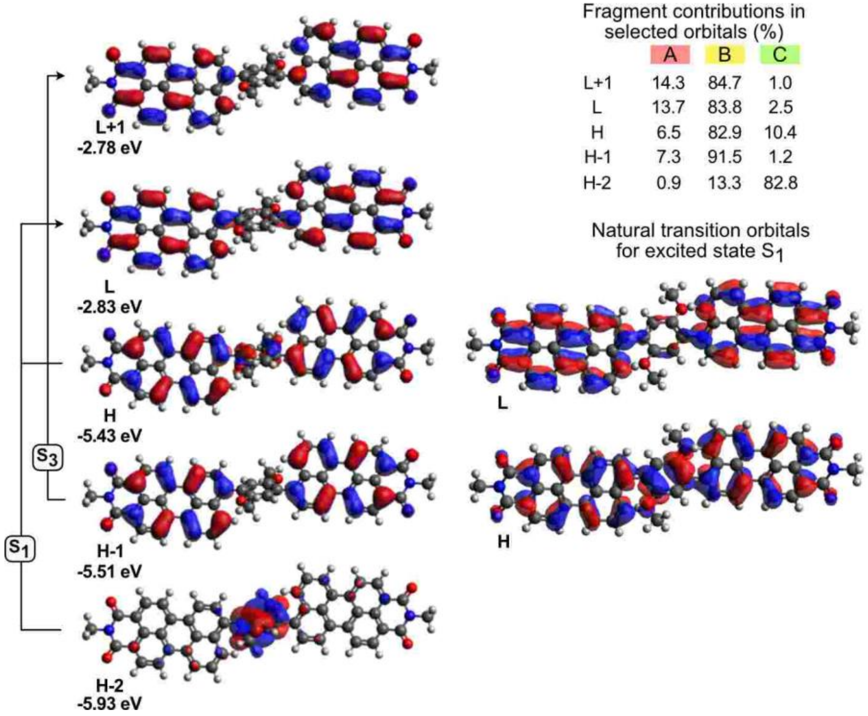

B)

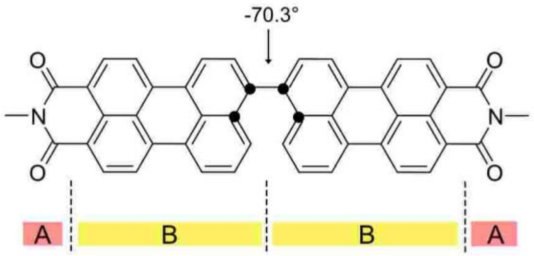

Excited state	eV	nm	Oscillator strength	Orbitals	Coefficients	Contribution of configuration (%)
S1	2.2465	551.90	0.5367	H-1 -> L+1	0.16	4.9
				H -> L	0.69	94.6
S3	2.4585	504.31	0.9245	H-1 -> L+1	0.69	94.7
				H -> L	-0.16	5.0

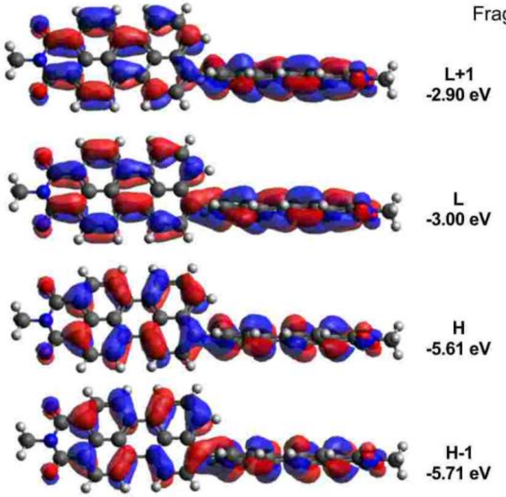

Fragment contributions in selected orbitals (%)

	A	B
L+1	14.1	85.9
L	13.4	86.6
H	7.2	92.7
H-1	7.3	92.7

Figure S8: Computation data of P-DeOPh-P (**7g**) and P-P (**6**).

Excited state	eV	nm	Oscillator strength	Orbitals	Coefficients	Contribution of configuration (%)
S4	2.4228	511.73	0.2342	H-2 -> L	0.52	54.1
				H-1 -> L+1	0.20	8.2
				H -> L	-0.25	12.8
				H -> L+2	0.30	22.5
S5	2.4233	511.63	0.2331	H-2 -> L+1	0.55	60.9
				H-1 -> L	0.21	8.6
				H-1 -> L+2	0.34	22.8
				H -> L+1	0.17	5.8
S7	2.4802	499.90	0.9516	H-2 -> L	-0.26	13.3
				H-1 -> L+1	-0.14	4.1
				H -> L	0.16	5.4
				H -> L+2	0.61	75.5
S8	2.4805	499.85	0.9565	H-2 -> L+1	-0.28	15.9
				H-1 -> L	-0.15	4.2
				H-1 -> L+2	0.62	75.7
				H -> L+1	-0.13	3.1

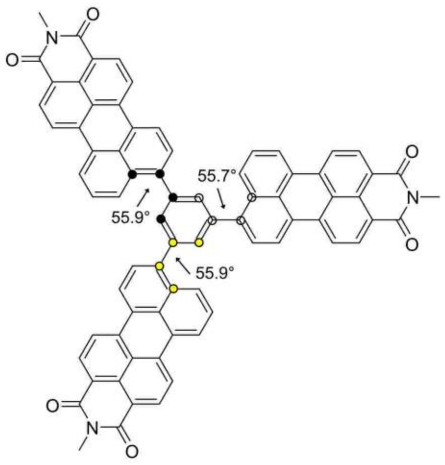

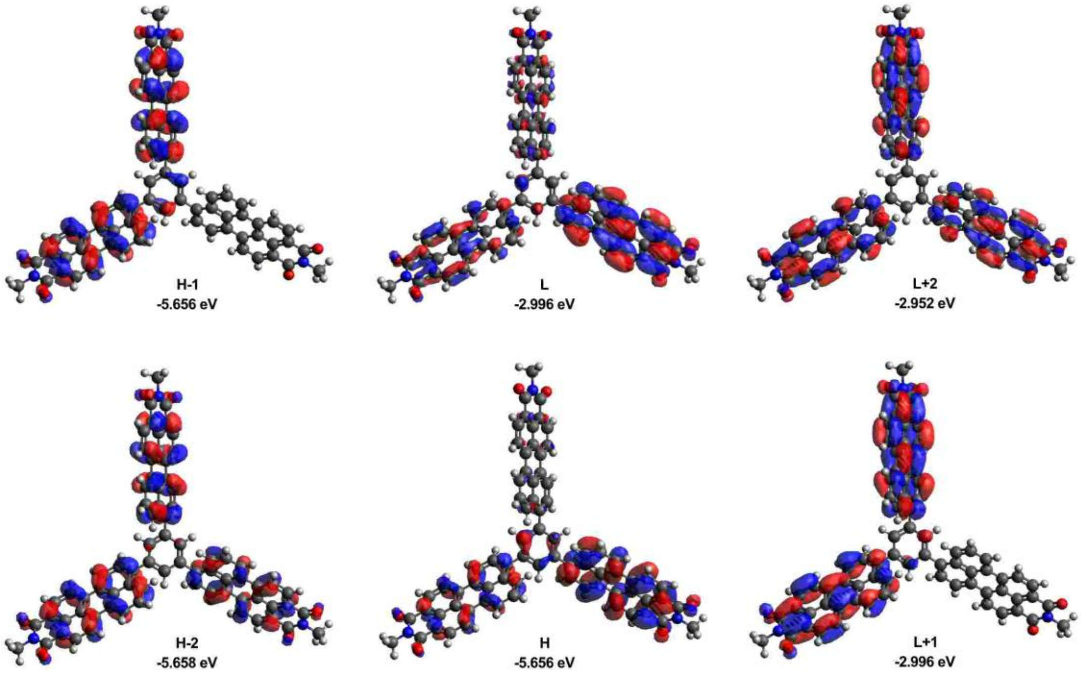

Figure S9: Computation data of PMI_3Ph (**7d**); all results in B3LYP/6-31G(d,p) level of theory. Geometry optimization could not converge using gaussian09 standard (Fine) integration grid thus UltraFine grid was used instead. The converged geometry was verified by frequency calculations and then used for single point calculation with standard gaussian09 parameters.

S3. Crystal structure data

Table S2 Crystal data and structure refinement for **1** and **4**.

CCDC	2144711	2144712
Substance Identifier	**1** (**P-H**)	**4** (**P-Ph**)
Empirical formula	$C_{34}H_{27}NO_2$	$C_{40}H_{31}NO_2 \cdot CHCl_3$
Formula weight	481.56	677.02
Temperature/K	99.99	99.99
Crystal system	orthorhombic	triclinic
Space group	$Pca2_1$	P-1
a/Å	19.1292(17)	17.2533(12)
b/Å	8.6116(8)	20.0636(16)
c/Å	30.009(2)	21.2621(16)
α/°	90	76.528(5)
β/°	90	89.918(4)
γ/°	90	65.900(4)
Volume/Å^3	4943.5(7)	6497.2(9)
Z	8	8
ρ_{calc}g/cm^3	1.294	1.384
μ/mm^{-1}	0.080	0.321
F(000)	2032.0	2816.0
Crystal size/mm^3	0.22 × 0.18 × 0.15	0.24 × 0.21 × 0.09
Radiation	MoKα (λ = 0.71073)	MoKα (λ = 0.71073)
2Θ range for data collection/°	4.258 to 54	1.98 to 51
Index ranges	-24 ≤ h ≤ 24, -11 ≤ k ≤ 11, -38 ≤ l ≤ 38	-20 ≤ h ≤ 20, -24 ≤ k ≤ 24, -25 ≤ l ≤ 25
Reflections collected	82181	365141
Independent reflections	10713 [R_{int} = 0.0718, R_{sigma} = 0.0732]	24188 [R_{int} = 0.1408, R_{sigma} = 0.0622]
Data/restraints/parameters	10713/1/675	24188/1044/1634
Goodness-of-fit on F^2	1.023	1.117
Final R indexes [I>=2σ (I)]	R_1 = 0.0481, wR_2 = 0.1096	R_1 = 0.1305, wR_2 = 0.3067
Final R indexes [all data]	R_1 = 0.0931, wR_2 = 0.1274	R_1 = 0.1683, wR_2 = 0.3428
Largest diff. peak/hole / e Å^{-3}	0.22/-0.26	1.21/-1.23

P-H (**1**) crystallizes in the orthorhombic point group $Pca2_1$ with two independent molecules in the asymmetric unit. The perylene fragments adopt an almost planar conformation in the solid state, while the 2,6-$^{i}Pr_2$-C_6H_3 substituents at nitrogen adopt dihedral angles of 83.8° and 90.0°, respectively. The two independent unit form dimers via π-stacking where the perylene units are separated by 3.41 Å.

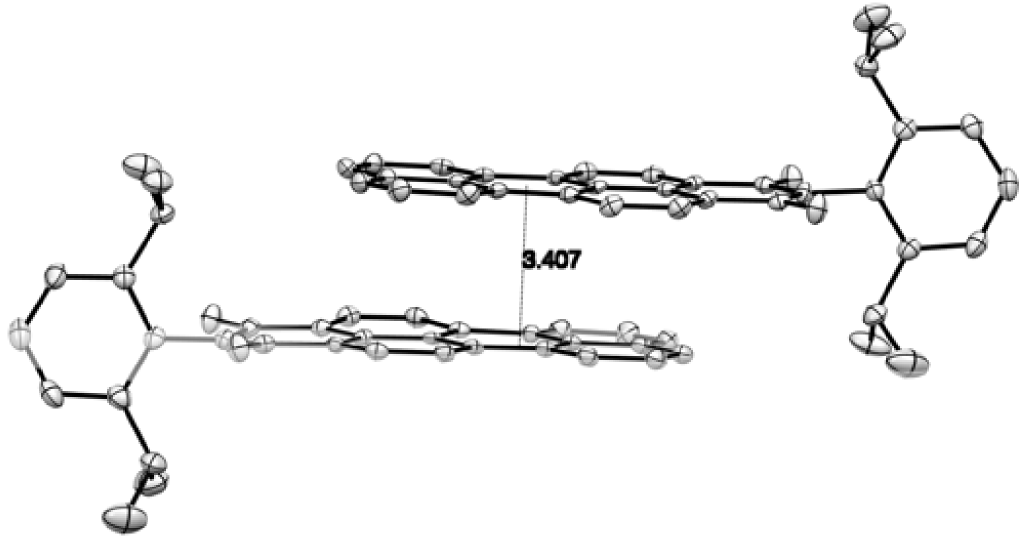

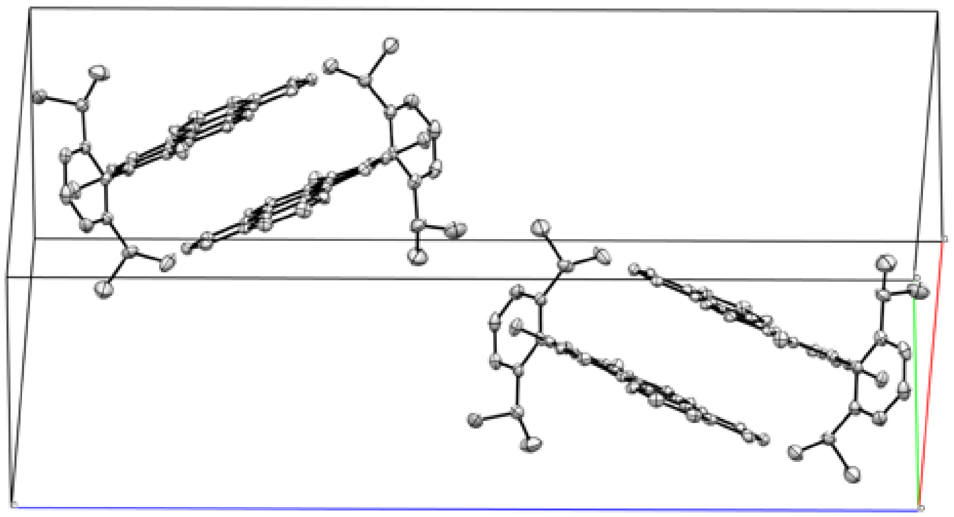

Figure S10: Single-crystal X-ray structure of perylene monoimide P-H (**1**).

P-Ph (**4**) crystallizes in the triclinic space group P-1 with four independent molecules per asymmetric unit and additional chloroform solvate molecules. In **4**, the dimers are separated by 3.49 and 3.51 Å, respectively. Similar to **1**, the dihedral angles of the 2,6-$^{i}Pr_2$-C_6H_3 substituents at nitrogen are oriented almost perpendicular to the planar perylene core and adopt dihedral angles of 79.38° to 89.52°. In contrast, the sterically less demanding phenyl groups on the perylene core are rotated by 63°. In agreement with results from GIWAXS measurements (see the next page), the quality of crystals of **4** grown from chloroform is inferior to **1** hampering final R values for the refinement of **4**.

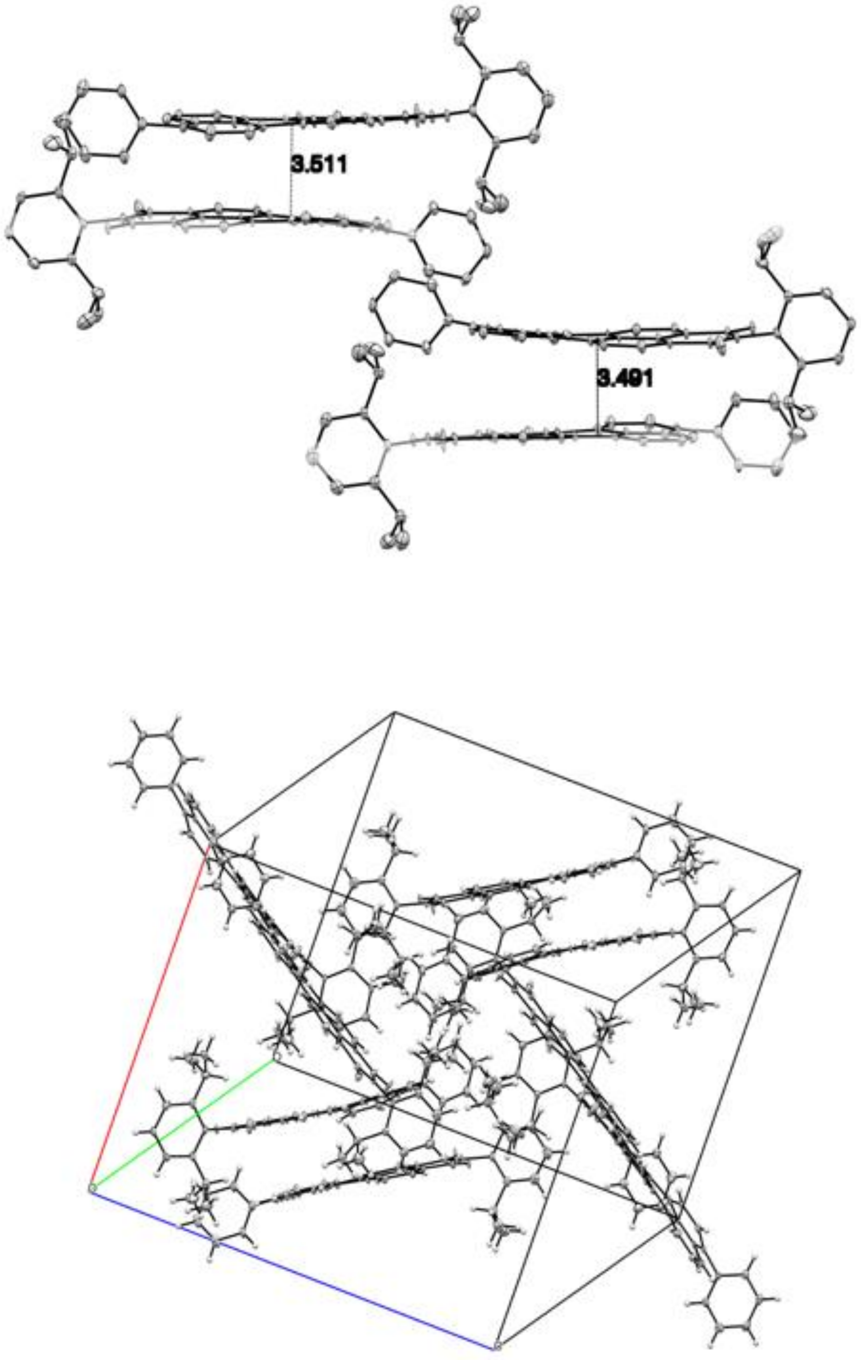

Figure S11: Single-crystal X-ray structure of perylene monoimide P-Ph (**4**).

S4. GIWAXS

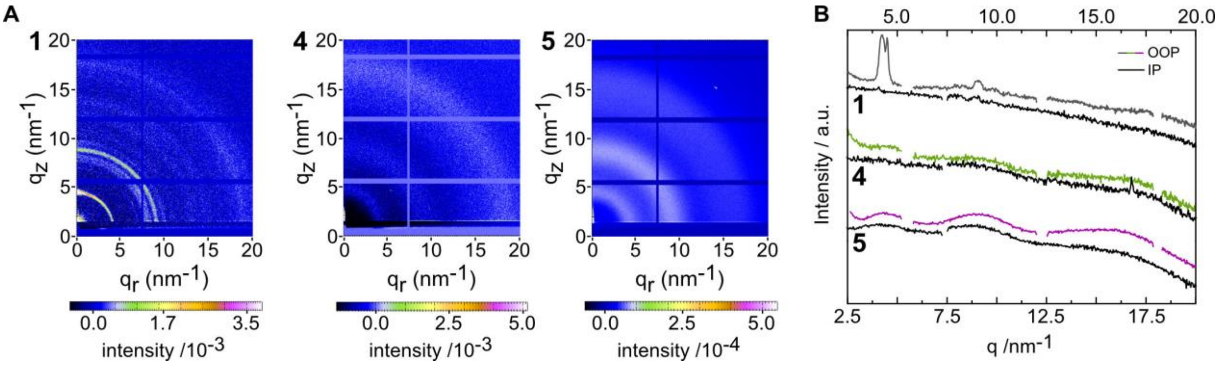

Figure S12: A - 2D-GIWAXS pattern and B - 1D line-cuts cuts in the in plane (IP) and out-of-plane directions (OOP) of compounds **1**, **4** and **5**.

S5. Optical, thermal and electrochemical data

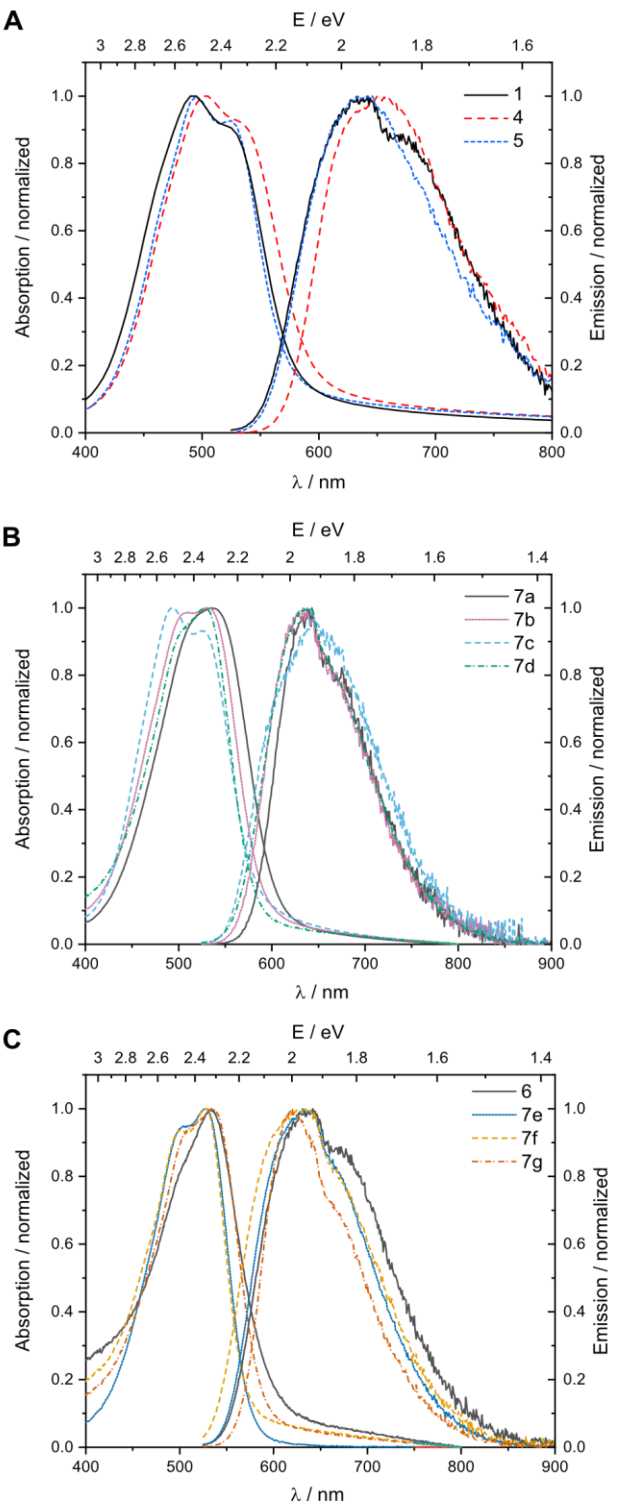

Figure S13: Normalized film absorption and emission spectra of compounds **1**,**4-5** (A), **6**, **7a-g** (B,C).

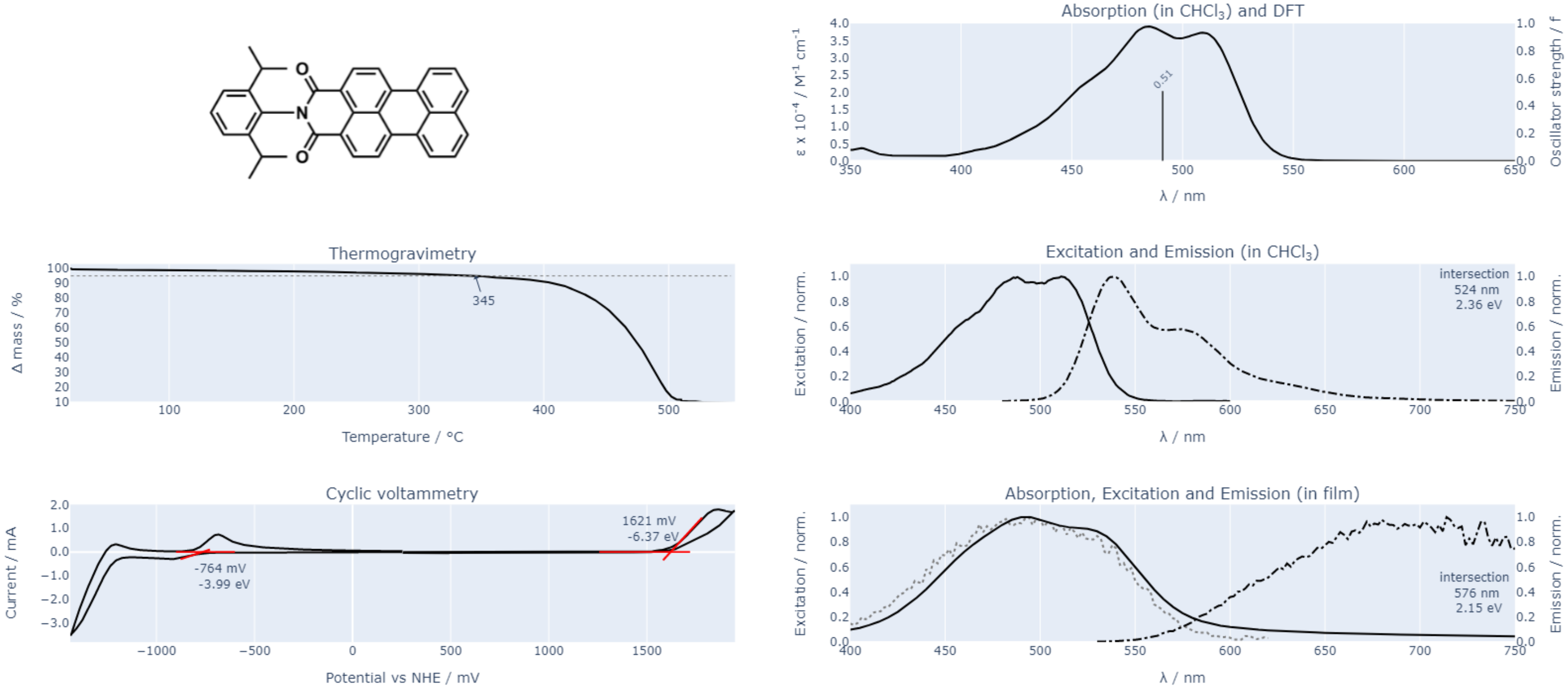

Figure S14: Summary of experimental data for compound **1**.

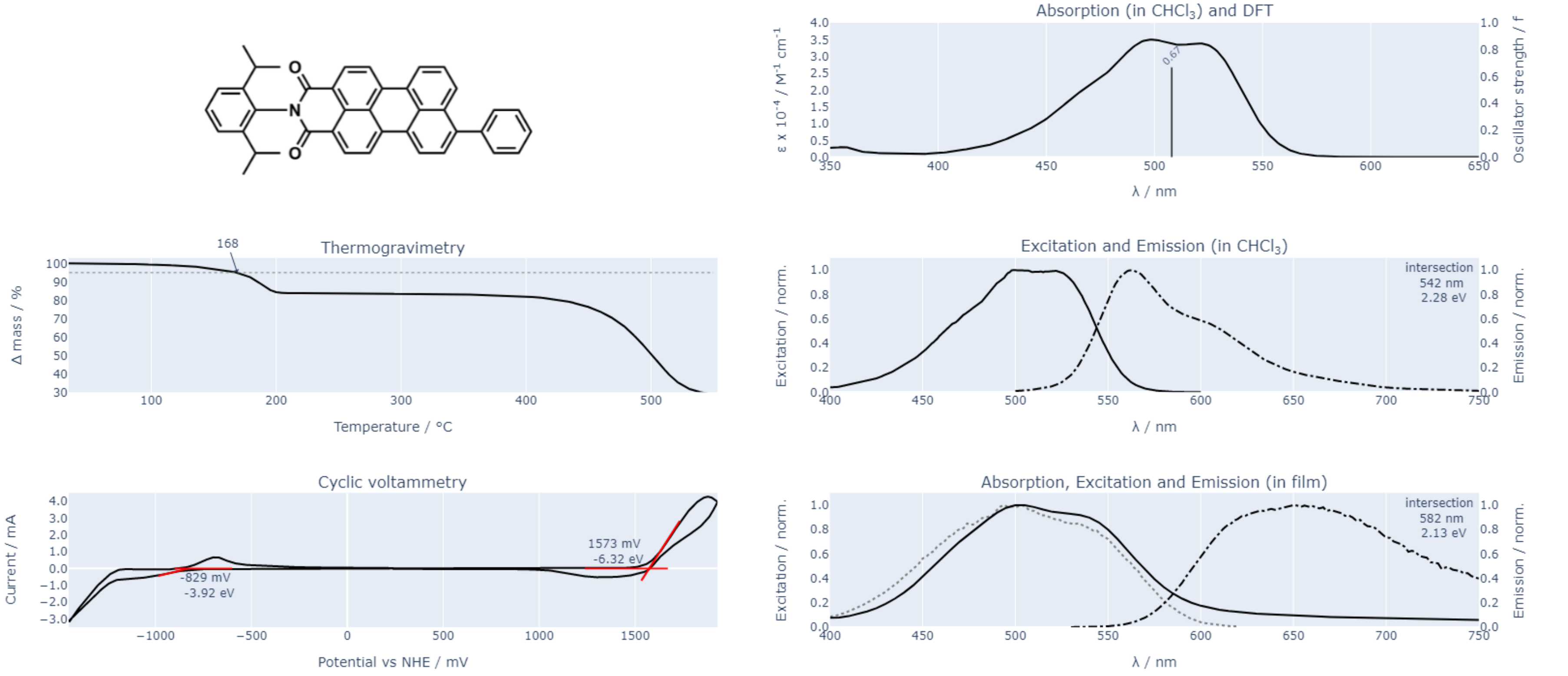

Figure S15: Summary of experimental data for compound **4**.

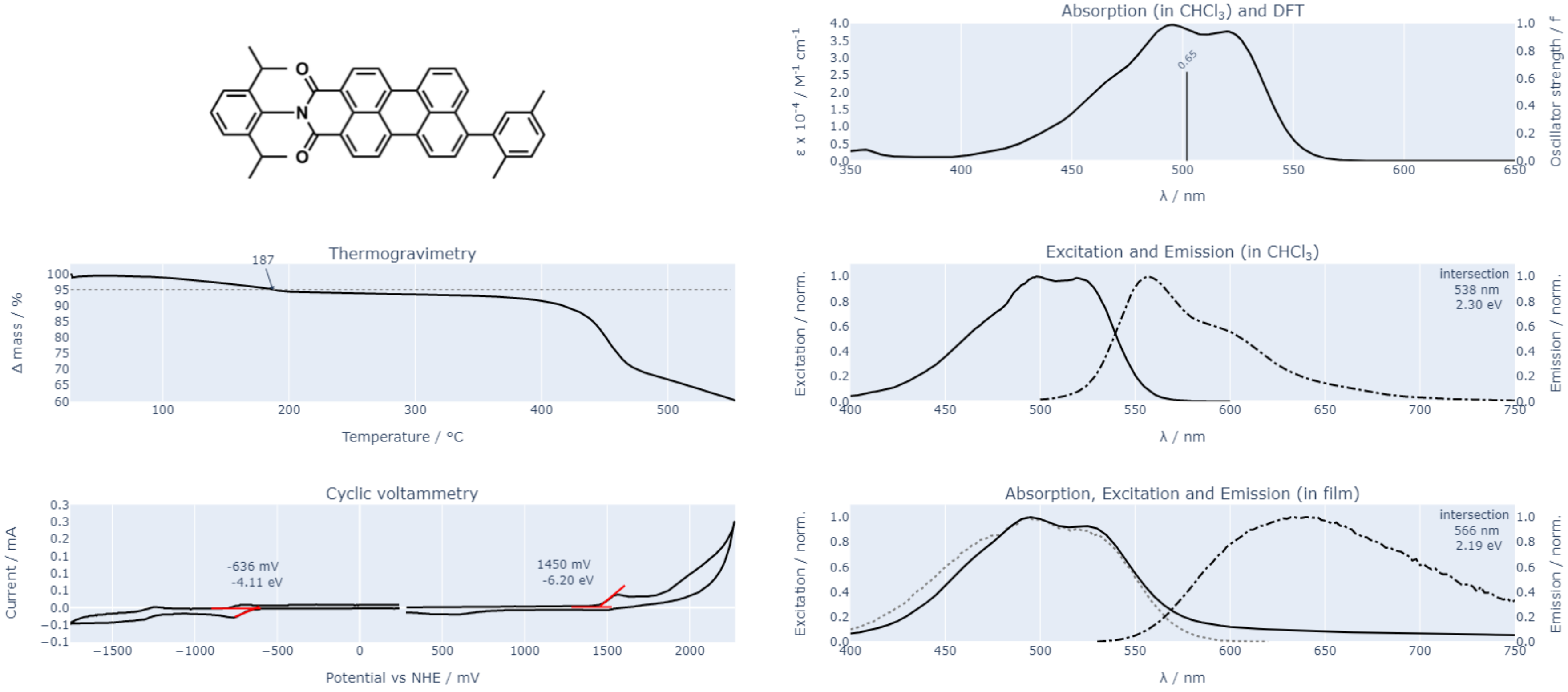

Figure S16: Summary of experimental data for compound **5**. CV was measured in solution.

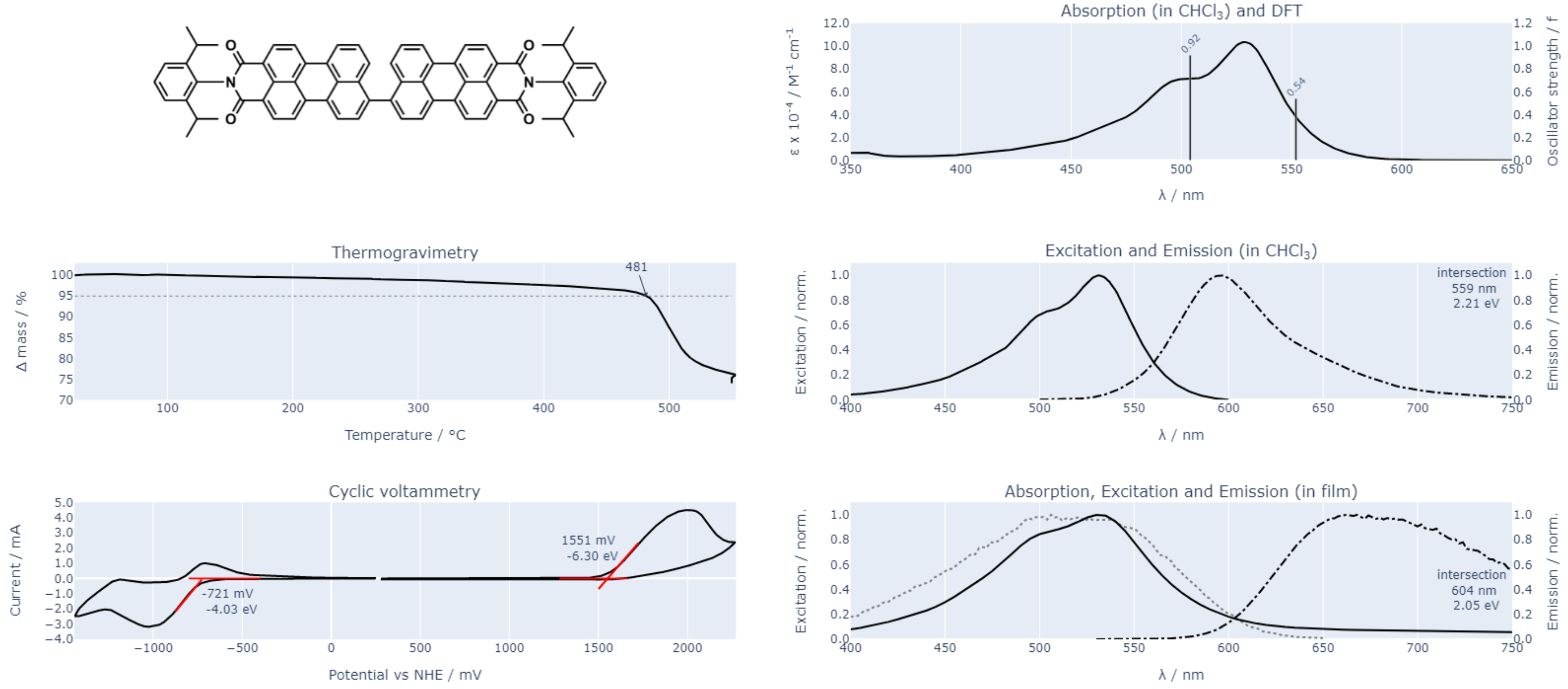

Figure S17: Summary of experimental data for compound **6**.

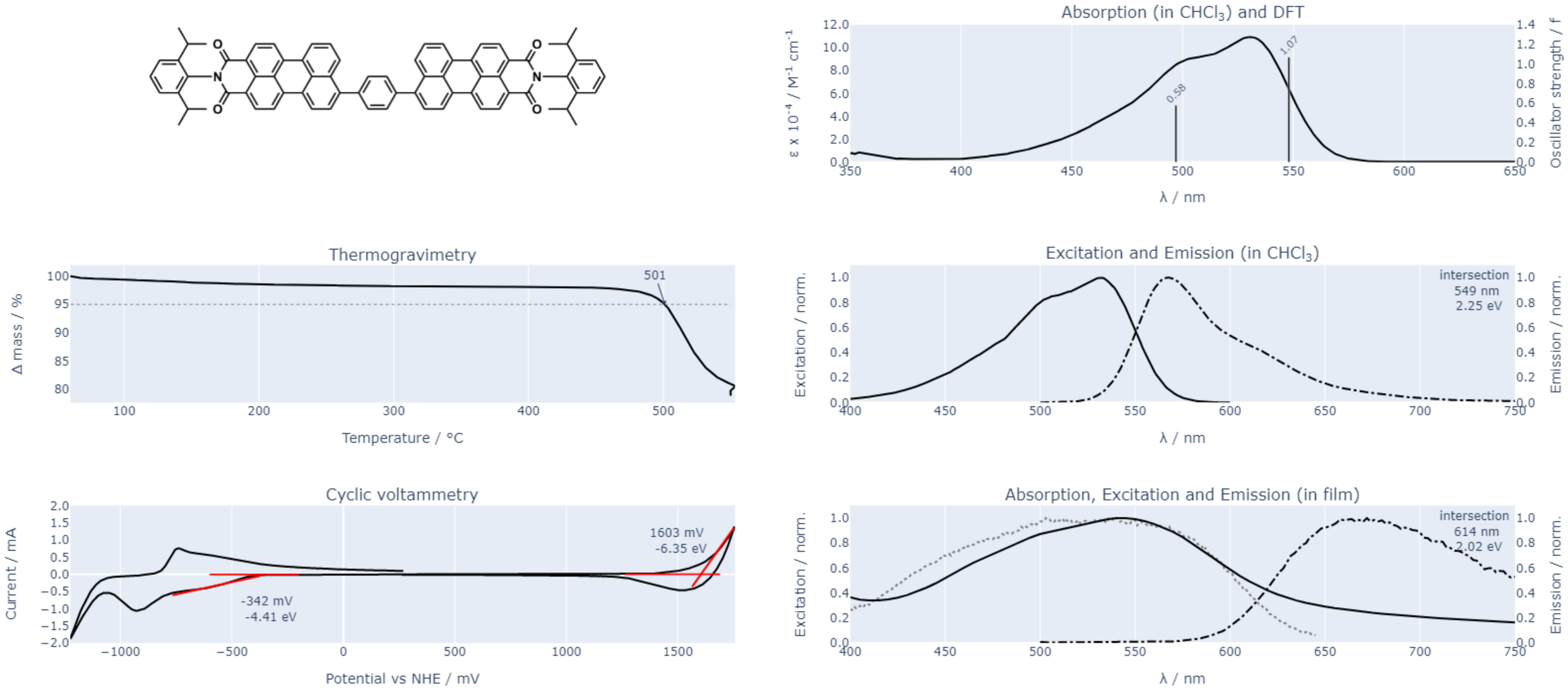

Figure S18: Summary of experimental data for compound **7a**.

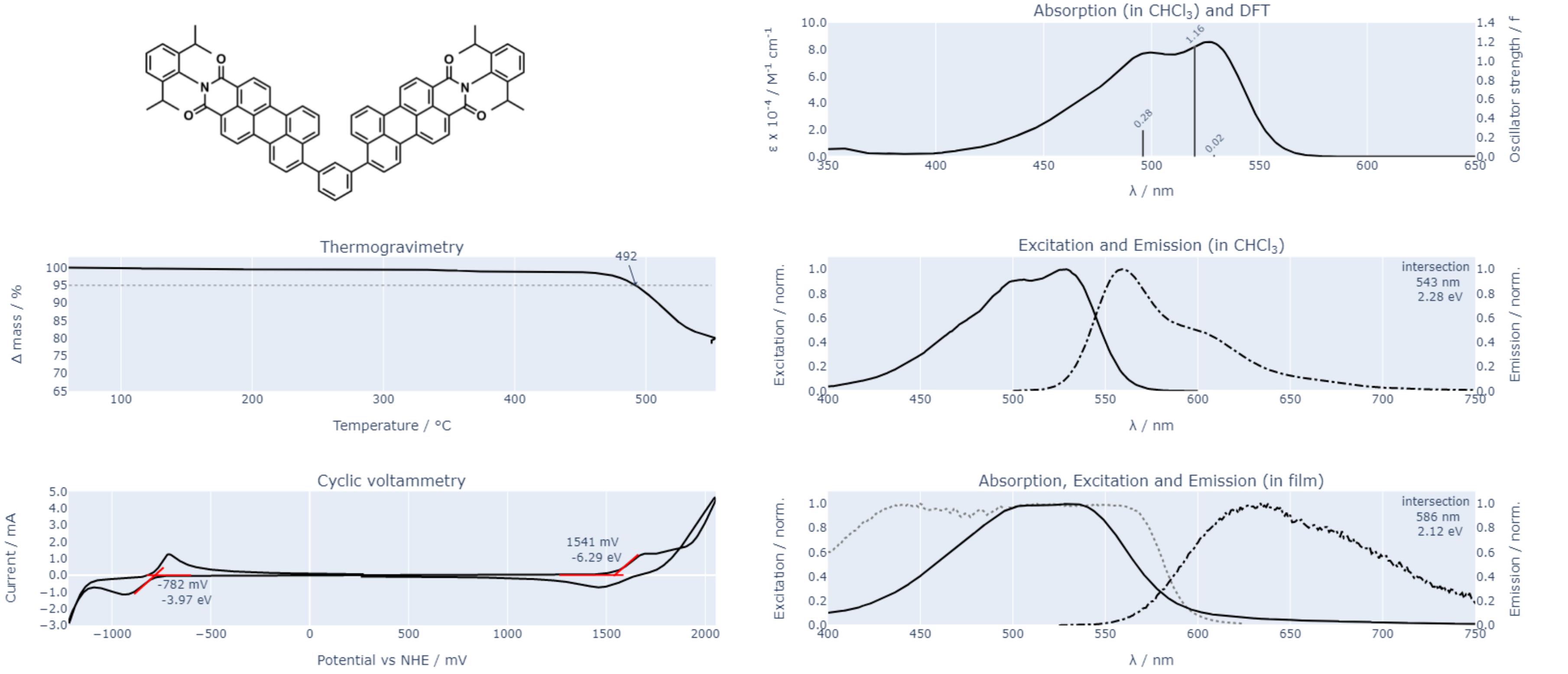

Figure S19: Summary of experimental data for compound **7b**.

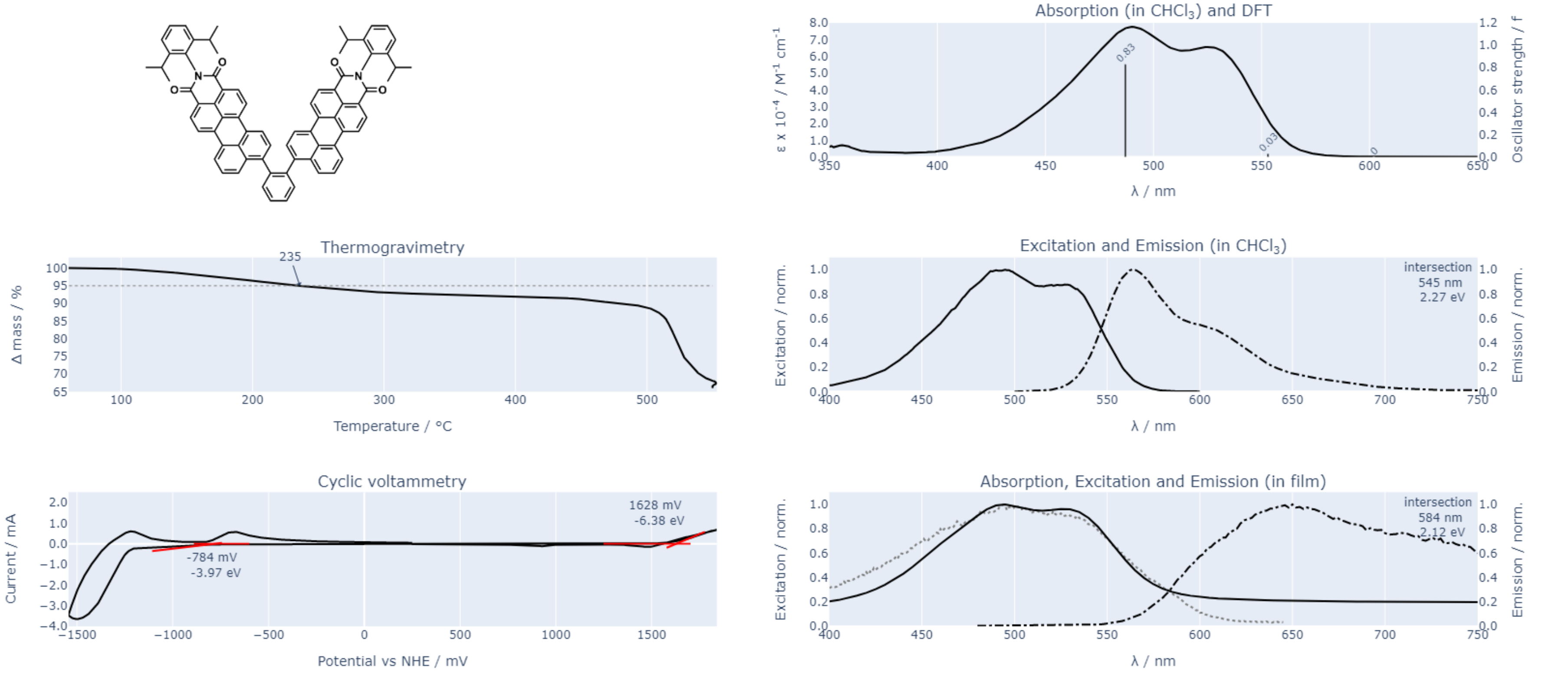

Figure S20: Summary of experimental data for compound **7c**.

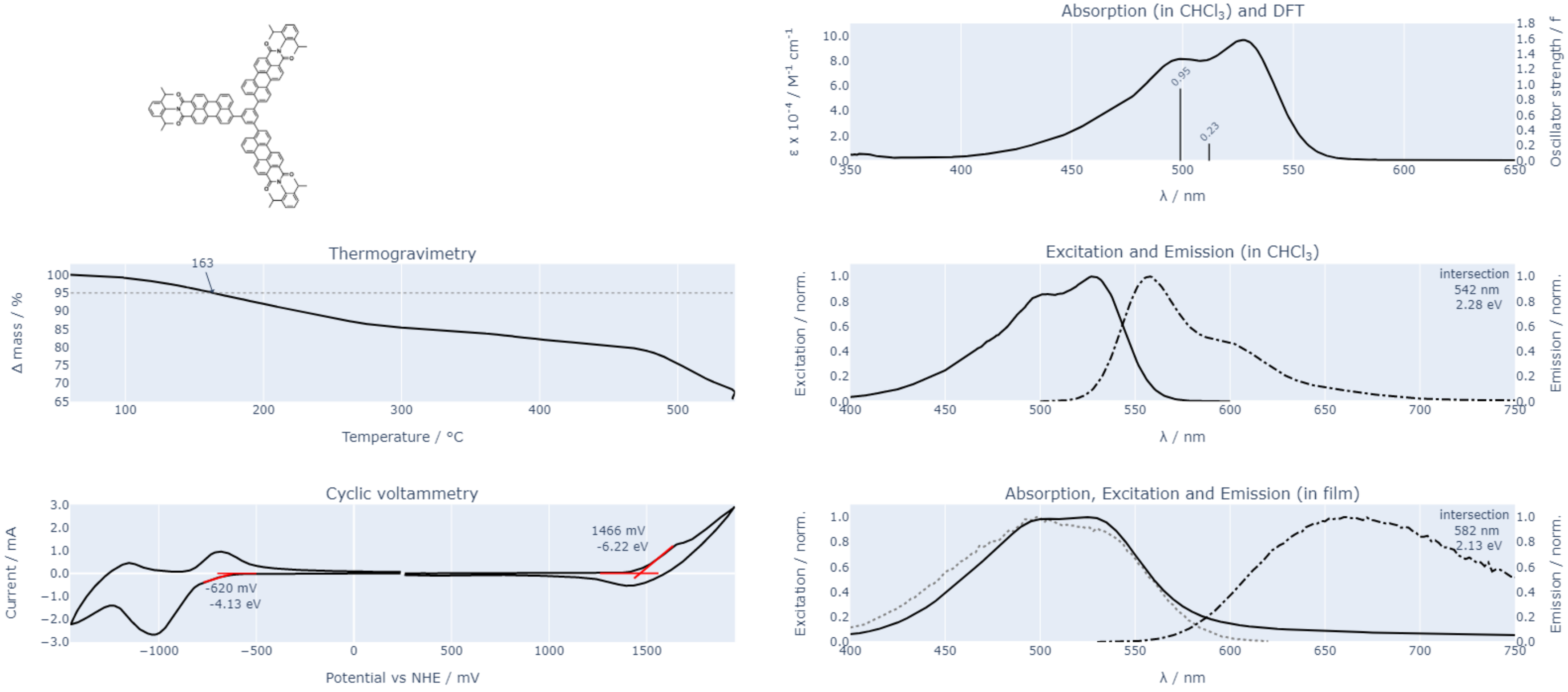

Figure S21: Summary of experimental data for compound **7d**.

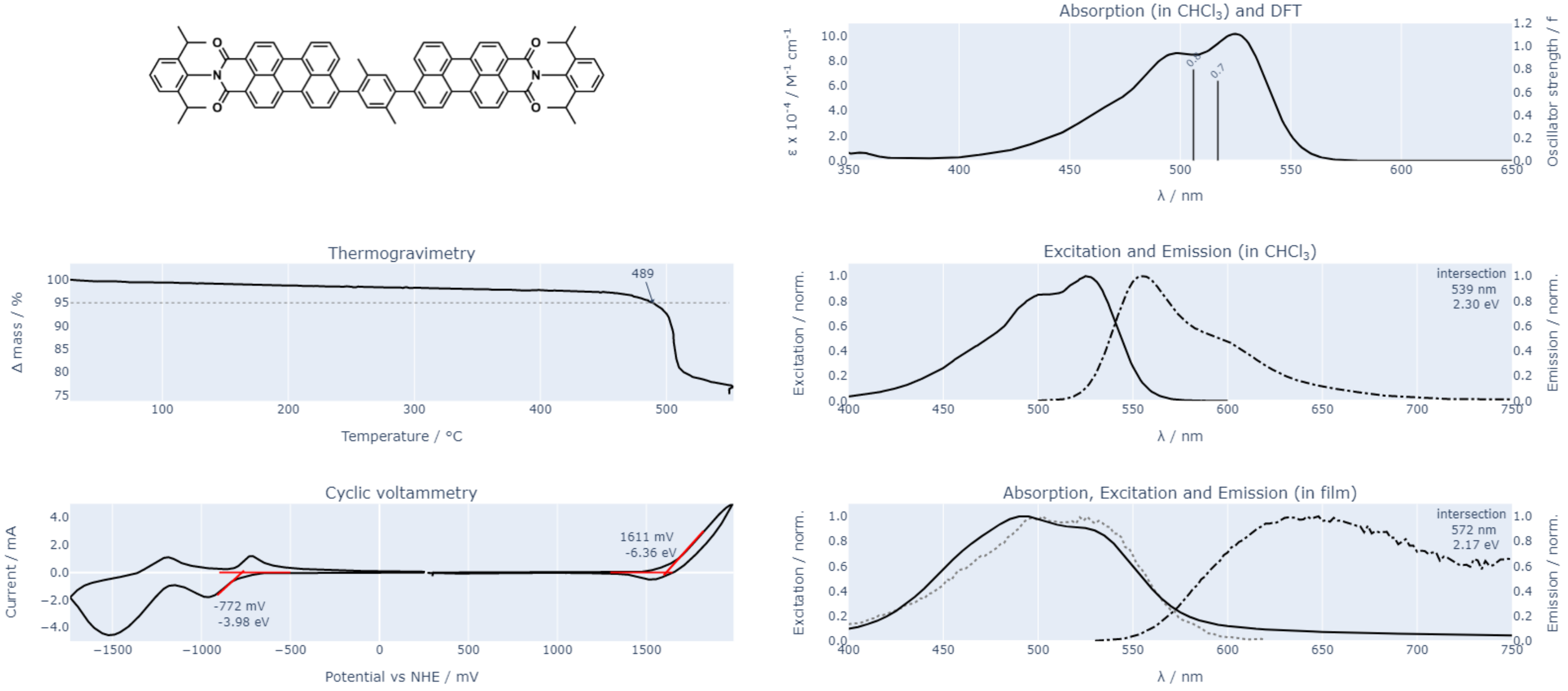

Figure S22: Summary of experimental data for compound **7e**.

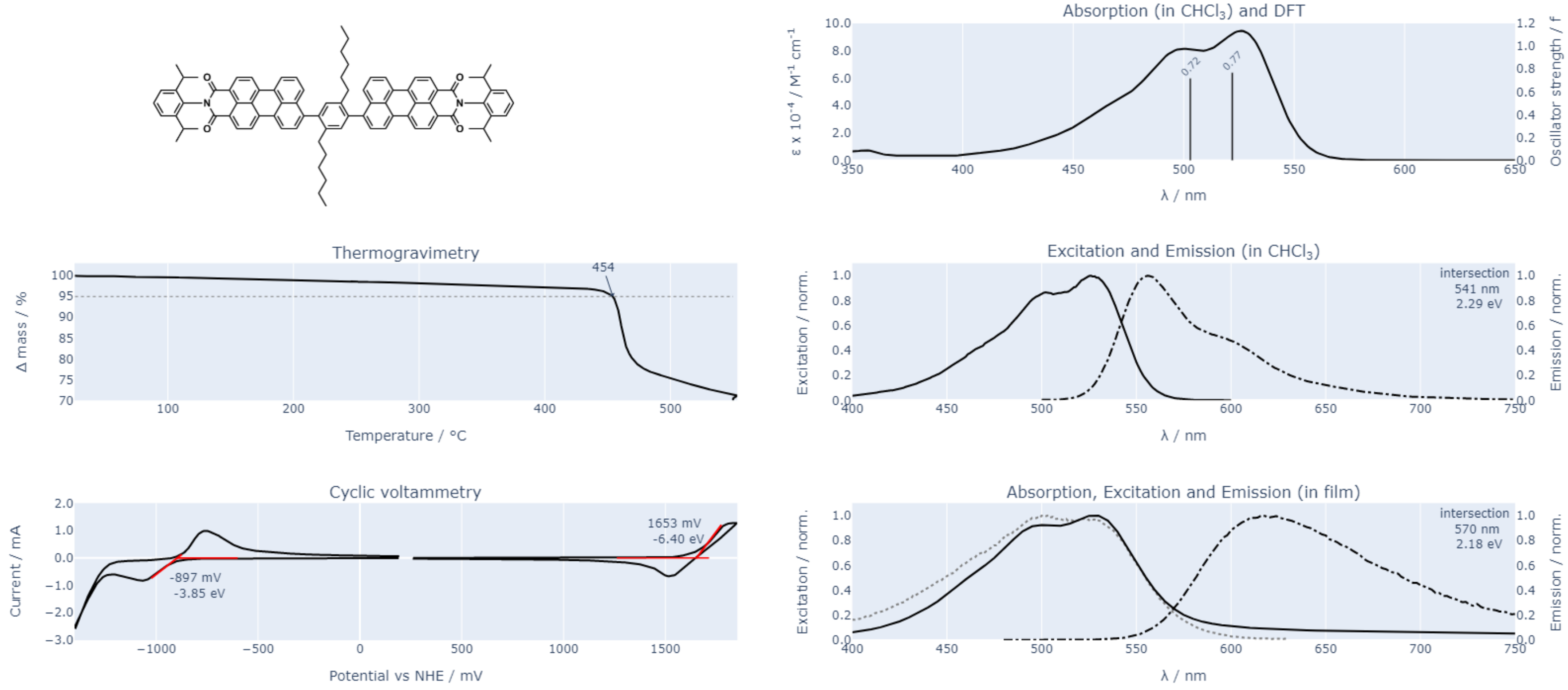

Figure S23: Summary of experimental data for compound **7f**.

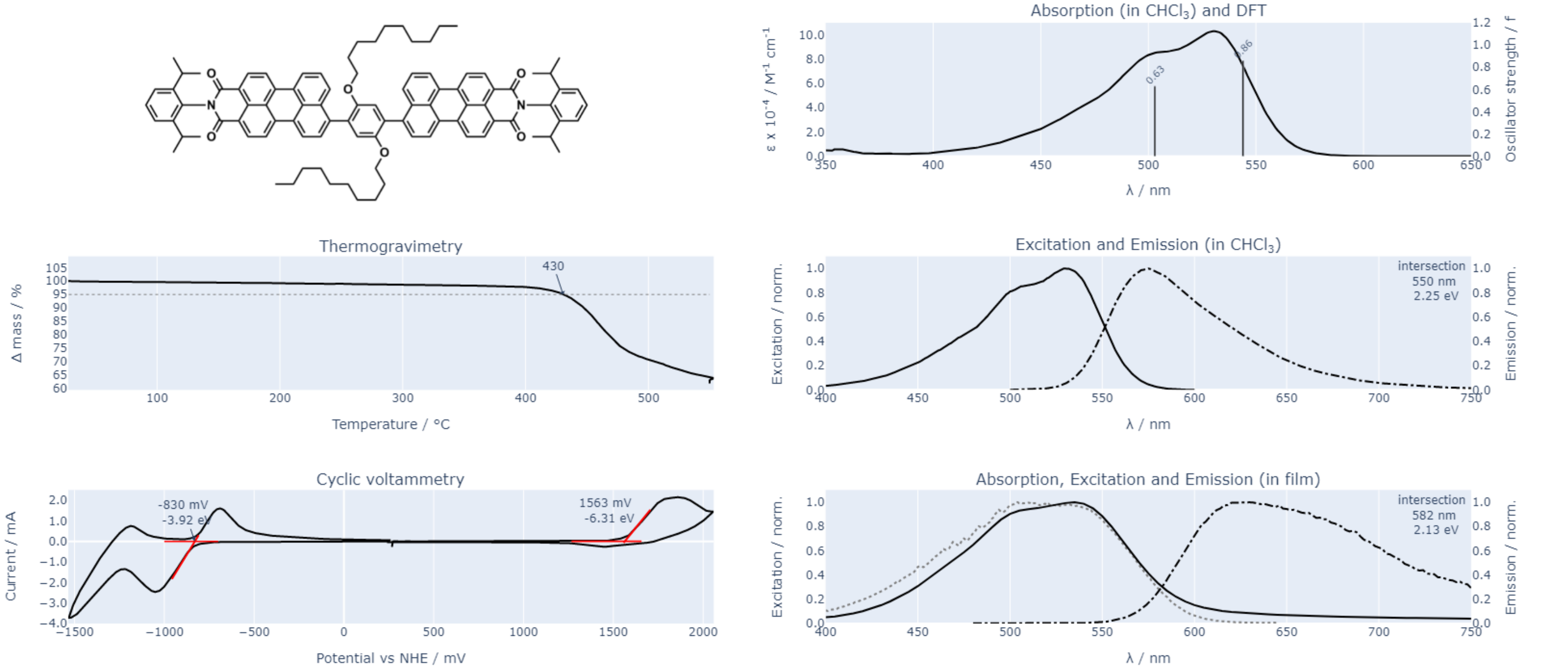

Figure S24: Summary of experimental data for compound **7g**.

Electrochemical properties

Table S3: Oxidation and reduction potentials, HOMO and LUMO energy levels.

Compound	CV[a]					DFT[b]		
	E_{red} (V)	E_{ox}(V)	HOMO (eV)	LUMO (eV)	Gap (eV)	HOMO (eV)	LUMO (eV)	Gap(eV)
**1** (P-H)	-0.76	1.62	-6.37	-3.99	2.38	-5.52	-2.79	2.73
**6** (P-P)	-0.72	1.55	-6.30	-4.03	2.27	-5.61	-3.00	2.60
**7a** (P-pPh-P)	-0.34	1.60	-6.35	-4.41	1.94	-5.50	-2.94	2.56
**7b** (P-mPh-P)	-0.78	1.54	-6.29	-3.97	2.32	-5.55	-2.90	2.65
**7c** (P-oPh-P)	-0.78	1.63	-6.38	-3.97	2.41	-5.44	-2.81	2.63
**7d** (P3Ph)	-0.62	1.47	-6.22	-4.13	2.09	-5.66	-3.00	2.66
**7e** (P-MePh-P)	-0.77	1.61	-6.36	-3.98	2.38	-5.57	-2.88	2.68
**7f** (P-HexPh-P)	-0.90	1.65	-6.40	-3.85	2.55	-5.55	-2.89	2.66
**7g** (P-DeOPh-P)	-0.83	1.56	-6.31	-3.92	2.39	-5.43	-2.84	2.60

[a] film measurements; [b] DFT B3LYP-GD3/6-31G(d,p).

S6. Synthesis procedures

Perylene monoimide building block

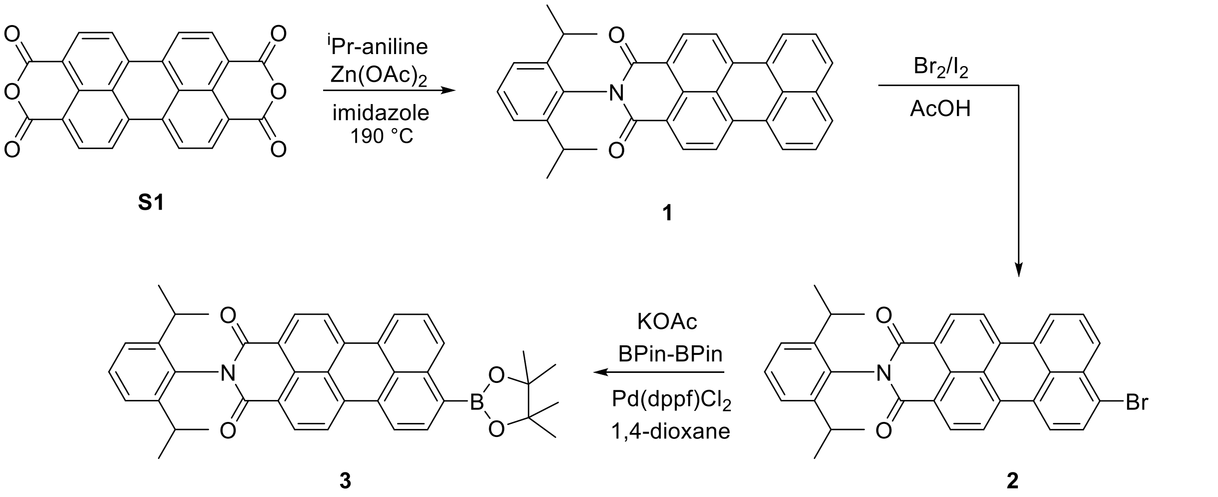

Perylene monoimide (**P-H**, **1**)

In a beaker 16.0 g (40.8 mmol, 1 eq.) of perylenetetracarboxylic dianhydride (**S1**) was mixed with imidazole (80 g, 1.18 mol, 29 eq.) and $Zn(OAc)_2$ (5.99 g, 27.3 mmol, 0.67 eq.) before being transferred to an autoclave vessel. The mixture was then suspended in water (20 mL) followed by addition of 2,6-diisopropylaniline (4.1 mL, 3.82 g, 21.6 mmol, 0.53 eq.). The vessel was sealed and stirred at 190°C for 24 h. After cooling to room temperature it was diluted with water (ca. 200 mL), acidified until pH 2 using HCl (10%), filtered. The dark red residue was air dried. Once dry, it was grinded and transferred to a beaker in which also 250 mL of $CHCl_3$ was added. The suspension was sonicated for 15 min,

decanted via filter paper. The clear, dark orange solution was partially evaporated until precipitate started to appear on the sides of the flask. A large amount of MeOH (at least equal to that of leftover $CHCl_3$) was added which causes formation of a red solid on the solvent mixing surface; later both solvents were mixed mechanically to achieve quantitative precipitation of the product. Identical treatment to the crude product was repeated further two times. Dark solid left after third extraction step was further extracted using Soxhlet set up. Soxhlet extraction was not used initially because upon reaching too high concentrations, product precipitates together with side products as a dark solid, which is then difficult to redissolve in $CHCl_3$ for further purification. Using the precipitation with MeOH the product is be obtained as red solid (3.6 g, 18%). It is pure enough to be used for further reactions without additional purification. Alternatively, a simple purification on SiO_2 with $CHCl_3$ as eluent can be performed. ^{1}H NMR (300 MHz, $CDCl_3$): δ 8.65 (d, 2H, *J* = 8.0 Hz), 8.49-8.40 (m, 4H), 7.91 (d, 2H, *J* = 8.0 Hz), 7.69-7.59 (m, 2H), 7.52-7.43 (m, 1H), 7.34 (d, 2H, *J* = 7.7 Hz), 2.77 (sept, 2H, *J* = 6.8 Hz), 1.18 (d, 12H, *J* = 6.8 Hz) ppm, see alsoFigure S25.

9-Bromo perylene monoimide (**P-Br, 2**)

Perylene monoimide (**1**) (10.00 g, 20.8 mmol, 1 eq.) was suspended in AcOH (250 mL) and stirred for 30 min before the addition of Br_2 (13.28 g, 83.2 mmol, 4 eq.) in a one portion. Mixture was stirred in a room temperature for 24 h (conversion followed by TLC). The starting material and product has similar retention factors; in order to achieve optimal TLC results solutions with low concentration should be used; Spotting a co-spot is advisable (starting material together with the reaction mixture); Toluene with acetone is suitable eluent (if only toluene is used, the TLC has to be eluted at least 3 times; upon increased acetone content (up to 10%) less elution times are needed). Upon completion, extra bromine was partially evaporated by a gentle stream of air (in fume hood!) for 30 min. Then MeOH (250 mL) was added and the mixture stirred for 30 min before pouring into 1 L of water. The formed bright red precipitate was filtered and washed with a large amount of water (until the filtrate is neutral). Initially also diluted $NaHCO_3$ solution can be used. The bright red solid was air dried (with a regular mixing and grinding). 11 g of product (95%) was obtained. This solid is pure enough to be used for further reactions without additional purification. Alternatively, a simple purification on SiO_2 with $CHCl_3$ as eluent can be performed. ^{1}H NMR (300 MHz, $CDCl_3$): δ 8.71-8.61 (m, 2H), 8.53-8.44 (m, 2H), 8.42 (d, 1H, *J* = 8.1 Hz), 8.32 (d, 1H, *J* = 8.4 Hz), 8.25 (d, 1H, *J* = 8.4 Hz), 7.92 (d, 1H, *J* = 8.2 Hz), 7.78-7.69 (m, 1H), 7.53-7.44 (m, 1H), 7.34 (d, 2H, *J* = 7.7 Hz), 2.77 (sept, 2H, *J* = 6.8 Hz), 1.18 (d, 12H, *J* = 6.8 Hz) ppm, see also Figure S26.

Perylene monoimide 9-boronic acid pinacol ester (**P-BPin, 3**)

In a round bottom flask Pd(dppf)Cl_2 (522 mg, mol%) and dry KOAc (2.106 g, 21.4 mmol, 3 eq.) was weighted (in a glow box), to this the starting material **P-Br** (**2**) (4.000 g, 7.14 mmol, 1 eq.) and bis(pinacolato)diboron (2.177 g, 8.57 mmol, 1.2 eq.) was added (outside of glow box, under nitrogen flow). After addition of dry dioxane (70 mL) the mixture was stirred at 80 °C overnight (under nitrogen pressure). TLC control showed full conversion. A red precipitate was formed once the reaction mixture was cooled to room temperature. This was filtrated and washed with 3 x 50 mL of MeOH. The dark red solid was air dried to give 3.4 g (78%) of product in a quality which is good enough to be used in further reactions (e.g. Suzuki coupling). Further purification can be performed by recrystallization from toluene if necessary (e.g. to remove small amount of solid which is insoluble in toluene). Alternatively, purification can be done via SiO_2 with $CHCl_3$ or EtOAc/Cyclohexane as eluent, however it's worth noting that the product is slightly instable on SiO_2. ^{1}H NMR (300 MHz, $CDCl_3$): δ 8.87 (d, 1H, *J* = 8.4 Hz), 8.69-

8.60 (m, 2H), 8.51-8.39 (m, 4H), 8.87 (d, 1H, *J* = 7.3 Hz), 7.72-7.62 (m, 1H), 7.52-7.44 (m, 1H), 7.34 (d, 2H, *J* = 7.7 Hz), 2.77 (sept, 2H, *J* = 6.8 Hz), 1.47 (s, 12H), 1.18 (d, 12H, *J* = 6.8 Hz) ppm, see also Figure S27.

Suzuki couplings

Synthesis of **4** (**P-Ph**)

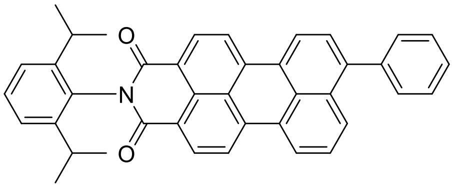

Chemical Formula: $C_{40}H_{31}NO_2$
Molecular Weight: 558

In a round bottom flask to **P-Br** (**1**) (417 mg, 0.74 mmol, 1 eq) and phenylboronic acid (109 mg, 0.89 mmol, 1.2 eq.) was suspended in a mixture of 50 mL toluene and 4 mL EtOH. To this mixture KF (1 M, 3 mL) was added, and the solution was degassed by a flow of nitrogen for 20 min. Then $Pd(PPh_3)_4$ (86 mg, 10 mol%) was added and the mixture refluxed for 16 h (under nitrogen). The solution was cooled to room temperature, extracted with toluene, washed with deionized water and brine, dried over Na_2SO_4, and concentrated. Purification was done by silica gel column (toluene/acetone = 99/1) followed by recrystallization from ethyl acetate and another silica gel column (cyclohexane/ethyl acetate = 9/1). Compound **3** (142 mg, 34%) was obtained as a red powder. ^{1}H NMR (500 MHz, $CDCl_3$): δ 8.70-8.65 (m, 2H), 8.55-8.47 (m, 4H), 8.02 (d, 1H, *J* = 8.4 Hz), 7.64-7.59 (m, 2H), 7.58-7.54 (m, 4H), 7.53-7.45 (m, 2H), 7.34 (d, 2H, *J* = 7.9 Hz), 2.78 (sept, 2H, *J* = 6.8 Hz), 1.18 (d, 12H, *J* = 6.8 Hz) ppm. ^{13}C NMR (75 MHz, $CDCl_3$): δ 164.2, 145.9, 143.6, 140.0, 137.9, 137.8, 132.8, 132.3, 132.2, 131.2, 130.7, 130.1, 129.6, 129.5, 128.7, 128.6, 128.5, 128.4, 128.2, 127.2, 124.2, 124.1, 123.7, 121.1, 120.5, 120.2, 29.3, 24.2 ppm, see also Figure S29. HRMS (MALDI) m/z calcd for $C_{40}H_{31}NO_2$: 557.2355 $[M]^+$; found 557.2344.

Synthesis of **5** (**P-MePh**)

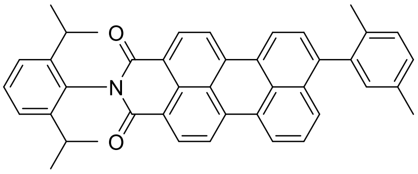

Chemical Formula: $C_{42}H_{35}NO_2$
Molecular Weight: 586

Synthesis was done as described for compound **4**. For the reaction compound **1** (180 mg, 0.32 mmol, 1 eq.), 2,5-dimethylphenylboronic acid (58 mg, 0.38 mmol, 1.2 eq.), $Pd(PPh_3)_4$ (37 mg, 10 mol%) and KF (1 M, 2 mL) in a mixture of 25 mL toluene and 2 mL EtOH were used. After workup the crude material was precipitated from CH_2Cl_2 and MeOH to give 88 mg of product. Estimated purity 80-90% (NMR). This was further purified by silica gel column (cyclohexane/ethyl acetate = 9/1 to 5/1), and best fractions precipitated from CH_2Cl_2 and MeOH. After filtration, the solution was evaporated to give 10 mg (5%) of pure product (red powder). ^{1}H NMR (500 MHz, $CDCl_3$): δ 8.70-8.65 (m, 2H), 8.54 (d, 1H, *J* = 7.8 Hz), 8.52-8.47 (m, 3H), 7.62-7.51 (m, 3H), 7.48 (t, 1H, *J* = 7.8 Hz), 7.34 (d, 2H, *J* = 7.8 Hz), 7.27 (d, 1H, *J* = 7.8 Hz), 7.22 (d, 1H, *J* = 7.8 Hz), 7.12 (s, 1H), 2.78 (sept, 2H, *J* = 6.8 Hz), 2.41 (s, 3H), 2.06 (s, 3H), 1.21-1.16 (m, 12H) ppm. HSQC NMR (300 MHz, $CDCl_3$): δ 132.2, 130.8, 130.2, 129.5, 129.4, 128.9,

128.0, 127.0, 124.0, 123.8, 120.2, 29.2, 24.3, 20.1, 19.8 ppm. Both NMR spectra are shown in Figure S30. Furthermore, Figure S31 contains COSY and Figure S32 NOESY1D spectra. HRMS (MALDI) m/z calcd for $C_{42}H_{35}NO_2+H^+$: 586.2741 $[M+H]^+$; found 586.2726.

Synthesis of **6** (**P-P**)

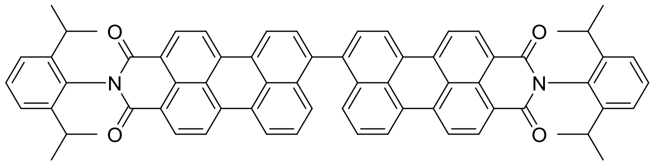

Chemical Formula: $C_{68}H_{52}N_2O_4$
Molecular Weight: 961

In a round bottom flask **P-Br** (compound **2**) (86 mg, 0.15 mmol, 1.1 eq.), and compound **3** (**P-Bpin**) (85 mg, 0.14 mmol, 1 eq.), K_2CO_3 (1 M, 4 mL) and Aliquat 336 (1 drop) was suspended in 4 mL toluene and degassed with a flow of nitrogen for 20 min before addition of $Pd(PPh_3)_4$ (5 mol%). The mixture was stirred at 80 °C for 2 h. After cooling to room temperature, the product was extracted with toluene, washed with deionized water and brine, dried over Na_2SO_4, and concentrated. After purification through a silica gel column (toluene/ethyl acetate from 100/0 to 85/15) and precipitation from CH_2Cl_2 and MeOH 71 mg (53%) of red solid was obtained. ^1H NMR (500 MHz, $CDCl_3$): δ 8.74 (d, 2H, *J* = 8.1 Hz), 8.72 (d, 2H, *J* = 8.1 Hz), 8.67 (d, 2H, *J* = 7.8 Hz), 8.60 (d, 2H, *J* = 8.1 Hz), 8.57-8.53 (m, 4H), 7.76 (d, 2H, *J* = 7.6 Hz), 7.63 (d, 2H, *J* = 8.4 Hz), 7.58-7.53 (m, 2H), 7.49 (t, 2H, *J* = 7.8 Hz), 7.36 (d, 4H, *J* = 7.8 Hz), 2.80 (sept, 4H, *J* = 6.9 Hz), 1.24-1.17 (m, 24H) ppm. Spectra are shown in Figure S28 as in good agreement with previously published NMR.[12] HRMS (MALDI) m/z calcd for $C_{68}H_{52}N_2O_4$: 960.3927 $[M]^+$; found 960.3950.

Synthesis of **7a** (**P-pPh-P**)

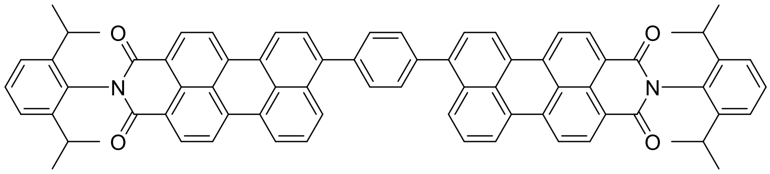

Chemical Formula: $C_{74}H_{56}N_2O_4$
Molecular Weight: 1037

Synthesis was done as described for compound **4**. For the reaction, **P-Br** (compound **2**) (768 mg, 1.37 mmol, 1 eq.), 1,4-phenyldiboronic acid (103 mg, 0.62 mmol, 0.45 eq.), $Pd(PPh_3)_4$ (158 mg, 10 mol%) and KF (1 M, 5 mL) in a mixture of 80 mL toluene and 10 mL EtOH were used. During extraction an emulsion is formed, which had to be filtered. The solid part (around 450 mg) was mainly product, filtrate mainly contained side products. The solid part was purified via multiple silica gel columns (with CH_2Cl_2 as eluent) to give two fractions: 106 mg with an estimated purity 80% (NMR) and 42 mg (6%) of clean product (dark red powder). ^1H NMR (500 MHz, $CDCl_3$): δ 8.71 (dd, 4H, *J* = 8.0, 2.7 Hz), 8.61 (d, 2H, *J* = 8.0 Hz), 8.58 (d, 2H, *J* = 7.5 Hz), 8.56-8.52 (m, 4H), 8.20 (d, 2H, *J* = 8.3 Hz), 7.78-7.41 (m, 6H), 7.71 (t, 2H, *J* = 7.9 Hz), 7.49 (t, 2H, *J* = 7.8 Hz), 7.36 (d, 4H, *J* = 7.8 Hz), 2.80 (sept, 4H, *J* = 6.9 Hz), 1.20 (d, 24H, *J* = 6.9 Hz) ppm. Polarisation transfer used in HSQC and HMBC methods, combined with ^{13}C APT measurment, allowed to determine the following chemical shifts: ^{13}C ($CDCl_3$) 164.2, 145.6, 142.8, 139.7, 137.7, 133.0, 132.2, 131.1, 130.6, 129.6, 129.5, 128.5, 127.3 126.6, 124.1, 124.0, 123.7,

120.3, 29.1, 24.1 ppm. Spectra are shown in Figure S37 and Figure S38. HRMS (MALDI) m/z calcd for $C_{74}H_{56}N_2O_4$+H^+: 1037.4313 $[M+H]^+$; found 1037.3906.

Synthesis of **7b** (**P-mPh-P**)

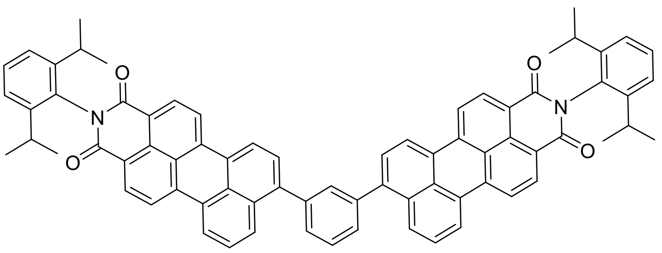

Chemical Formula: $C_{74}H_{56}N_2O_4$
Molecular Weight: 1037,2720

Synthesis was done as described for compound **6**. Initially **P-Br** (compound **2**) (1.10 g, 1.97 mmol), 1,3-benzenediboronic acid (166 mg, 1.00 mmol), $P(Ph_3)_4$ (58 mg, 5 mol%) and Aliquat 336 (1 drop) in a mixture of toluene and 1 M K_2CO_3 (100 mL/14 mL) was stirred for 45 h. As no full conversion was observable more $Pd(PPh_3)_4$ (29 mg, 0.025 mmol) and 1,3-benzenediboronic acid (42.1 mg, 0.25 mmol) were added and the mixture was stirred for another 21 h. Compound **7b** (117 mg, 11%) was obtained as a red powder after purification through a silica gel column (toluene/acetone = 99/1) and several recrystallization steps (CH_2Cl_2/toluene and MeOH). ^{1}H NMR (500 MHz, $CDCl_3$): δ 8.71-8.67 (m, 4H), 8.58 (d, 2H, *J* = 8.0 Hz), 8.56-8.49 (m, 6H), 8.18 (2H, d, *J* = 8.4 Hz), 7.78-7.65 (m, 8H), 7.49 (t, 2H, *J* = 7.8 Hz), 7.35 (d, 4H, *J* = 7.8 Hz), 2.77 (sept, 4H, *J* = 6.9 Hz), 1.18 (d, 24H, *J* = 6.9 Hz) ppm. ^{13}C-APT NMR (75 MHz, $CDCl_3$): δ 164.1, 145.9, 142.9, 140.5, 137.8, 132.3, 131.2, 129.8, 129.7, 129.6, 128.6, 127.4, 124.2, 123.7, 121.3, 120.6, 120.4, 29.3, 24.2 ppm. Spectra are shown in Figure S39 and Figure S40. HRMS (MALDI) m/z calcd for $C_{74}H_{56}N_2O_4$+H^+: 1037.4313 $[M+H]^+$; found 1037.4111.

Synthesis of **7c** (**P-oPh-P**)

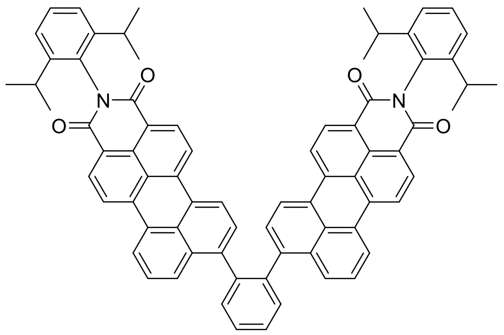

Chemical Formula: $C_{74}H_{56}N_2O_4$
Molecular Weight: 1037

Synthesis was done as described for compound **6**. Using 500 mg (0.89 mmol) of **2** (**P-Br**), 148 mg (0.45 mmol) of 1,2-benzenediboronic acid, bis(pinacol)ester, $P(Ph_3)_4$ (26 mg, 5 mol%) and Aliquat 336 (1 drop) in a mixture of toluene and 1 M K_2CO_3 (100 mL/7 mL), compound **7c** (139 mg, 30%) was obtained as a dark red powder after purification through several silica gel columns (CH_2Cl_2/CH in different ratios) and several recrystallization steps (CH_2Cl_2 and MeOH). ^{1}H NMR (500 MHz, $CDCl_3$): δ 8.46 (d, 1H, *J* = 7.8 Hz), 8.35 (d, 2H, *J* = 7.2 Hz), 8.30 (d, 1H, *J* = 7.8 Hz), 8.23 (d, 1H, *J* = 7.8 Hz), 8.20 (1H, d, *J* = 7.8 Hz), 8.10-7.92 (m, 5H), 7.90 (d, 1H, *J* = 8.4 Hz), 7.82-7.74 (m, 2H), 7.72-7.67 (m, 2H), 7.67-7.62 (m, 2H), 7.58-7.54 (m, 1H), 7.48-7.42 (m, 3H), 7.33 (t, 1H, *J* = 8.0 Hz), 7.30 (d, 4H, *J* = 7.8 Hz), 7.18 (d, 1H, *J* = 7.7 Hz), 2.71-2.59 (4H, m), 1.19-1.06 (24H, m) ppm. ^{13}C-APT NMR (75 MHz, $CDCl_3$): δ 163.9, 145.7, 142.1, 141.9, 139.6, 139.5, 137.3, 137.1, 137.0, 133.4, 132.7, 131.9, 131.8, 131.7, 131.0, 130.3, 129.7, 129.6, 129.5,

129.4, 129.3, 128.5, 128.5, 128.4, 128.1, 128.0, 127.2, 126.7, 126.7, 126.6, 124.1, 123.9, 123.8, 123.0, 122.9, 121.0, 120.9, 120.9, 120.3, 120.1, 120.0, 29.3, 29.2, 24.1 ppm. Spectra are shown in Figure S41 and Figure S42. HRMS (MALDI) m/z calcd for $C_{74}H_{56}N_2O_4+H^+$: 1037.4313 $[M+H]^+$; found 1037.4606.

Synthesis of **7d** (**P3Ph**)

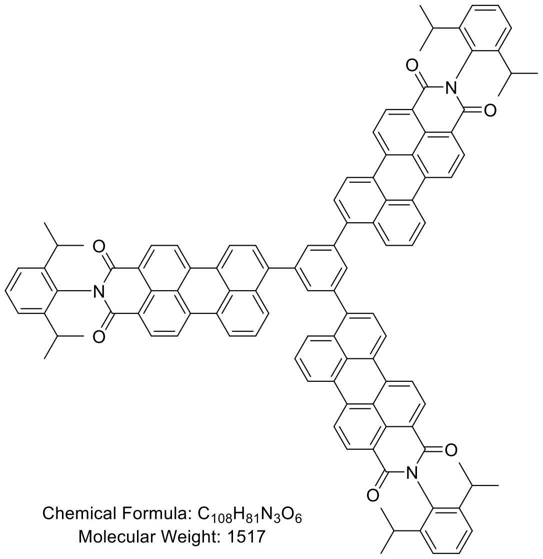

Chemical Formula: $C_{108}H_{81}N_3O_6$
Molecular Weight: 1517

A mixture of THF (10 mL), EtOH (2 mL) and KF (1 M, 2 mL) solution was degassed before the addition of compound **2** (**P-Br**) (200 mg, 0.36 mmol) and 1,3,5-phenyltriboronic acid, tris(pinacol)ester (40 mg, 0.088 mmol) and $Pd(PPh_3)_4$ (5 mol%). The mixture was heated at 70 °C for 20 h. The solution was cooled to room temperature, extracted with toluene, washed with deionized water and brine, dried over Na_2SO_4, and concentrated. Compound **7d** (55 mg, 8%) was obtained as a dark red powder after purification through several silica gel columns (CH_2Cl_2/MeOH and CH_2Cl_2/CH in different ratios) and several recrystallization/precipitation steps (CH_2Cl_2/MeOH and CH_2Cl_2/n-hexane). ^1H NMR (500 MHz, $CDCl_3$): δ 8.67-8.62 (m, 6H), 8.55 (d, 3H, *J* = 8.0 Hz), 8.49 (d, 3H, *J* = 7.7 Hz), 8.47-8.41 (m, 6H), 8.31 (d, 3H, *J* = 8.4 Hz), 7.92 (s, 3H), 7.84 (d, 3H, *J* = 7.8 Hz), 7.70 (t, 3H, *J* = 8.1 Hz), 7.47 (t, 3H, *J* = 7.8 Hz), 7.34 (d, 6H, *J* = 7.8 Hz), 2.78 (sept, 6H, *J* = 6.8 Hz), 1.18 (d, 36H, *J* = 6.8 Hz) ppm. ^{13}C-APT NMR (75 MHz, $CDCl_3$): δ 164.0, 145.8, 142.2, 141.0, 137.5, 137.3, 132.7, 132.2, 131.2, 131.1, 130.6, 129.8, 129.6, 129.3, 129.0, 128.8, 128.7, 127.6, 127.0, 124.2, 123.6, 121.3, 120.6, 120.5, 29.3, 24.2 ppm. Spectra are shown in Figure S43. HRMS (MALDI) m/z calcd for $C_{108}H_{81}N_3O_6$: 1516.6159 $[M]^+$; found 1516.4862.

Synthesis of **7e** (**P-MePh-P**)

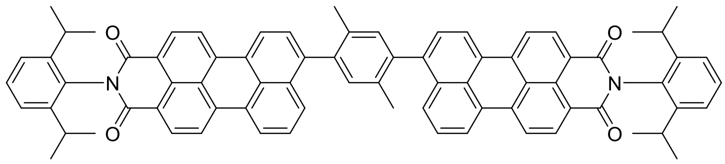

Chemical Formula: $C_{76}H_{60}N_2O_4$
Molecular Weight: 1065

Synthesis was done as described for compound 6. For the reaction, compound **2** (**P-Br**) (424 mg, 0.76 mmol, 1 eq.), 2,5-dimethyl-1,4-phenylenediboronic acid pinacol ester (123 mg, 0.34 mmol, 0.45 eq.), $P(Ph_3)_4$ (87 mg, 10 mol%) and Aliquat 336 (1 drop) in a mixture of toluene and 1 M K_2CO_3 (25 mL/9 mL) were used. Purification was done by column chromatography (SiO_2, EtOAc/cyclohexane eluent) followed by precipitation from CH_2Cl_2/MeOH. Compound **7e** (47 mg, 13%) was obtained as a dark red powder. ^1H NMR (500 MHz, $CDCl_3$): δ 8.74-8.69 (m, 4H), 8.64-8.60 (m, 2H), 8.60-8.52 (m, 6H), 7.81 (d, 1H, *J* = 8.3 Hz), 7.78 (d, 1H, *J* = 8.3 Hz), 7.73-7.64 (m, 4H), 7.49 (t, 3H, *J* = 7.8 Hz), 7.38-7.32 (m, 6H),

2.85-2.75 (m, 4H), 2.17 (s, 6H), 1.23-1.17 (m, 24H) ppm. ^{13}C-APT NMR (75 MHz, $CDCl_3$): δ 164.2, 145.9(2), 145.8(7), 143.0, 142.9, 139.4(2), 139.4, 137.9, 137.8, 134.4, 134.3, 134.2, 133.2, 132.3, 132.2(8), 132.1, 131.2, 130.8, 129.8, 129.7, 129.6, 129.5, 128.9, 128.4, 128.3(7), 127.4, 127.3(9), 127.2, 124.2, 124.1, 123.8, 123.7, 121.3, 121.2, 121.1(6), 120.6, 120.3, 53.6, 31.1, 29.3, 24.2, 19.9 ppm. Spectra are shown in Figure S44. HRMS (MALDI) m/z calcd for $C_{76}H_{60}N_2O_4$: 1064.4553 $[M]^+$; found 1064.4573.

Synthesis of **7f** (**P-HexPh-P**)

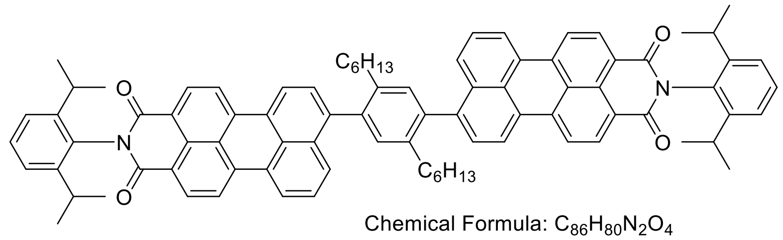

Chemical Formula: $C_{86}H_{80}N_2O_4$
Molecular Weight: 1205,5960

In a round bottom flask to compound **3** (**P-BPin**) (760 mg, 1.25 mmol, 1 eq.) and 2,5-bis(hexyl)-1,4-dibromobenzene (202 mg, 0.50 mmol, 0.4 eq.) was suspended in a mixture of 25 mL toluene and 5 mL EtOH. To this mixture KF (1 M, 3 mL) was added, and the solution was degassed by a flow of nitrogen for 20 min. Then $Pd(PPh_3)_4$ (114 mg, 10 mol%) was added and the mixture refluxed 16 h (under nitrogen). The solution was cooled to room temperature, extracted with toluene, washed with deionized water and brine, dried over Na_2SO_4, and concentrated. Purification was done by silica gel column (Ethyl acetate/cyclohexane = 3/1) followed by and another silica gel column (CH_2Cl_2/cyclohexane = 1/1). Crystallization on column was observed (thus - peak tailing). Compound was precipitated from CH_2Cl_2 and MeOH to give bright red powder (135 mg, 22%). ^{1}H NMR (500 MHz, $CDCl_3$): δ 8.74-8.69 (m, 4H), 8.64-8.60 (m, 2H), 8.60-8.53 (m, 6H), 7.81-7.76 (m, 2H), 7.73-7.63 (m, 4H), 7.49 (t, 2H, *J* = 7.9 Hz), 7.35 (d, 2H, *J* = 7.9 Hz), 7.33 (s, 1H), 7.32 (s, 1H), 2.85-2.74 (m, 4H), 2.56-2.47 (m, 2H), 2.46-2.37 (m, 2H), 1.49-1.40 (m, 4H), 1.20 (d, 24H, *J* = 6.8 Hz), 1.14-1.00 (m, 12H), 0.73-0.68 (m, 6H) ppm. ^{13}C NMR (75 MHz, $CDCl_3$): δ 164.2, 145.90, 145.88, 143.1, 143.0, 139.0, 138.9, 137.93, 137.91, 137.8, 133.74, 133.68, 132.3, 131.4, 131.2, 130.8, 129.8, 129.7, 129.65, 129.60, 128.8, 128.7, 128.65, 128.4, 127.3, 127.2, 124.2, 124.1, 123.6, 123.5, 121.2, 121.1, 120.6, 120.3, 33.1, 31.5, 31.1, 29.3, 29.1, 29.0, 24.2, 22.5, 14.1 ppm. Spectra in $CDCl_3$ are shown Figure S45 (^{1}H and ^{13}C), Figure S36 (ROESY). Spectra in toluene-d8 are shown in Figure S34 (^{1}H and COSY) and Figure S35 (^{1}H at 25, 50 and 80°C). HRMS (MALDI) m/z calcd for $C_{86}H_{80}N_2O_4$+H^+: 1205.6191 $[M+H]^+$; found 1205.6145.

Synthesis of **7g** (**P-DecOPh-P**)

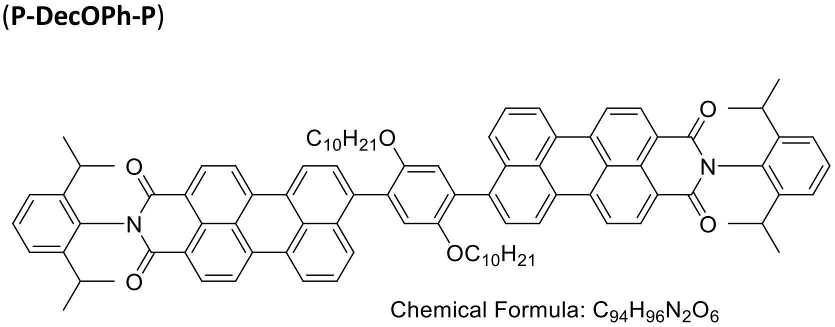

Chemical Formula: $C_{94}H_{96}N_2O_6$
Molecular Weight: 1350

Synthesis was done as described for compound **4**. For the reaction, compound **3** (**P-BPin**) (600 mg, 0.99 mmol, 1 eq.), 1,4-dibromo-2,5-bis(decyloxy)benzene (226 mg, 0.41 mmol, 0.42 eq.), $Pd(PPh_3)_4$ (76

mg, 10 mol%) and KF (1 M, 3 mL) in a mixture of 45 mL toluene and 5 mL EtOH were used. Crude product was purified by silica gel column (dry loading, cyclohexane/ethyl acetate = 9/1 to 5/1), however it was discovered that the product has significant tailing in this eluent. Further purifications were done with CH_2Cl_2 as eluent and in the end the product was precipitated from CH_2Cl_2 and MeOH. The final product is a bright red powder. No yield is reported for this synthesis, because it was repeated multiple times and the half-pure product fractions from these runs united and purified together.
^{1}H NMR (500 MHz, $CDCl_3$): δ 8.74-8.69 (m, 4H), 8.64-8.60 (m, 2H), 8.60-8.52 (m, 6H), 7.98 (d, 1H, *J* = 8.4 Hz), 7.95 (d, 1H, *J* = 8.4 Hz), 7.76 (d, 1H, *J* = 7.7 Hz), 7.73 (d, 1H, *J* = 7.7 Hz), 7.71-7.63 (m, 2H), 7.49 (t, 2H, *J* = 7.9 Hz), 7.35 (d, 2H, *J* = 7.9 Hz), 7.13 (s, 1H), 7.12 (s, 1H), 3.94-3.86 (m, 4H), 2.79 (sept, 6H, *J* = 6.8 Hz), 1.50-1.42 (m, 4H), 1.20 (d, 24H, *J* = 6.8 Hz), 1.16-1.09 (m, 4H), 1.07-0.93 (m, 24H), 0.78 (t, 6H, *J* = 7.3 Hz) ppm. ^{13}C NMR (75 MHz, $CDCl_3$): δ 164.2, 150.9, 145.9, 145.8, 138.1, 137.9, 133.3, 132.3, 131.2, 130.8, 130.1, 129.3, 129.6, 129.0, 128.9, 128.3, 127.2, 127.0, 124.2, 124.0, 123.7, 121.1, 121.0, 120.4, 120.3, 117.0, 69.9, 31.9, 29.6, 29.5, 29.4, 29.3, 26.0, 24.2, 22.7, 14.2 ppm. ^{1}H and ^{13}C spectra are shown in Figure S46. HRMS (MALDI) m/z calcd for $C_{94}H_{96}N_2O_6$: 1349.7302 $[M]^+$; found 1349.6982.

S7. NMR spectra

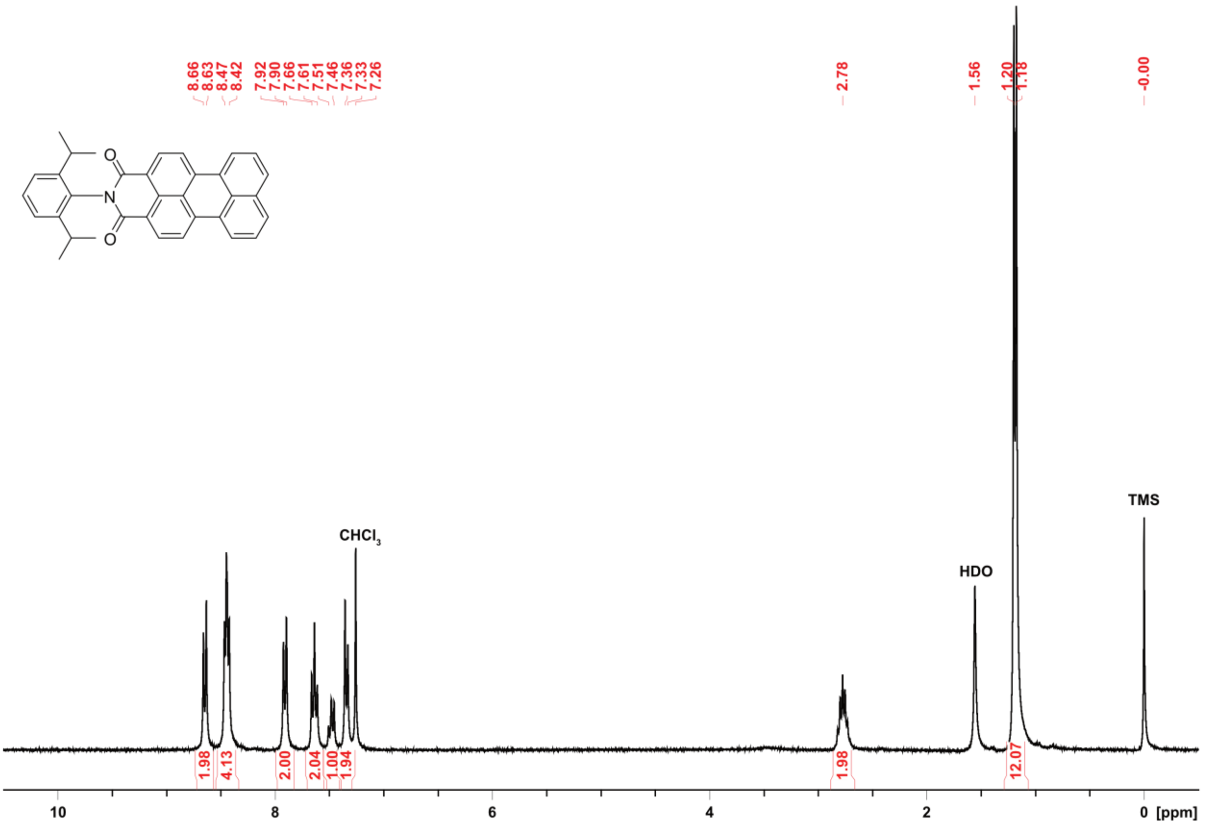

Figure S25: ^{1}H NMR (300 MHz, $CDCl_3$) spectrum of perylene monoimide **1**.

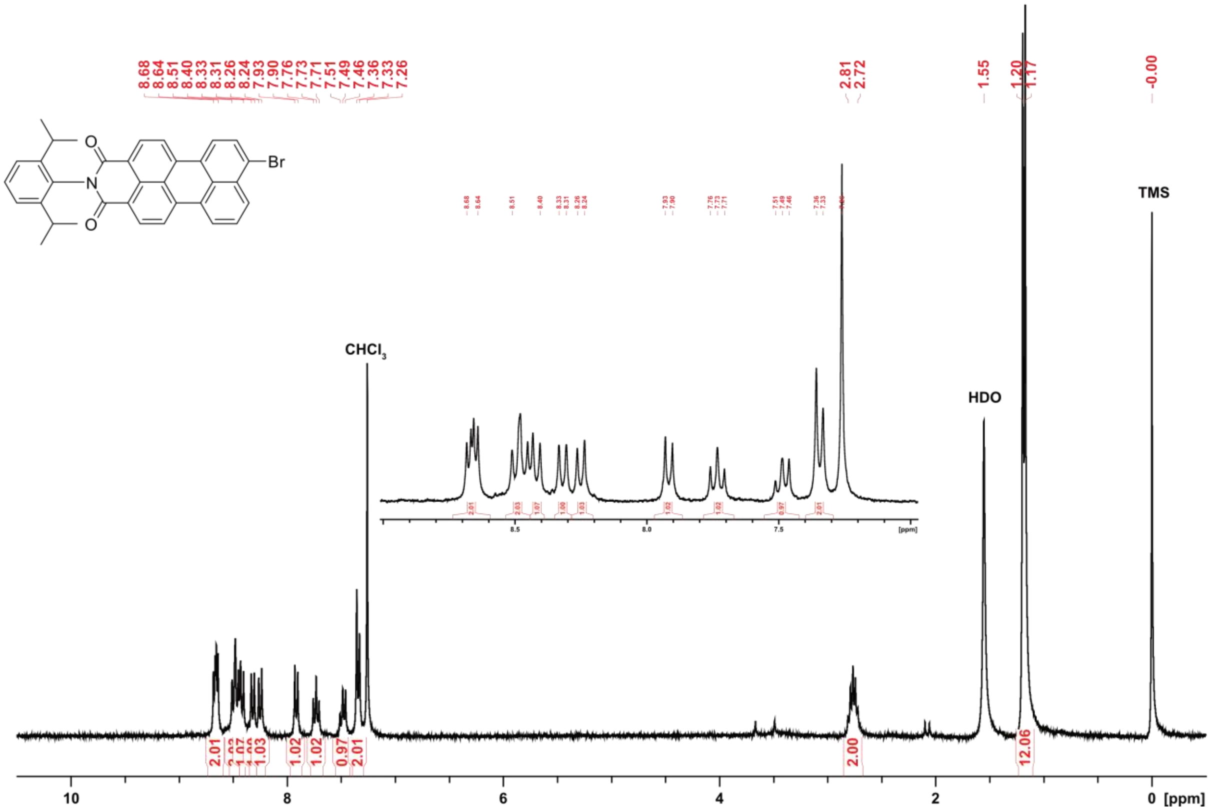

Figure S26: ^{1}H NMR (300 MHz, $CDCl_3$) spectrum of perylene monoimide bromide **2**.

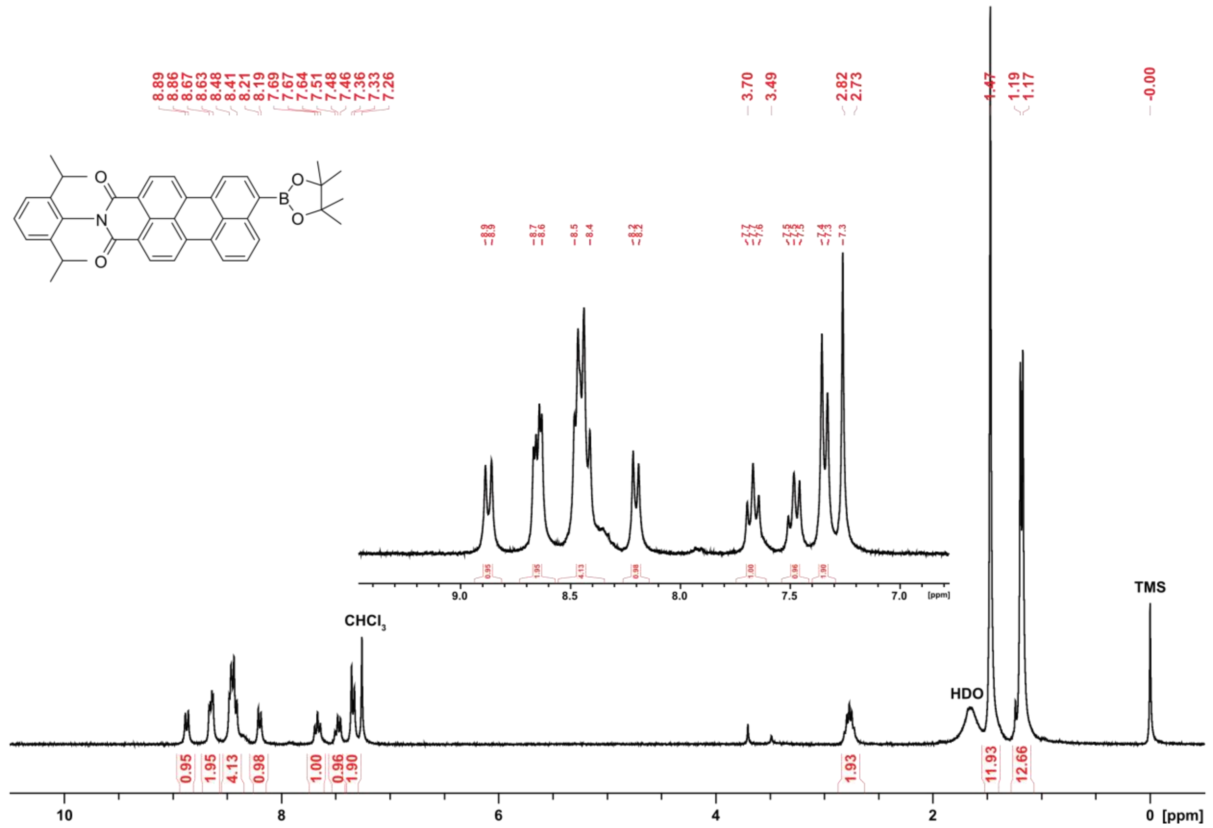

Figure S27: ^{1}H NMR (300 MHz, $CDCl_3$) spectrum of perylene monoimide pinacol ester **3**.

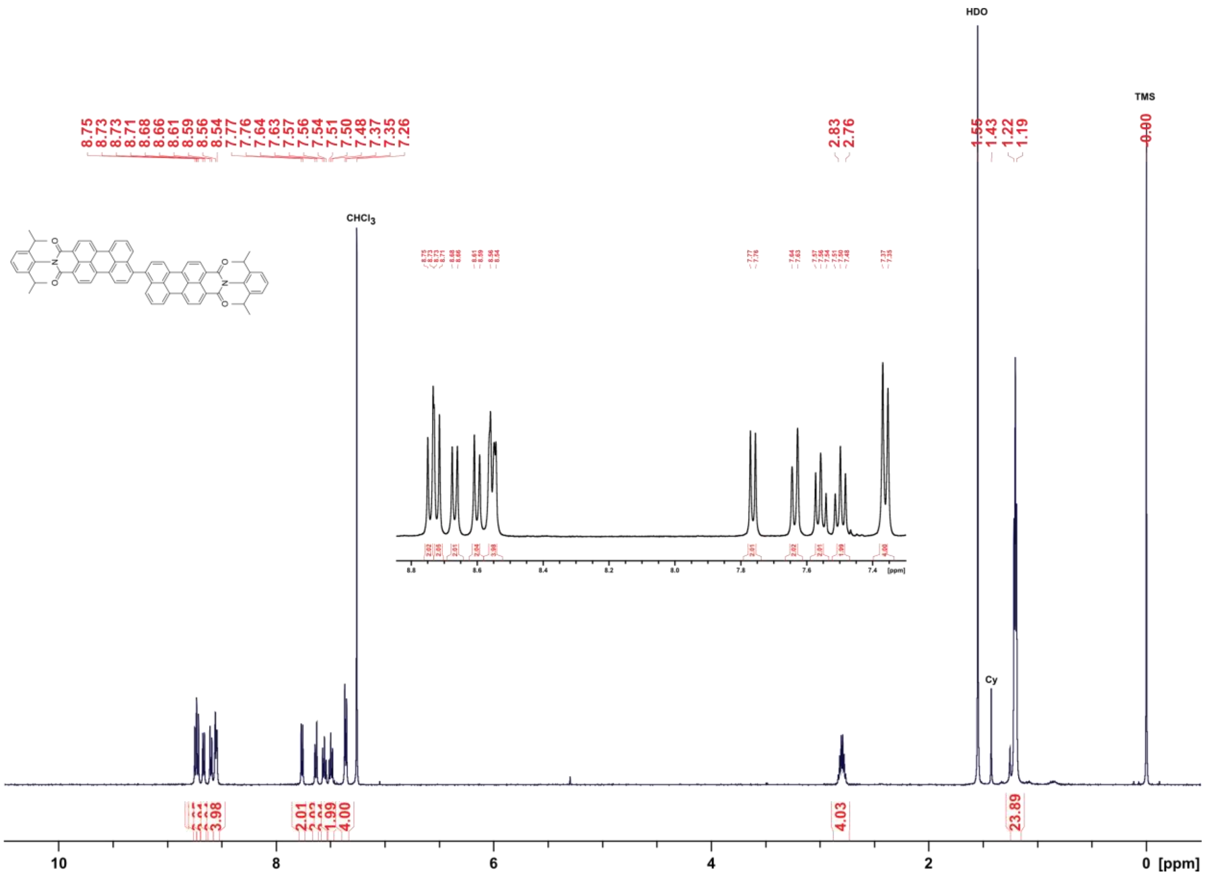

Figure S28: ^{1}H NMR (500 MHz, $CDCl_3$) spectrum of compound **6**. Cy – cyclohexan.

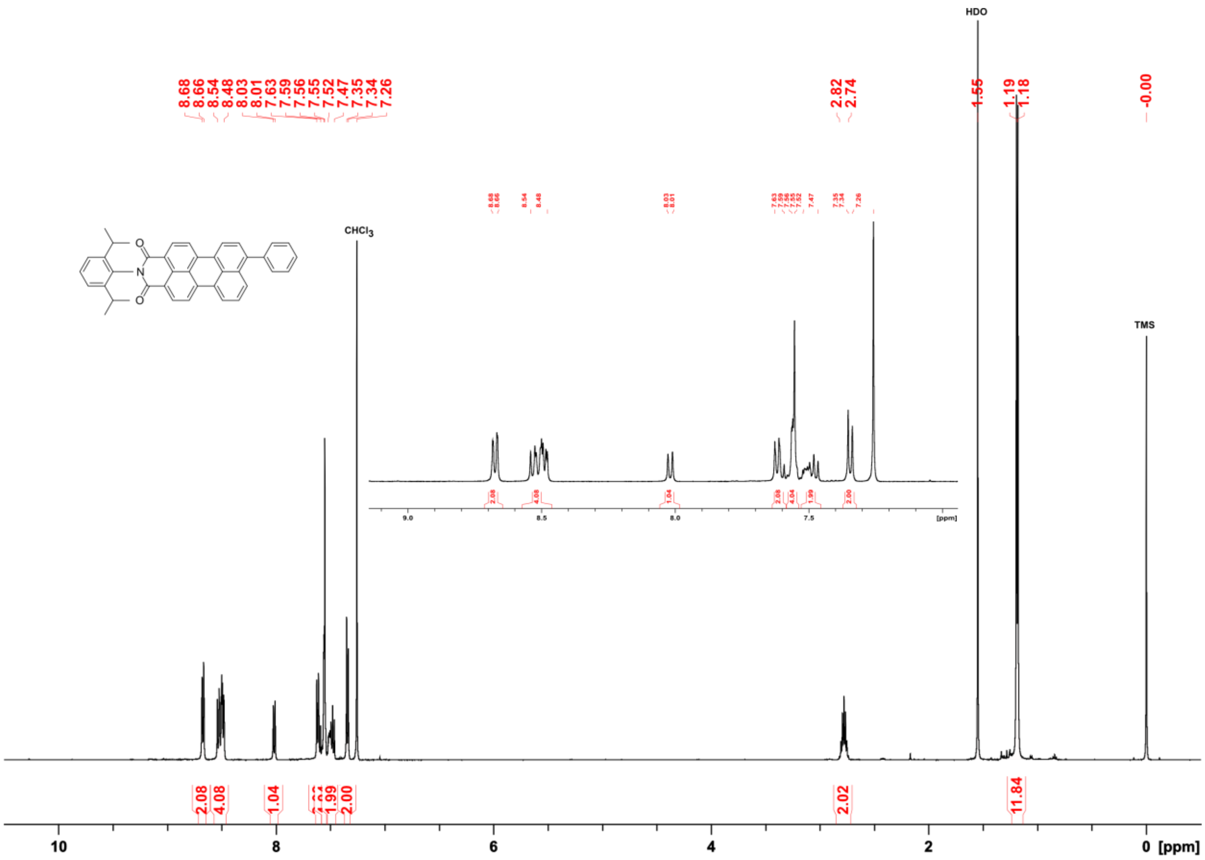

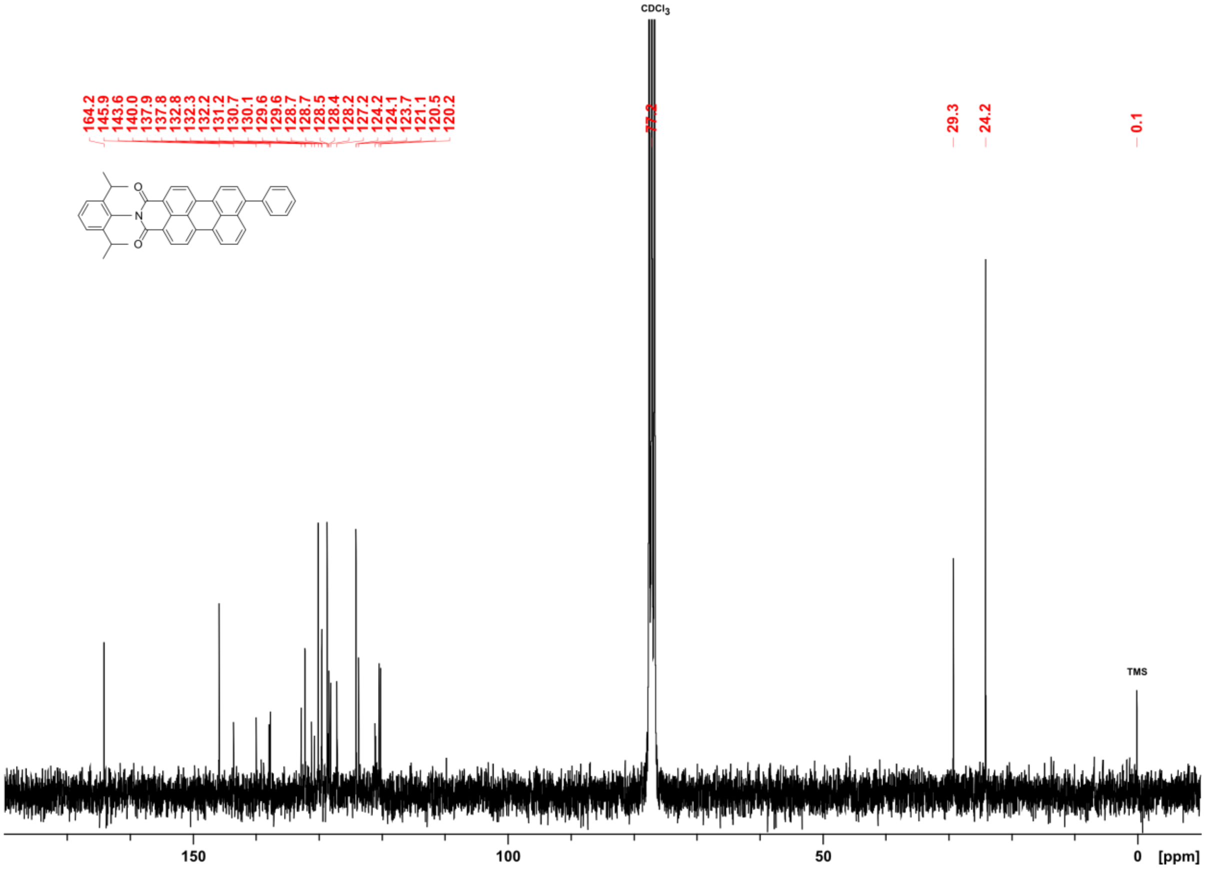

Figure S29: ^{1}H (500 MHz, $CDCl_3$) and ^{13}C (75 MHz, $CDCl_3$) spectra of compound **4**.

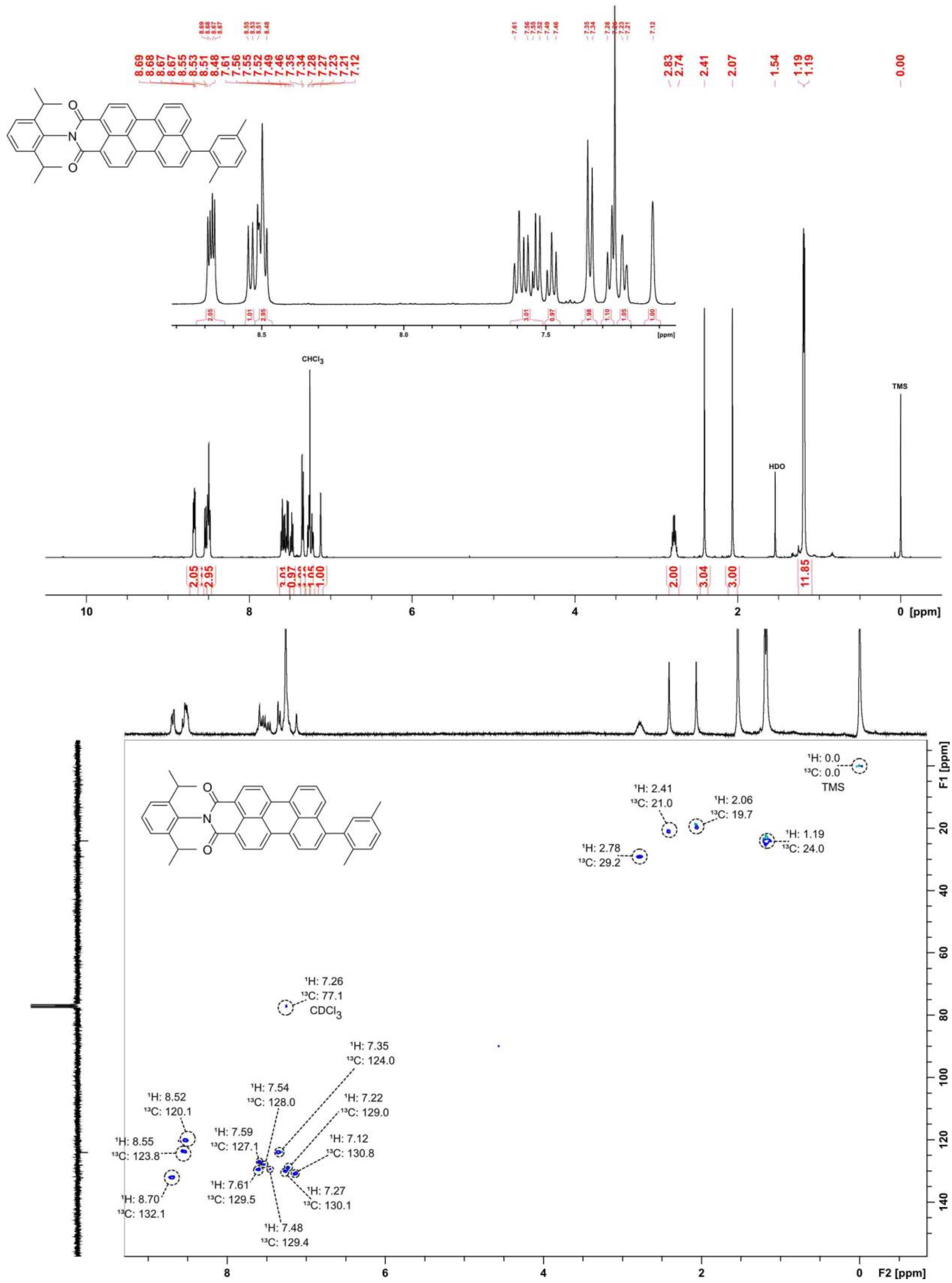

Figure S30: ^{1}H (500 MHz, $CDCl_3$) and HSQC (300 MHz, $CDCl_3$) spectra of compound **5**.

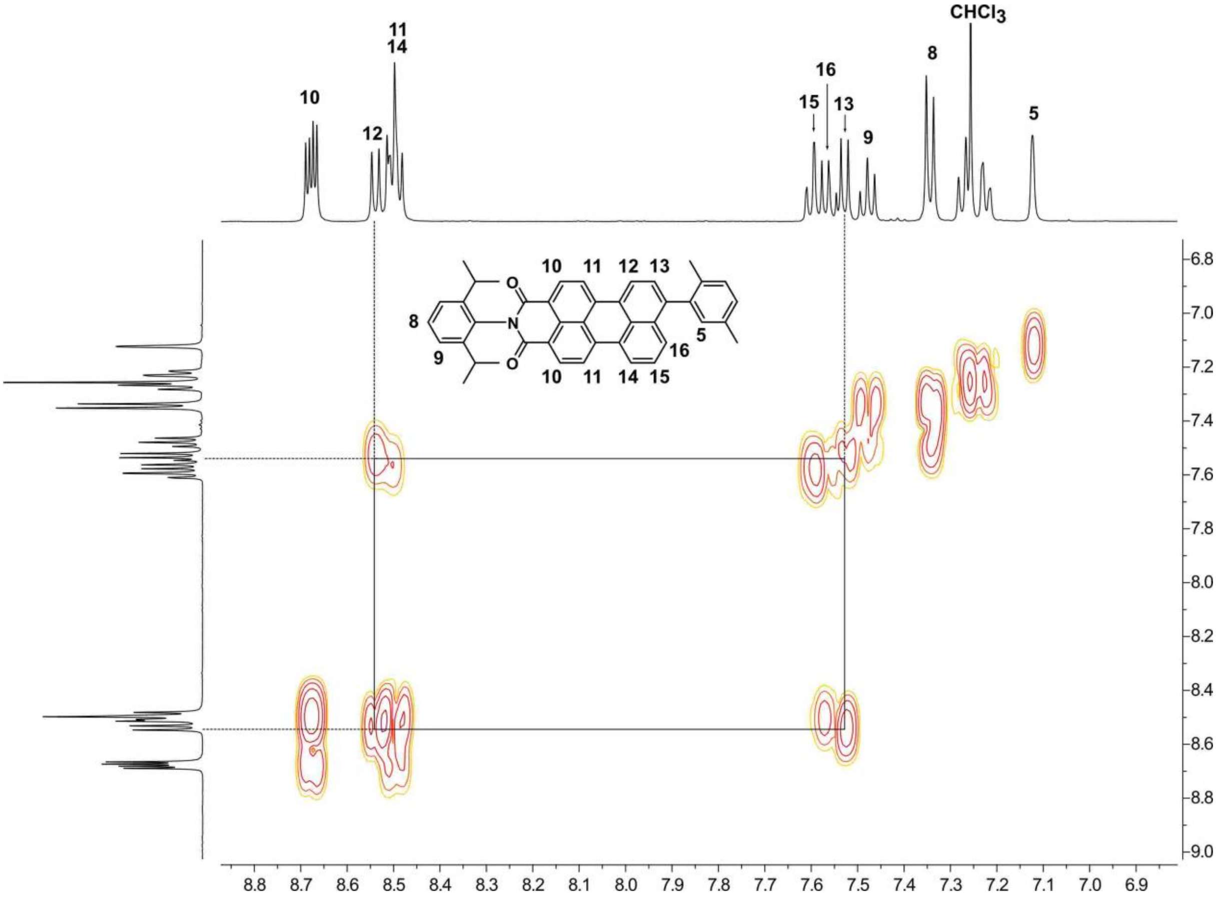

Figure S31: COSY (500 MHz, $CDCl_3$) spectrum of compound **5**.

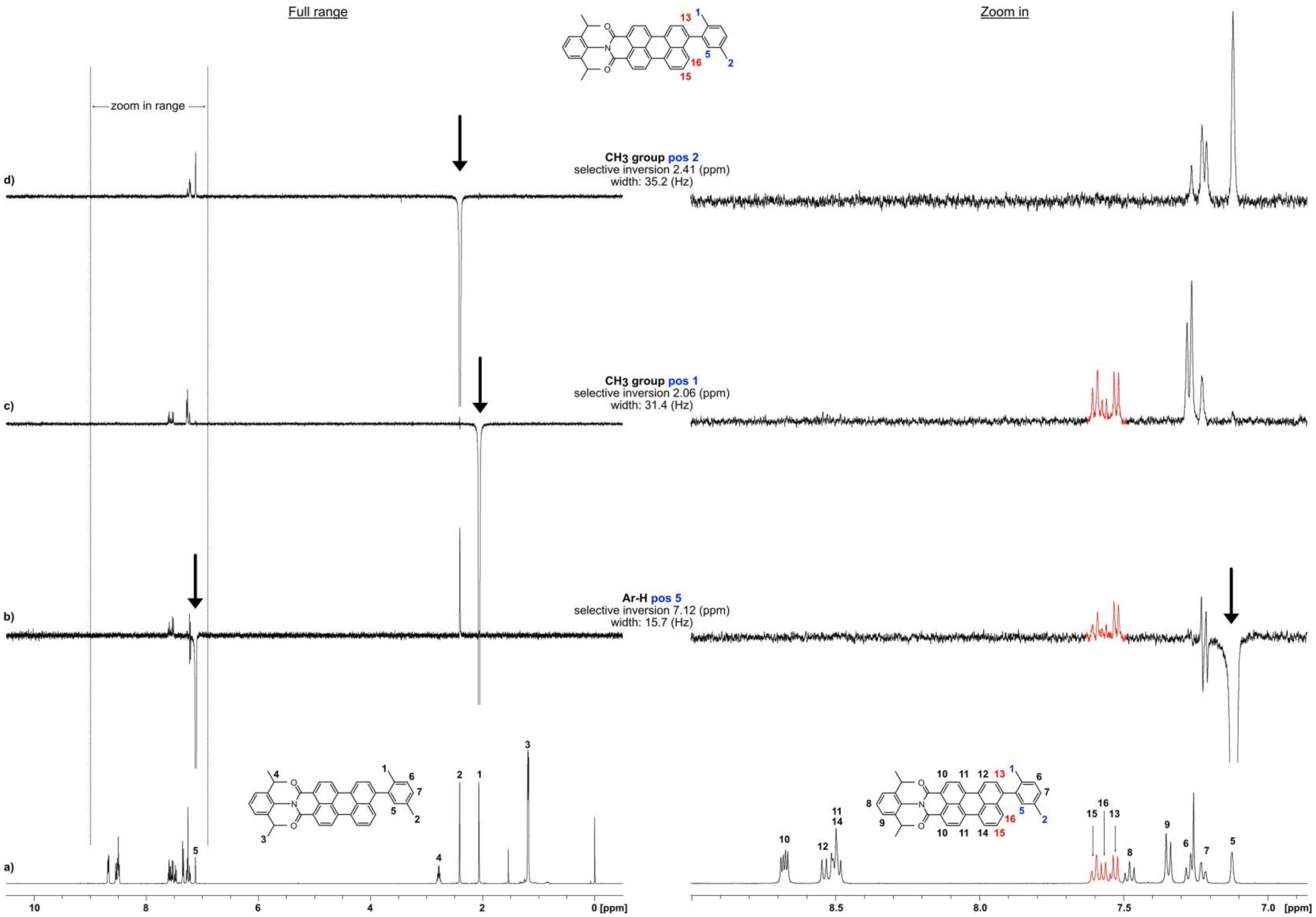

Figure S32: ^{1}H (500 MHz, $CDCl_3$, trace a) spectrum and NOESY1D (500 MHz, $CDCl_3$, mixing time = 500 ms, traces b-d) of compound **5**. Selective inversion position indicated at the respective spectral trace.

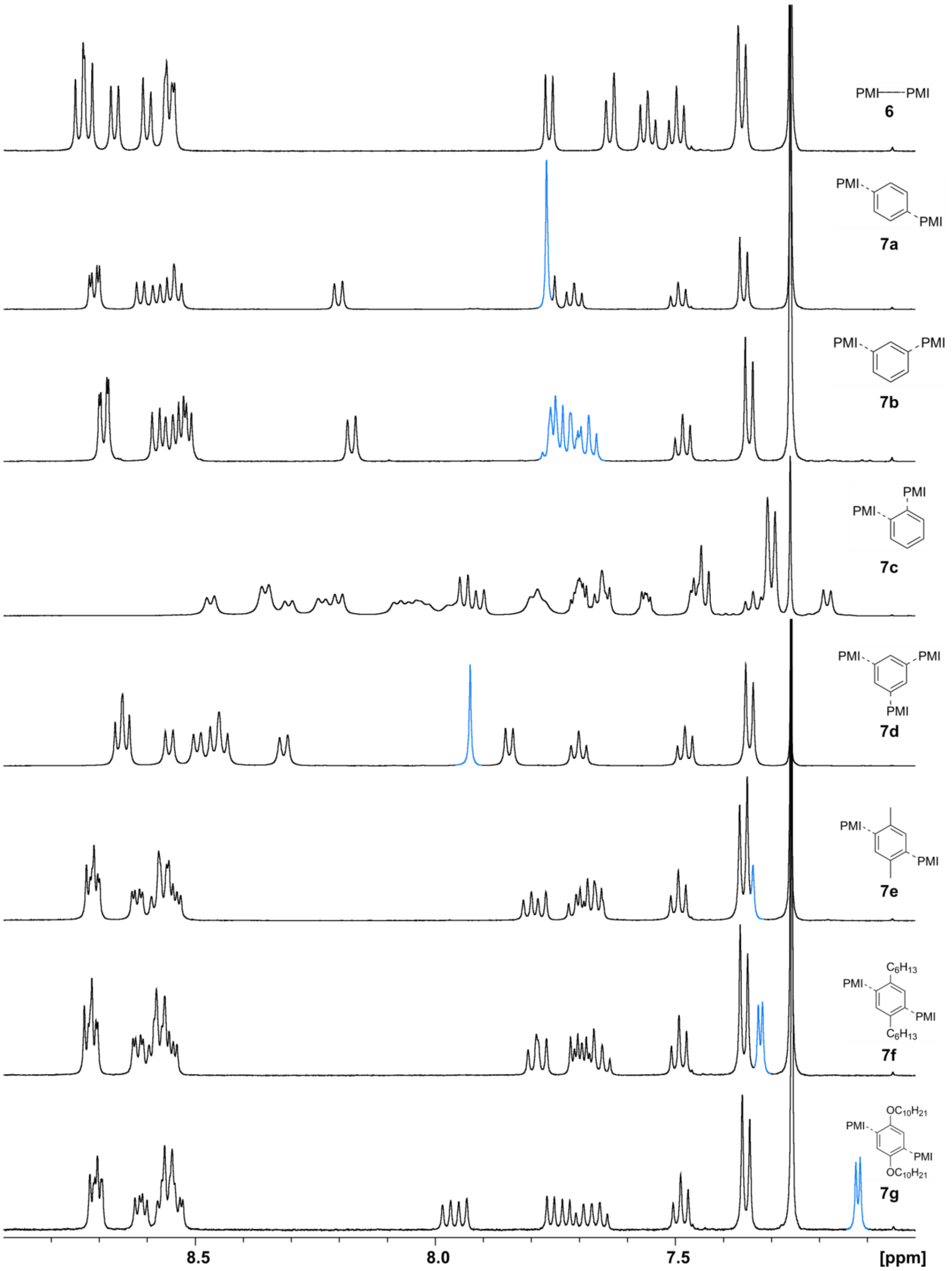

Figure S33: ^{1}H (500 MHz, $CDCl_3$) spectra of compounds **6**, **7a-g**. With blue color are indicated the signals from the phenyl linker (with the exception for molecule **6**, which has no linker, and molecule **7c**, where the signals from the linker cannot be assigned doubtlessly).

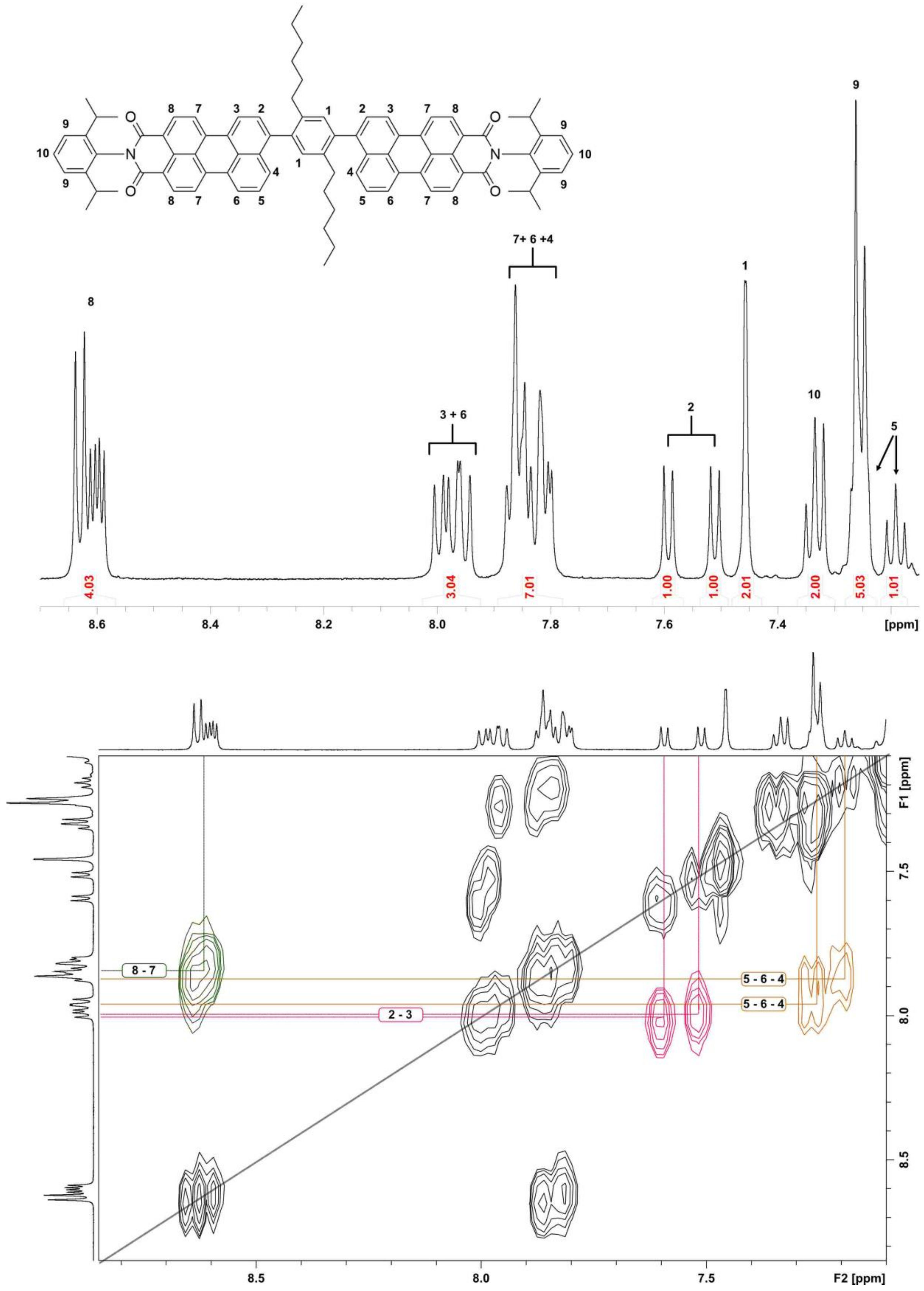

Figure S34: ^{1}H and COSY (500 MHz, toluene-d8) spectra of compound **7f**.

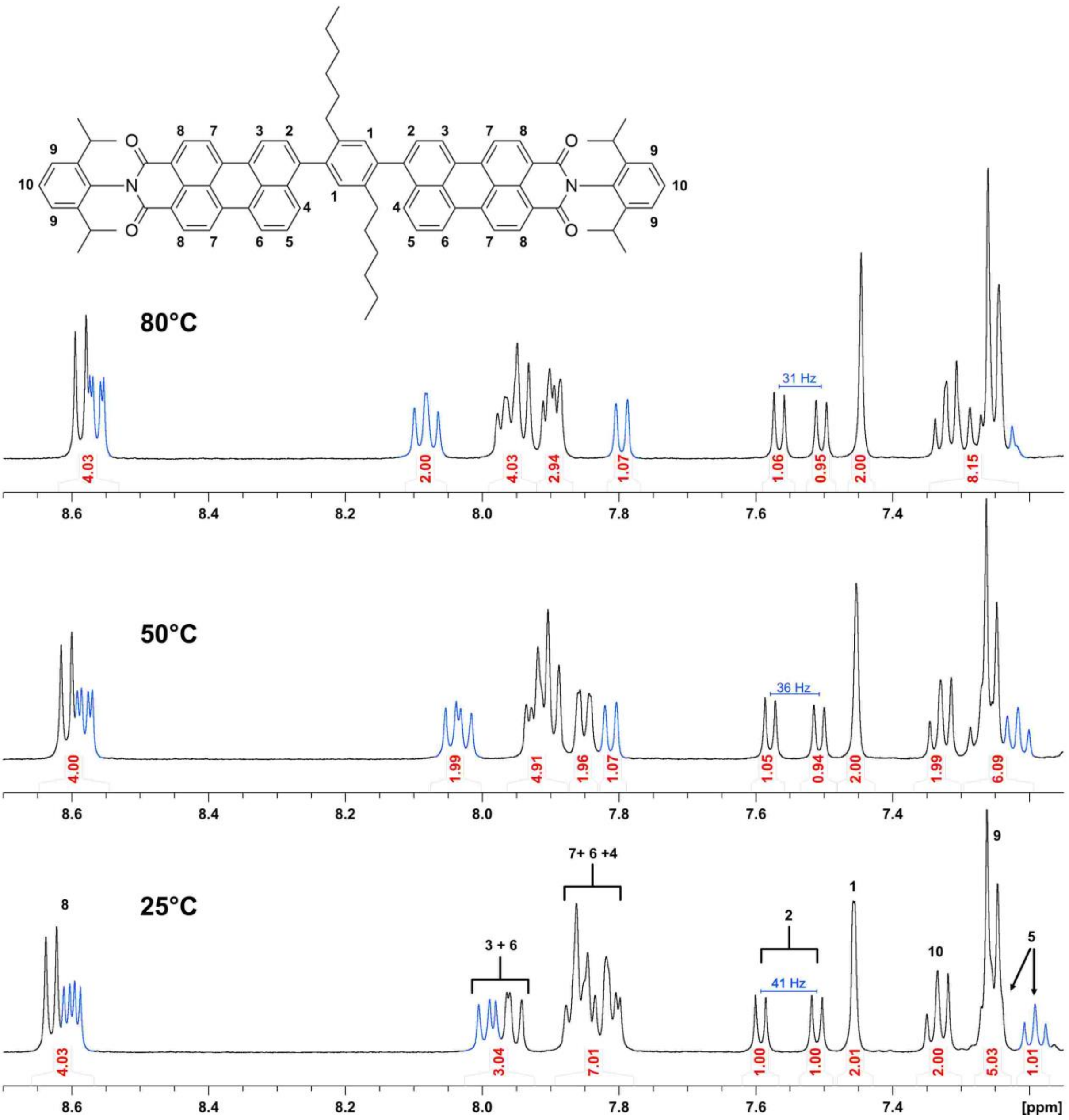

Figure S35: ^{1}H (500 MHz, toluene-d8) spectra of compound **7f** measured at the indicated temperature. For the signal assignment see COSY in the previous page. The largest observable changes are indicated in blue.

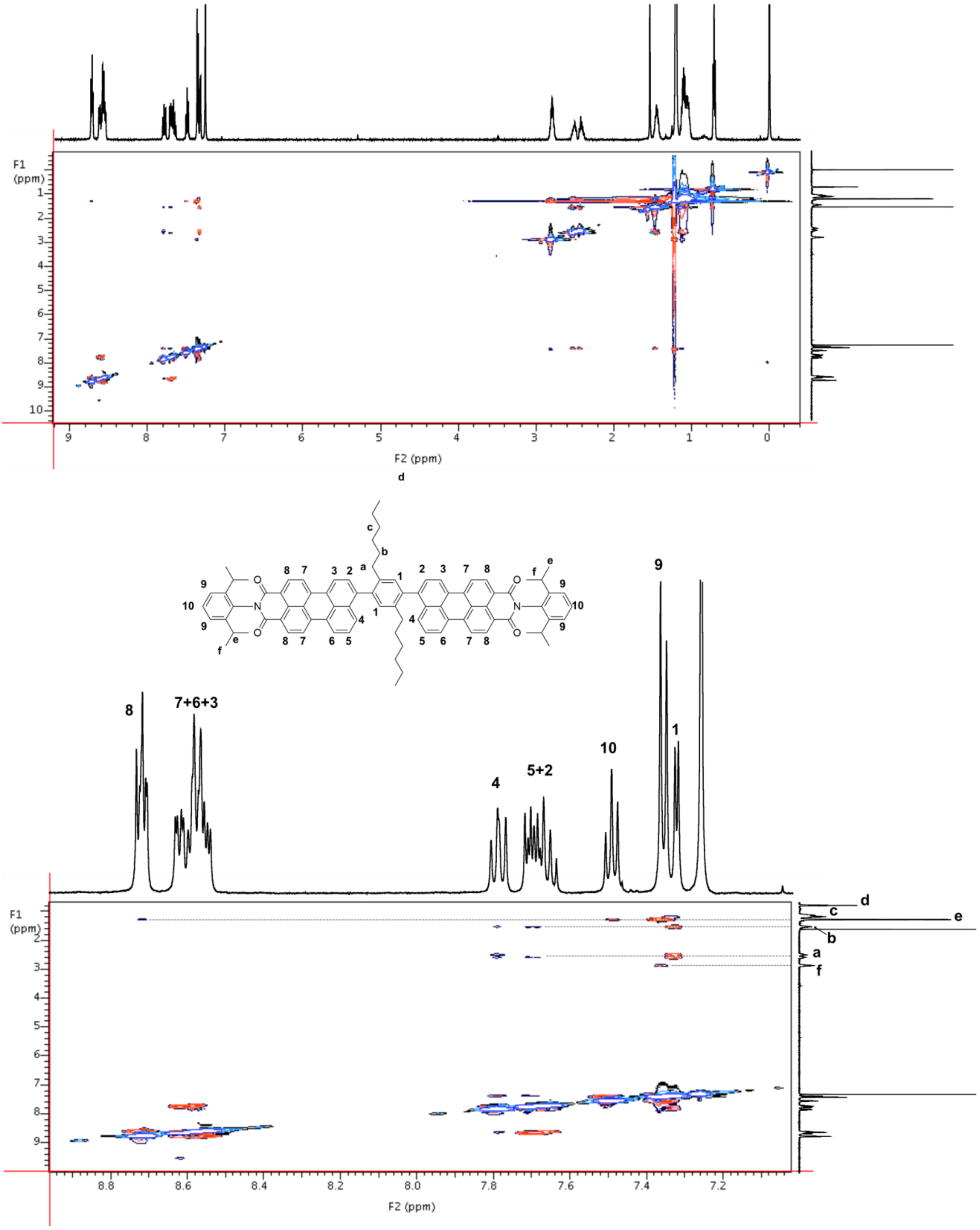

Figure S36: ^{1}H ROESY (500 MHz, $CDCl_3$) spectra of compound **7f**.

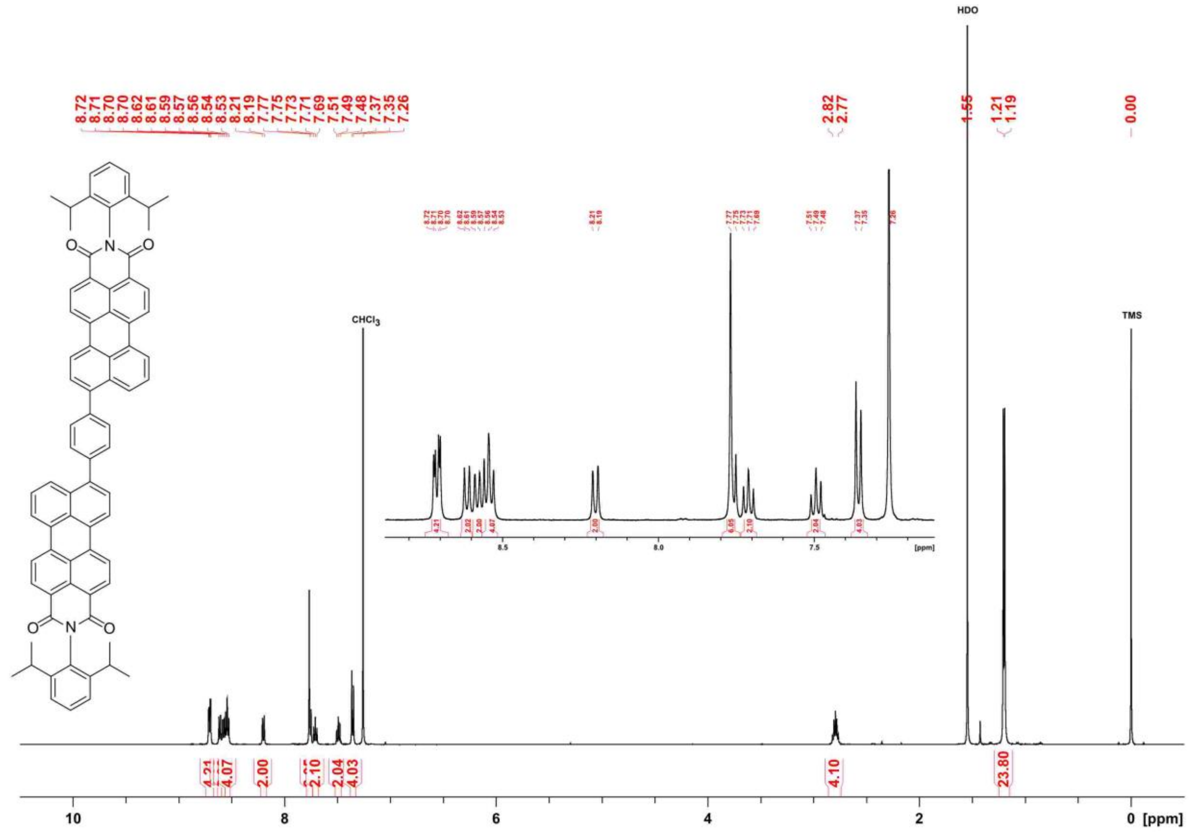

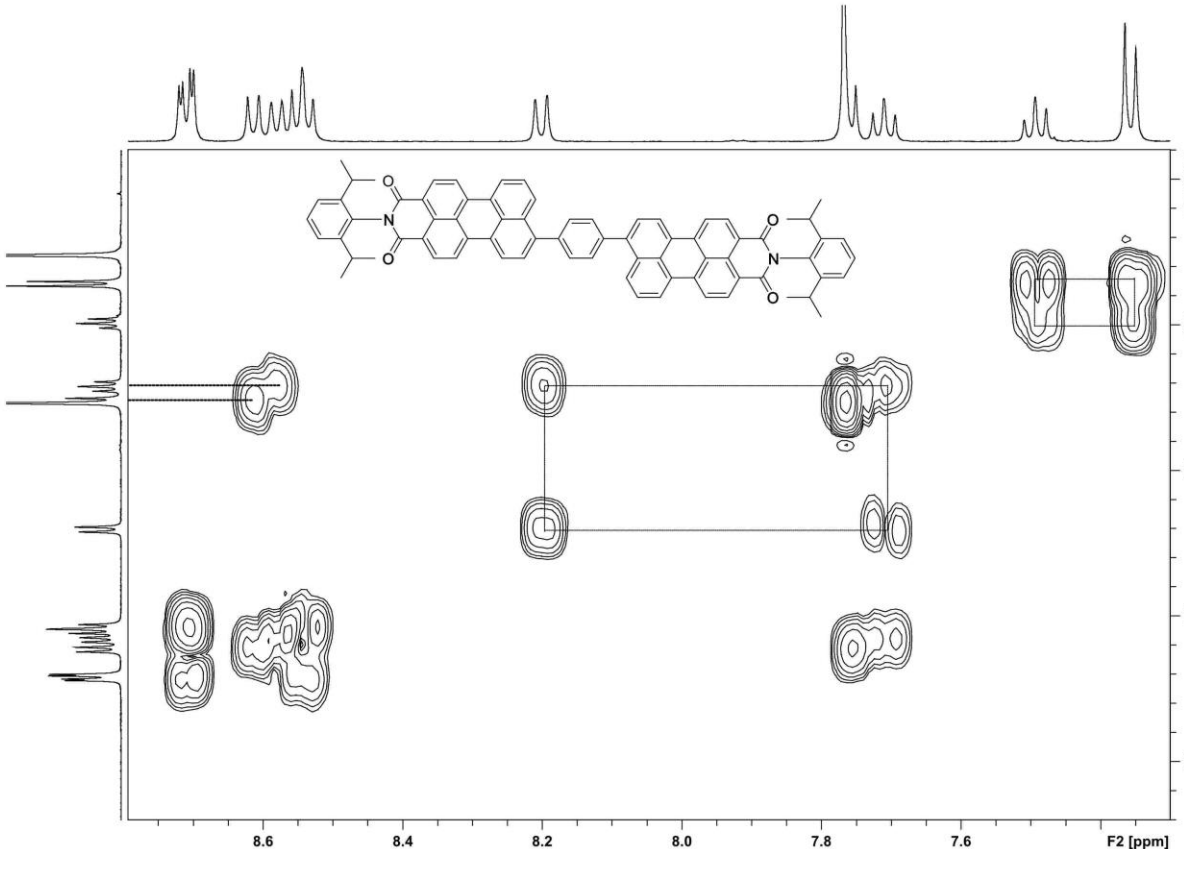

Figure S37: ^{1}H (500 MHz, $CDCl_3$) and COSY (500 MHz, $CDCl_3$) spectra of compound **7a**.

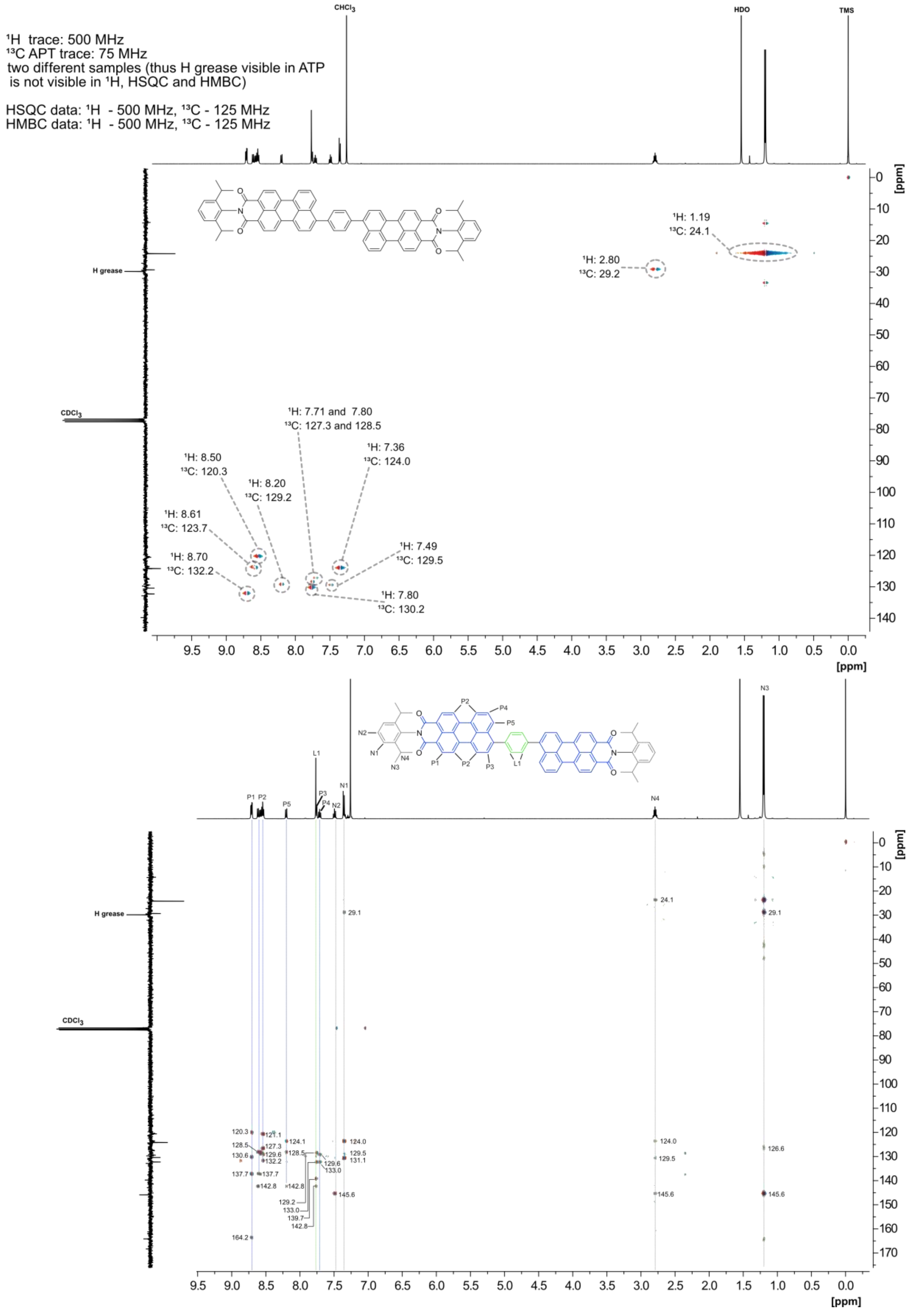

Figure S38: HSQC (500 MHz, $CDCl_3$) and HMBC (500 MHz, $CDCl_3$) spectra of compound **7a**.

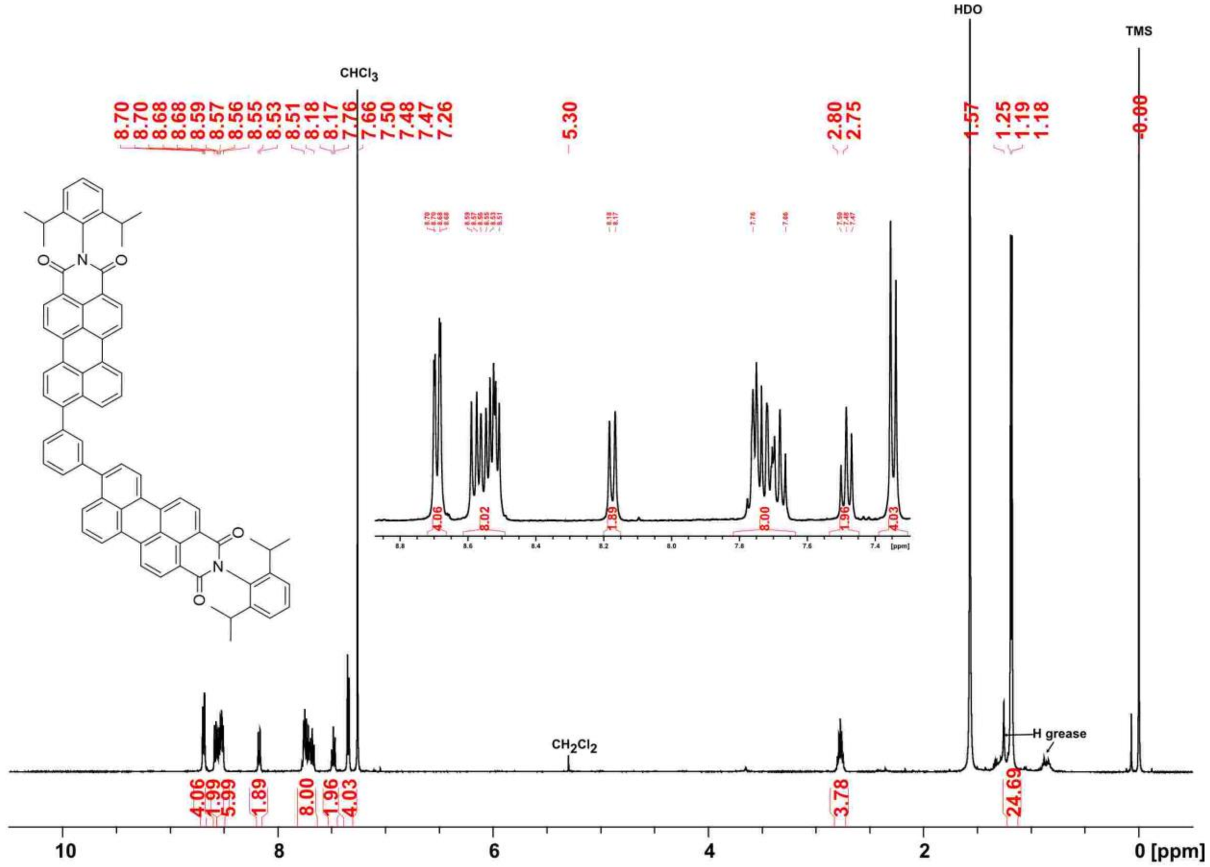

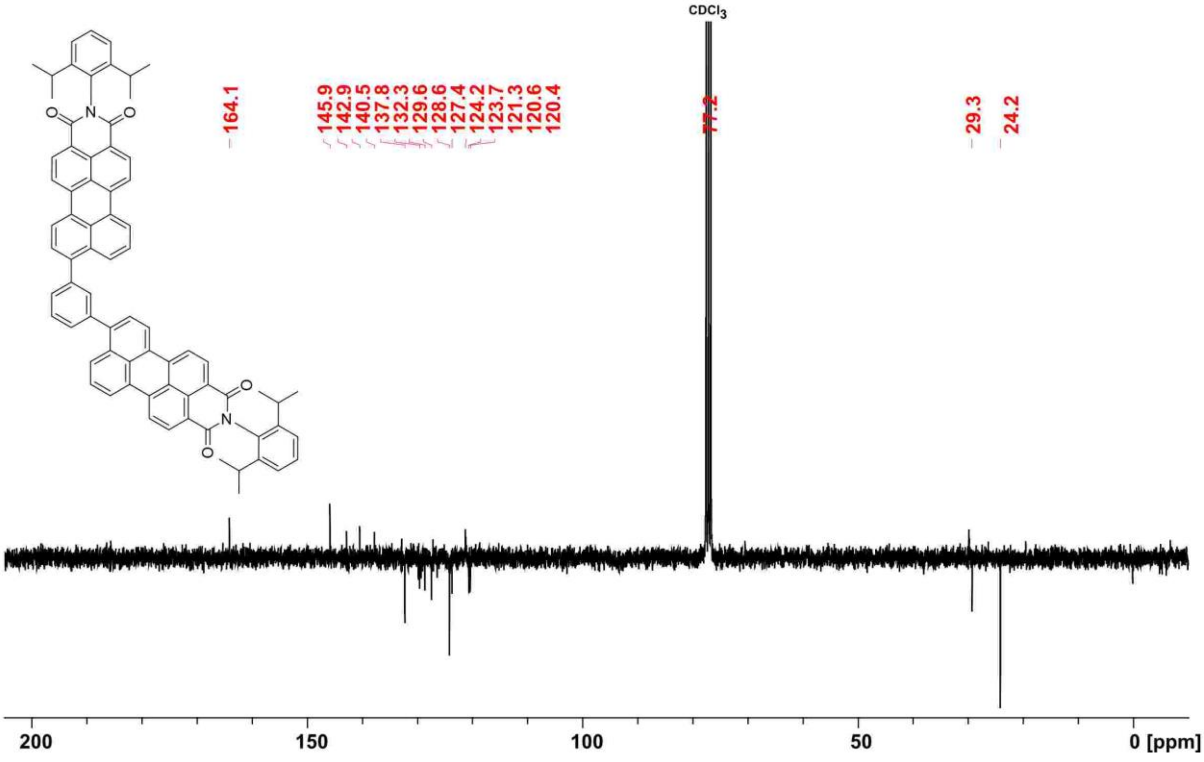

Figure S39: ^{1}H (500 MHz, CDCl$_3$) and ^{13}C-APT (75 MHz, CDCl$_3$) spectra of compound **7b**.

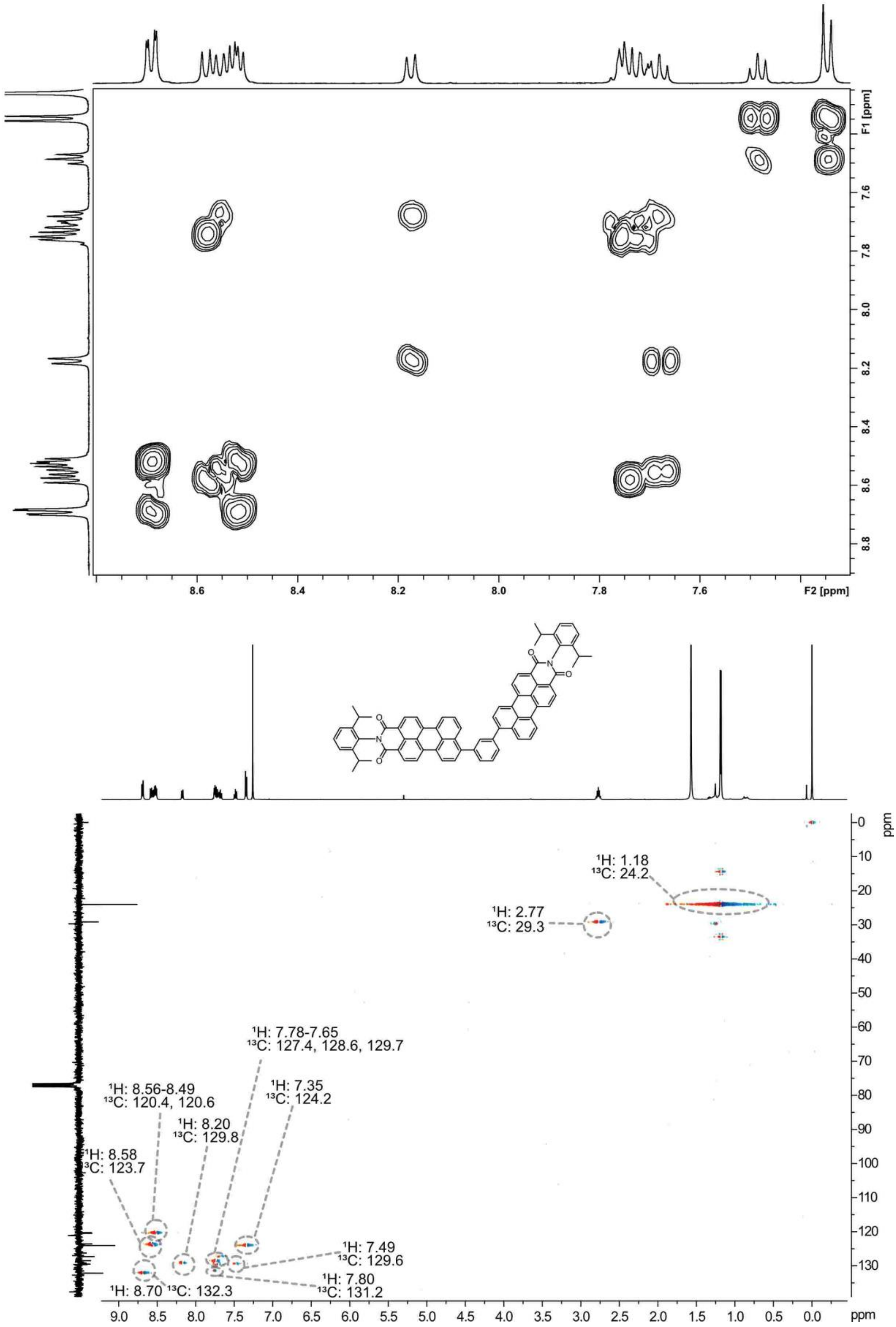

Figure S40: COSY (500 MHz, $CDCl_3$) and HSQC (500 MHz, $CDCl_3$) spectra of compound **7b**.

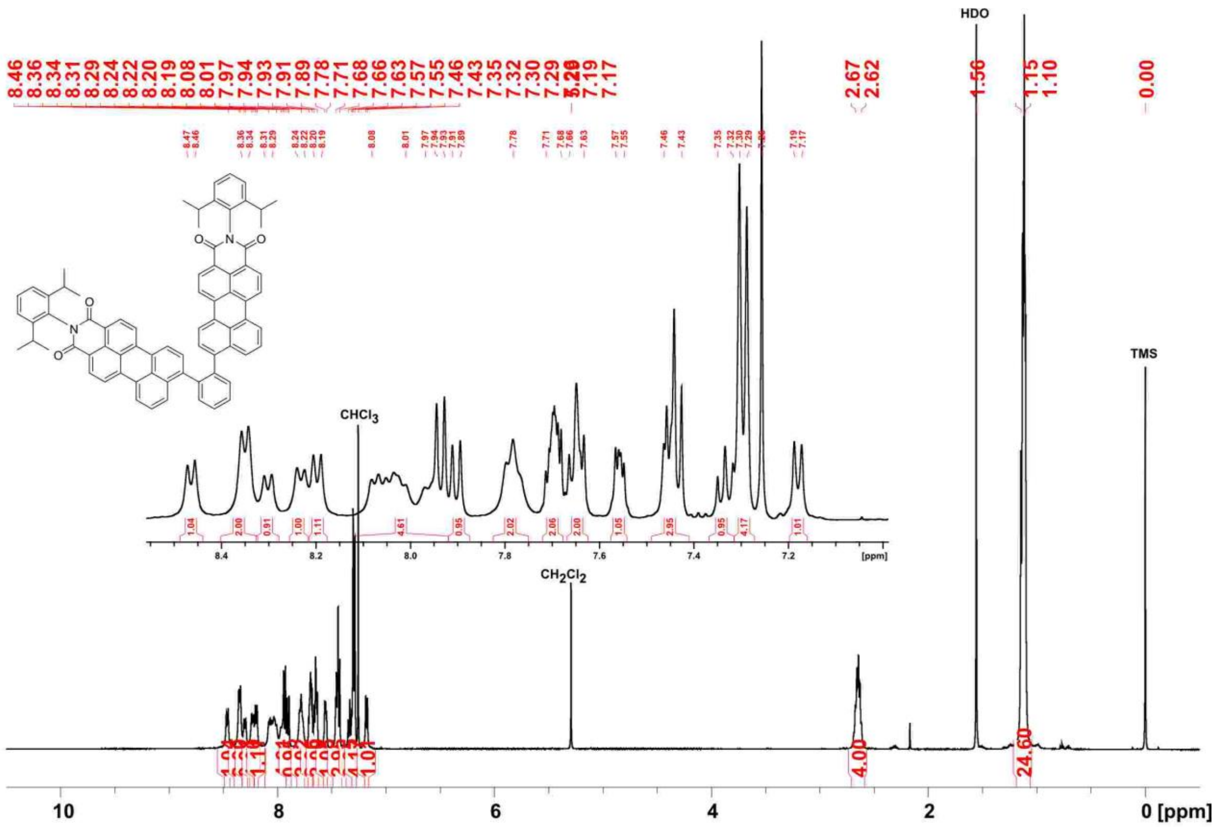

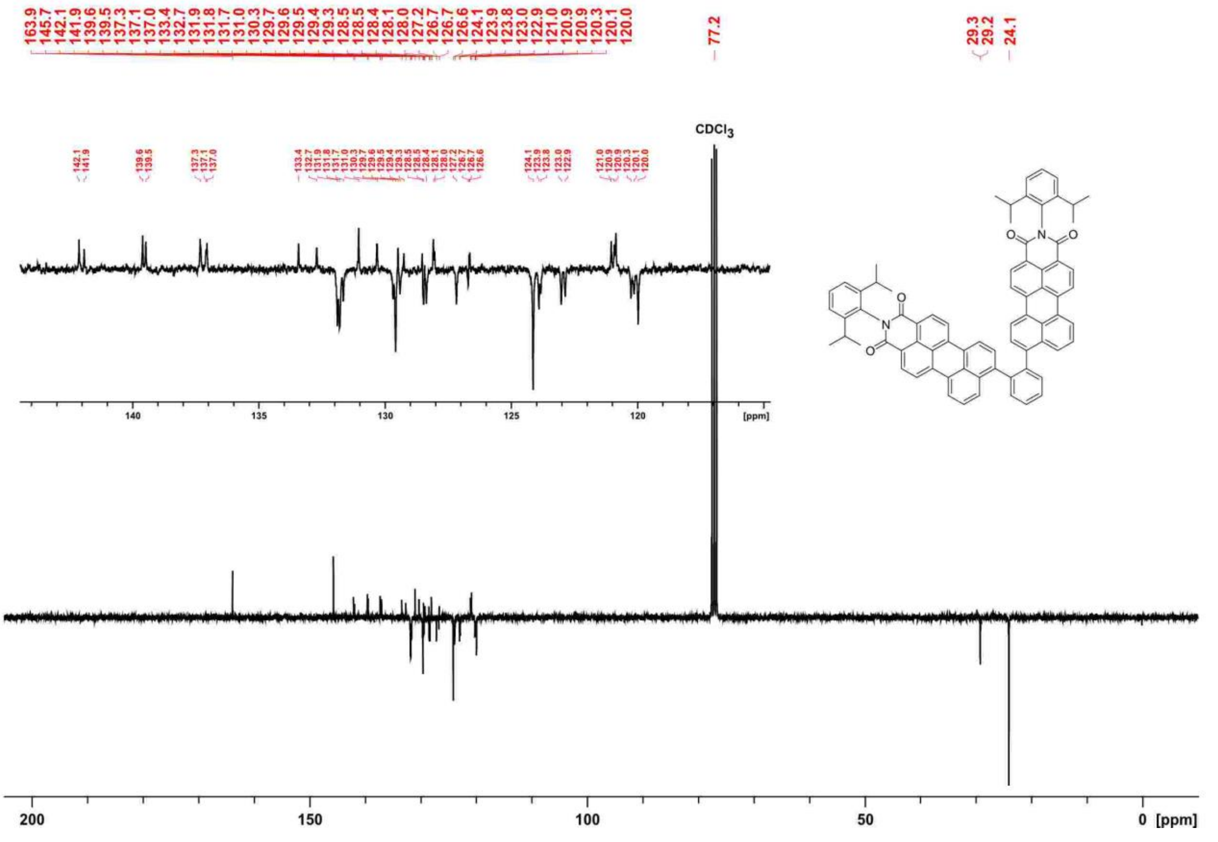

Figure S41: ^{1}H (500 MHz, $CDCl_3$) and ^{13}C-APT (75 MHz, $CDCl_3$) spectra of compound **7c**.

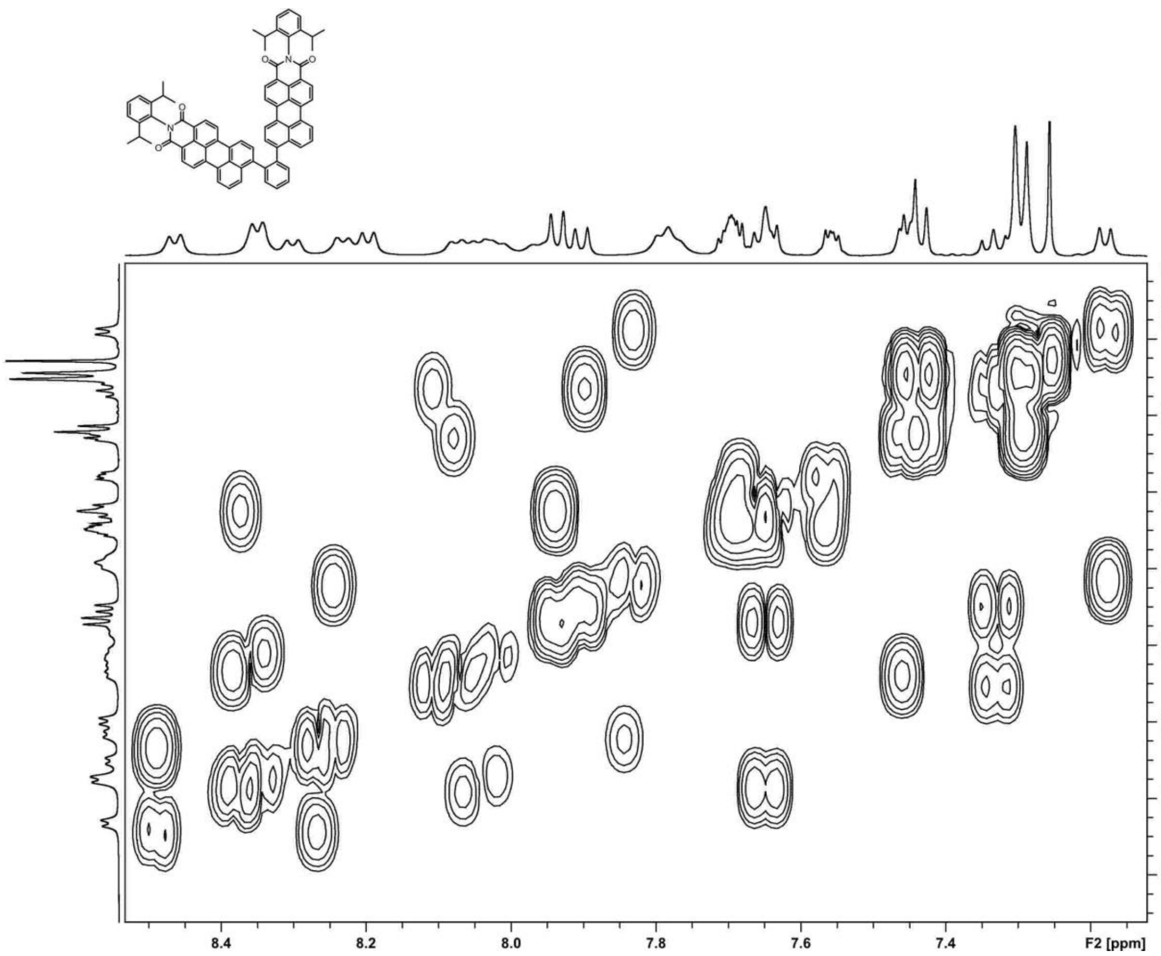

Figure S42: COSY (500 MHz, $CDCl_3$) spectrum of compound **7c**.

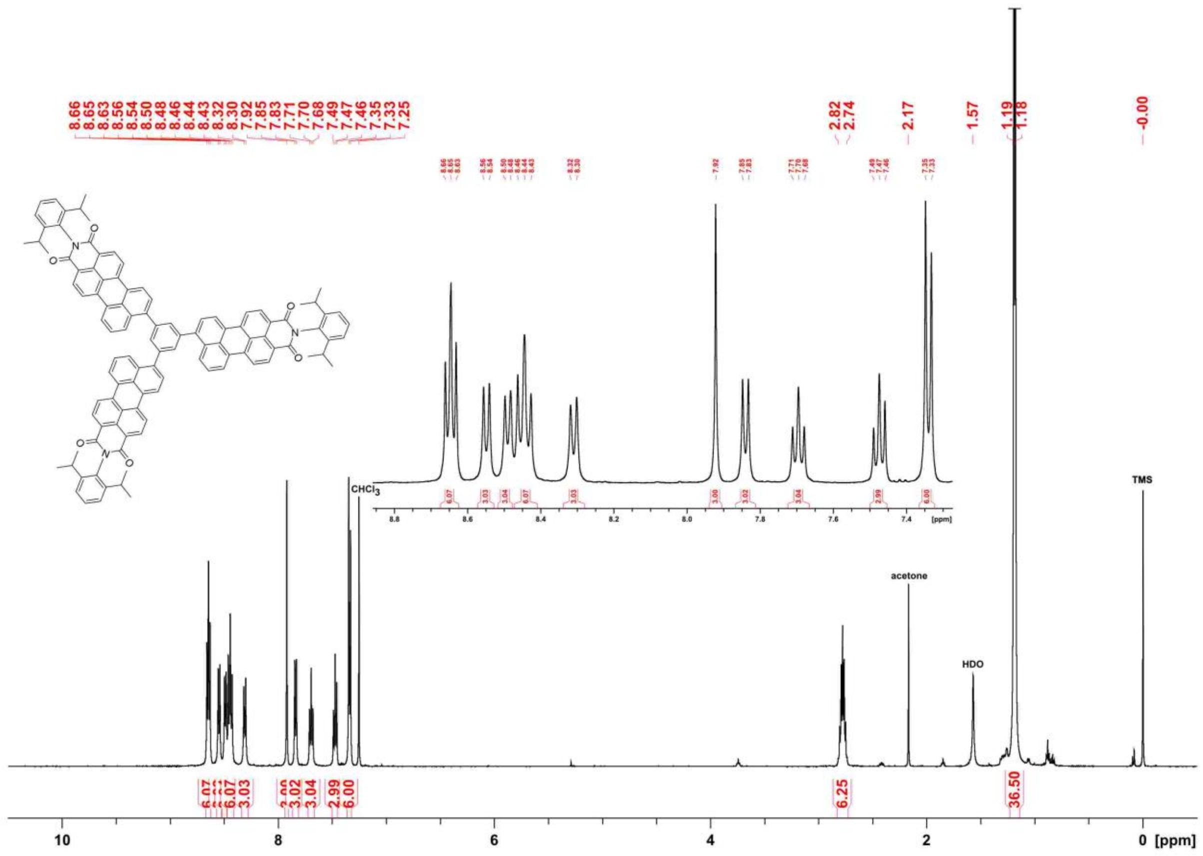

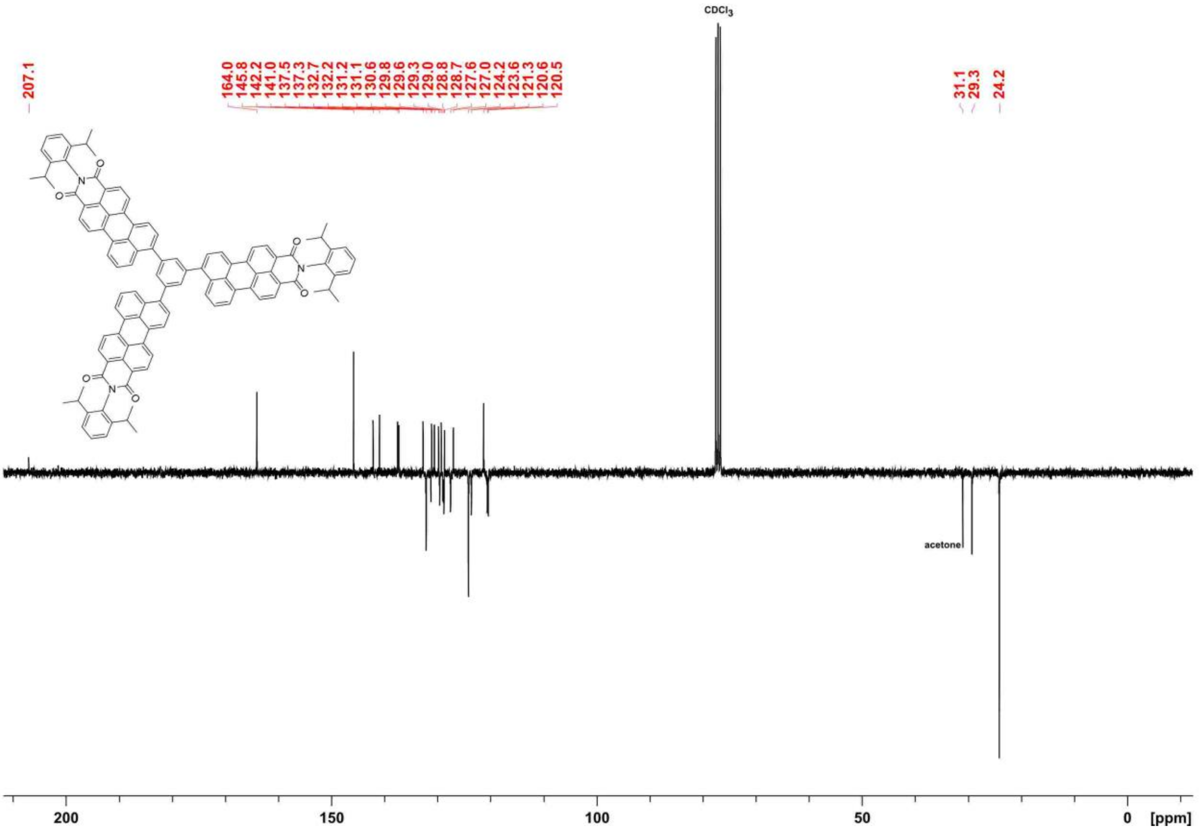

Figure S43: ^{1}H (500 MHz, $CDCl_3$) and ^{13}C-APT (75 MHz, $CDCl_3$) spectra of compound **7d**.

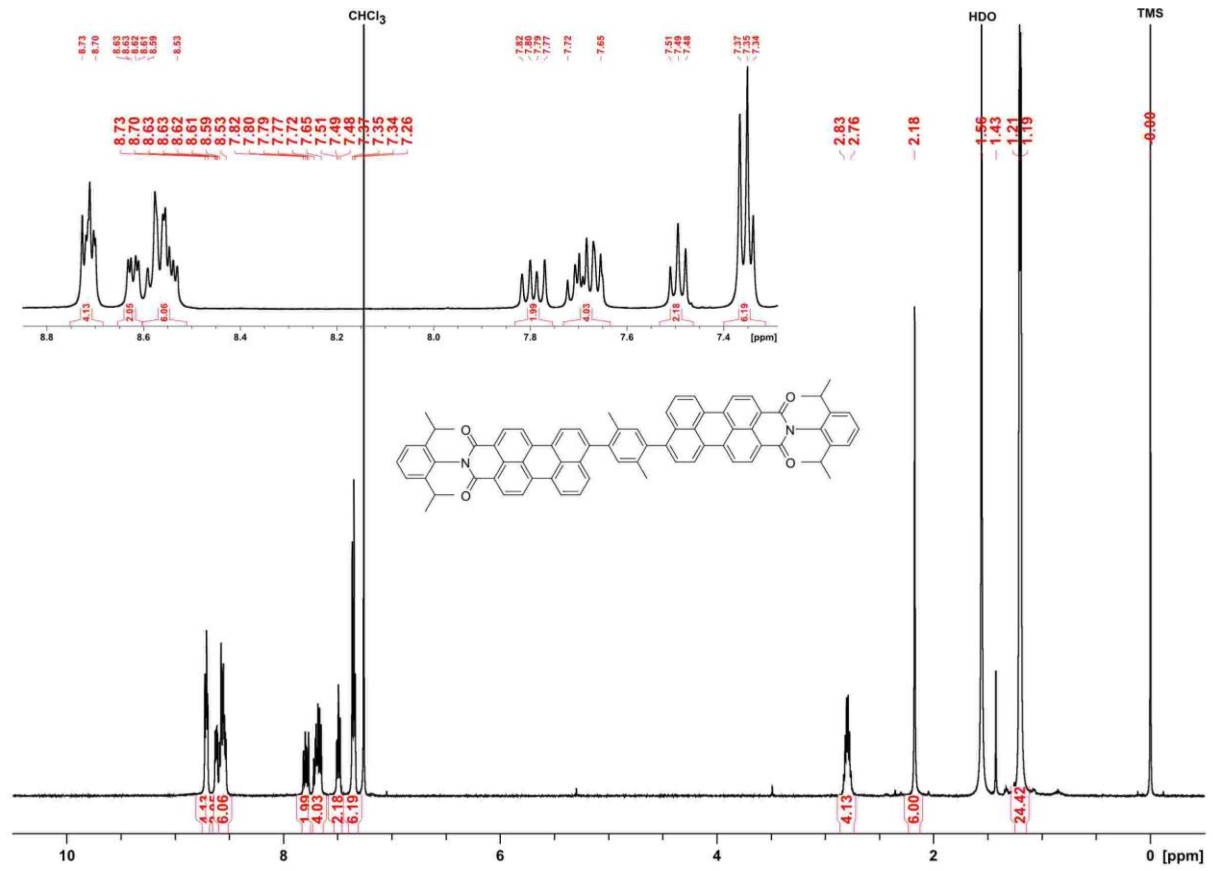

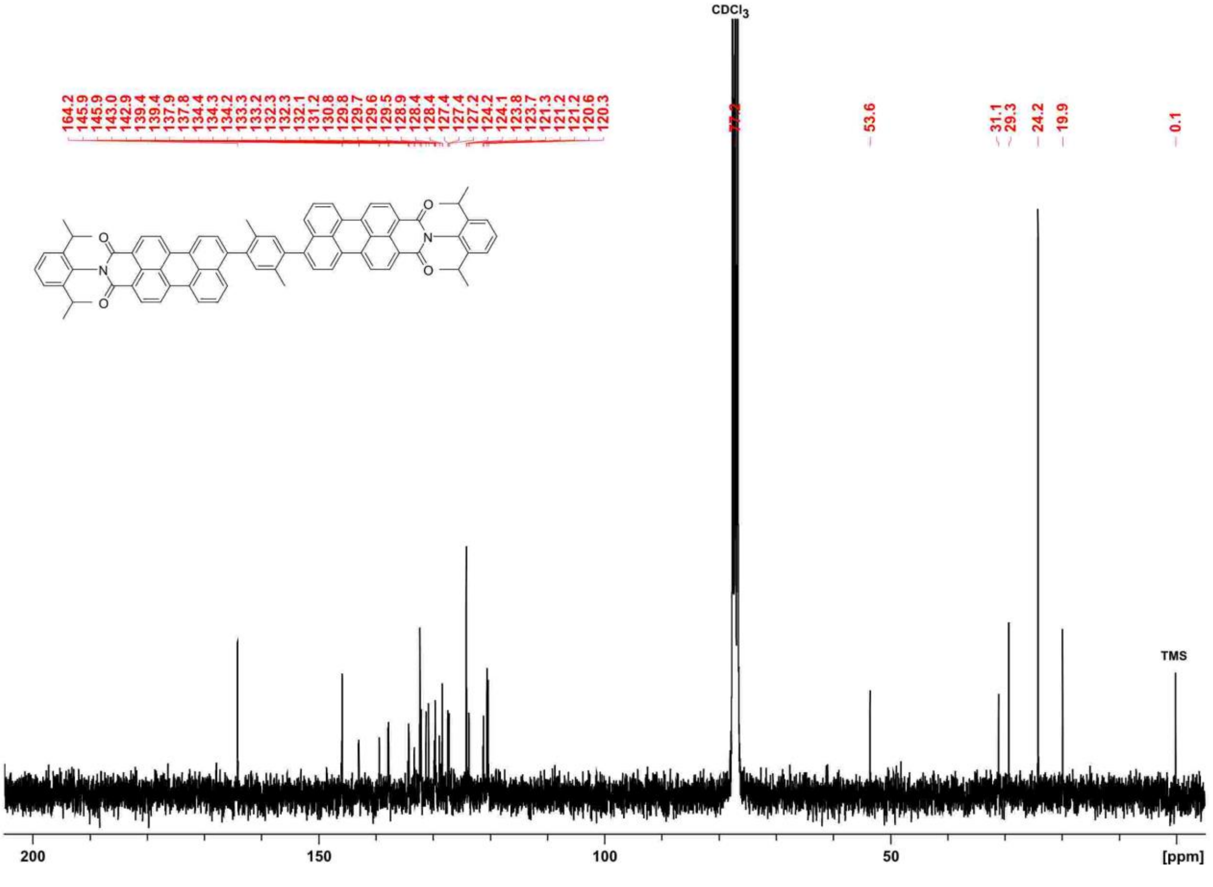

Figure S44: ^{1}H (500 MHz, $CDCl_3$) and ^{13}C (75 MHz, $CDCl_3$) spectra of compound **7e**.

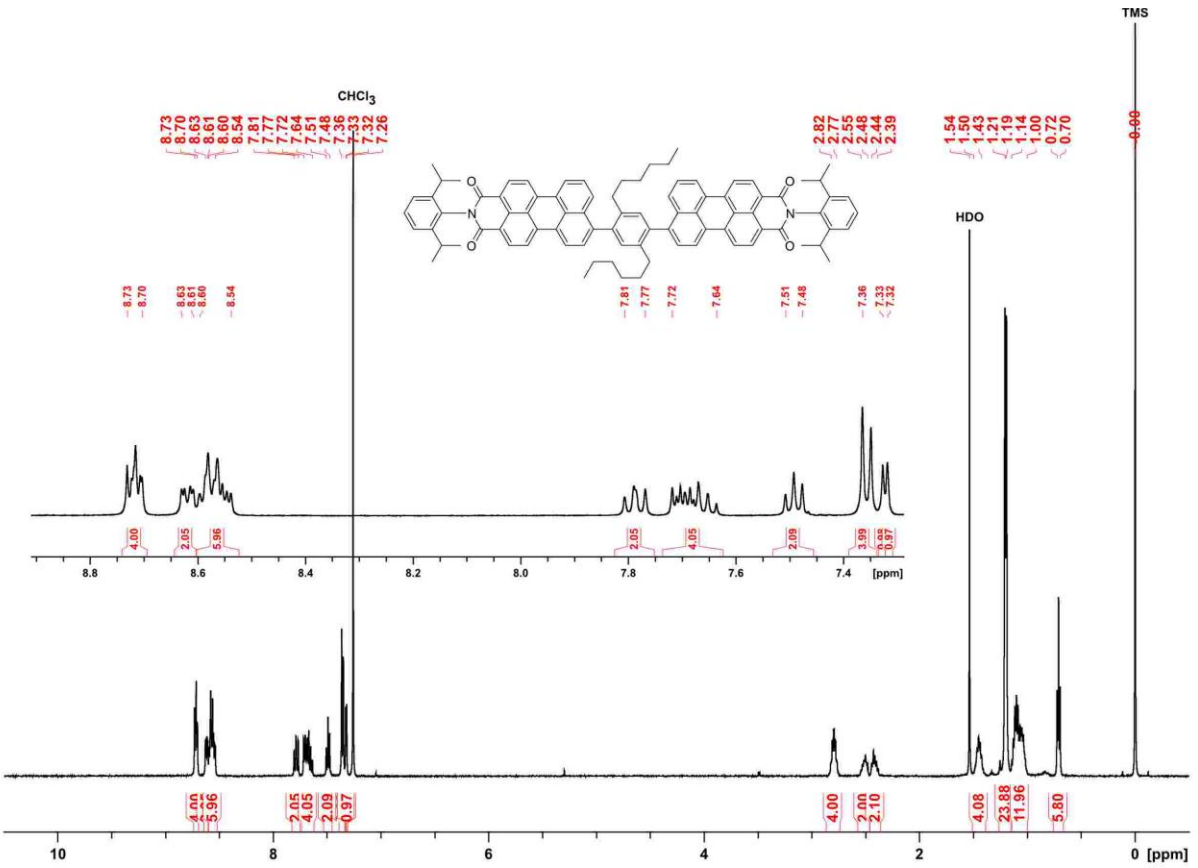

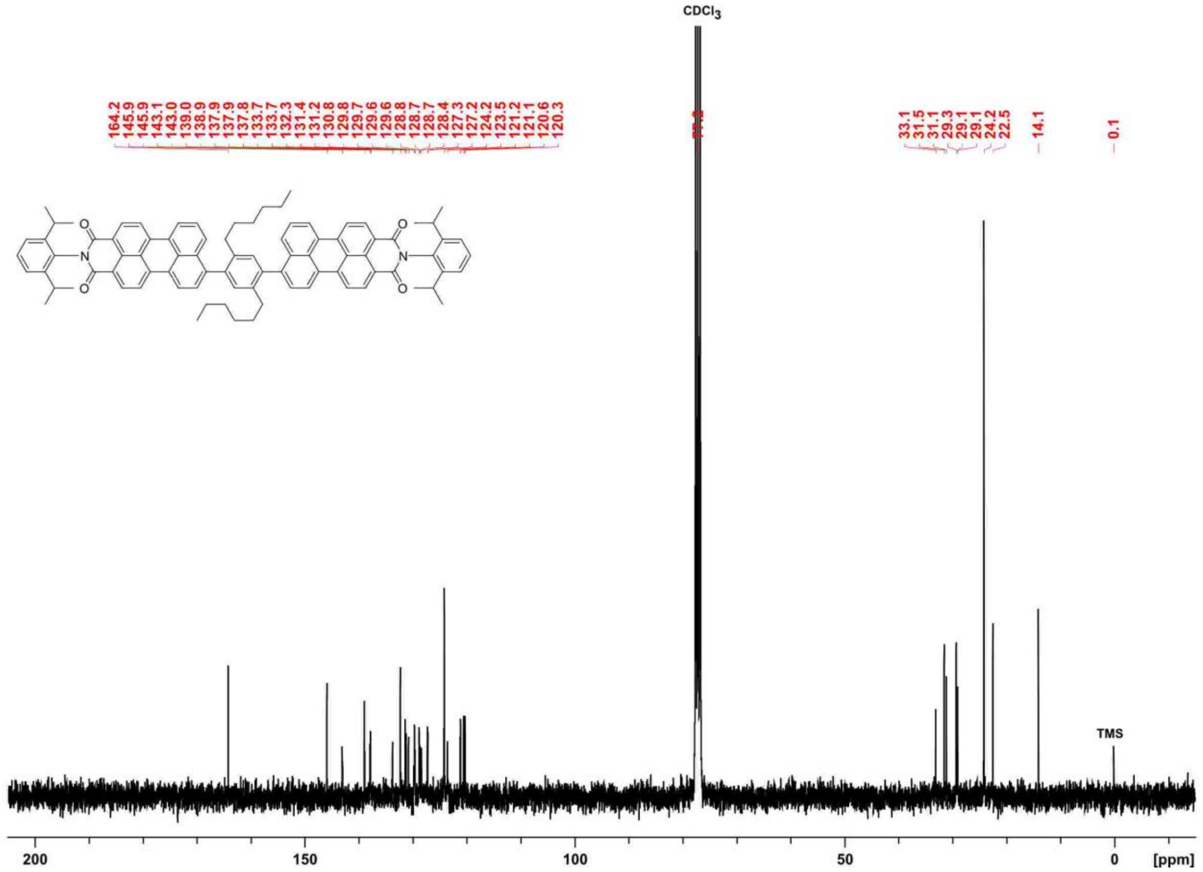

Figure S45: ^{1}H (500 MHz, $CDCl_3$) and ^{13}C (75 MHz, $CDCl_3$) spectra of compound **7f**.

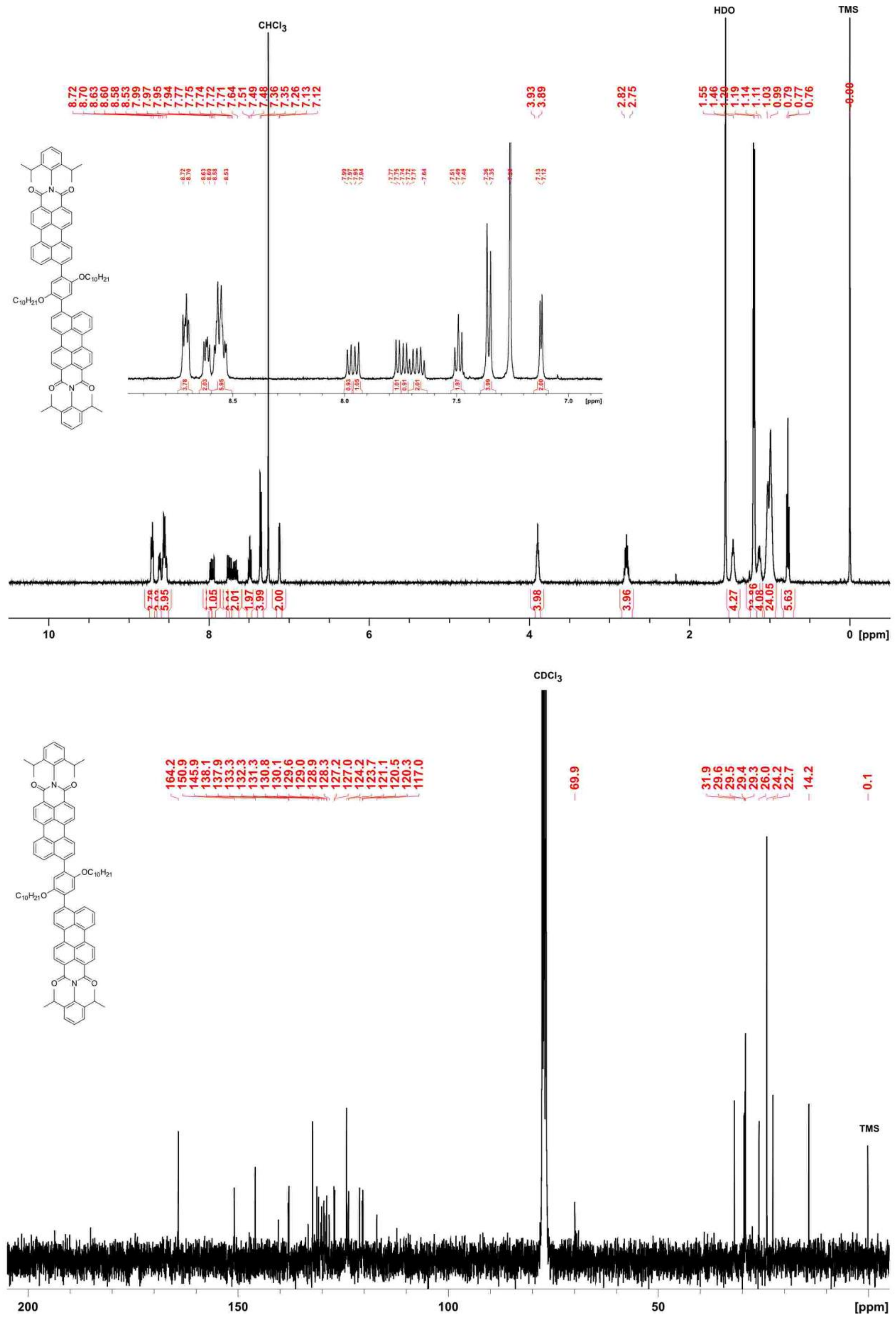

Figure S46: ^1H (500 MHz, $CDCl_3$) and ^{13}C (75 MHz, $CDCl_3$) spectra of compound **7g**.

S8. Solar cell fabrication and characterization

Organic solar cells were assembled on commercially available indium tin oxide (ITO) coated glass substrates (15 x 15 mm, 15 Ω/sq, Luminescence Technology Corp.) with the inverted device structure ITO/ZnO/PBDB-T:P-linker-P/MoO_3/Ag. Prior to the fabrication, the substrates were cleaned with deionized water, acetone and sonication in isopropanol (60 min, 40 °C). The substrates were treated with oxygen plasma (3 min, FEMTO, Diener Electronics) before the ZnO (30 nm) was deposited via spin coating (4000 rpm, 2000 rpm/s, 30 s) and subsequent thermal annealing at 150 °C (15 min). The zinc oxide precursor solution was prepared by dissolving zinc acetate dihydrate (0.5 g, 2.3 mmol) in 2-methoxyethanol (5 mL) and ethanolamine (150 µL, 2.5 mmol). For the active layer, the respective acceptor was dissolved in chlorobenzene or $CHCl_3$ in a concentration of 10 mg mL^{-1}. It was then blended with PBDB-T in a concentration of 10 mg mL^{-1}. The spin coating speed for depositing the active layer was varied from 1000 rpm to 5000 rpm at 500 rpm/s to 5000 rpm/s and 60 s. MoO_3 (10 nm) and Ag (100 nm) were deposited on the substrates via thermal evaporation through a shadow mask, giving 6 cells on each substrate with an active area of 0.09 cm^2.

Current-voltage plots were measured using a Keithley 2400 SourceMeter and a Dedolight DEB400D lamp as light source with an intensity of 100 mW cm^{-2}. A shadow mask was used for the measurements defining the active area to be 0.07 cm^2. External quantum efficiency measurements were done with a MuLTImode 4-AT monochromator (Amko) equipped with a xenon lamp (LPS 210-U, Amko) and a Keithley 2400 SourceMeter. The measurement was performed in nitrogen atmosphere. The calibration was done with a silicon photodiode (818-UV/DB, spectrally calibrated, Newport Corporation) and the spectra were monitored in the range of 380-900 nm.

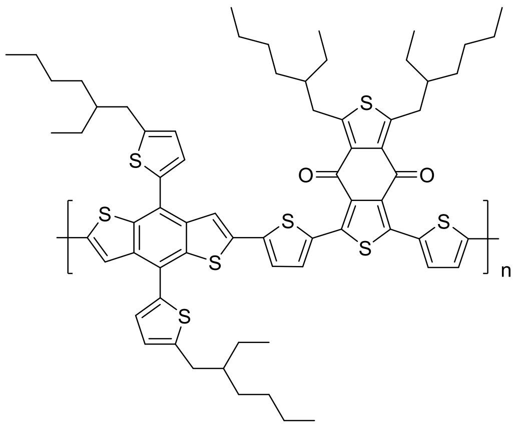

Figure S47: Chemical structure of the donor polymer PBDB-T (CAS: 1415929-80-4).

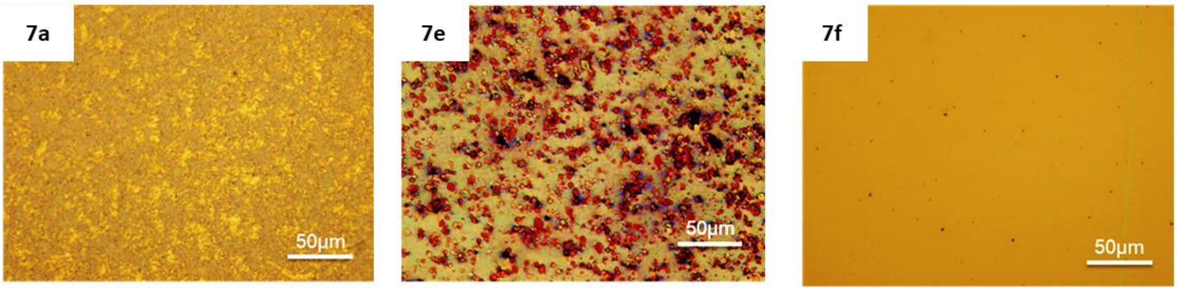

Figure S48: Light microscopy images of the compounds **7a**, **7e** and **7f** in blend with PBDB-T.

Table S4. Photovoltaic parameters and layer thicknesses of solar cells based on **6**:PBDB-T and **7d**:PBDB-T blends; average values and best values in brackets.

Compound	V_{OC} [V]	I_{SC} [mA cm^{-2}]	FF [%]	PCE [%]	Annealing	Layer Thickness [nm]
**6** (P-P)	0.84 ± 0.01 (0.83)	3.42 ± 0.54 (3.84)	39 ± 0.84 (39)	1.10 ± 0.18 (1.25)	160 °C, 10 min	155
**7d** (P_3-Ph)	0.91 ± 0.01 (0.93)	2.19 ± 0.11 (2.28)	36 ± 0.01 (37)	0.72 ± 0.04 (0.79)	--	65

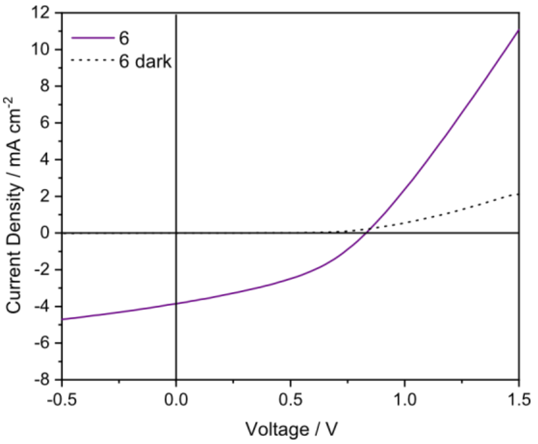

Figure S49. *J-V* characteristics of solar cells based on compound **6**.

Device Optimization

The solar cell devices were optimized concerning the annealing temperature of the active layer. The concentration was kept at 10 mg/mL of the donor PBDB-T and the D:A weight ratio was always 1:1. Optimization data are listed below for the **7c/7f/7g**:PBDB-T blend.

Table S5. Photovoltaic parameters and layer thicknesses of solar cells based on a **7c**:PBDB-T blend; average values, best values in brackets.

V_{OC} [V]	J_{SC} [mA cm^{-2}]	FF [%]	PCE [%]	Annealing	Thickness [nm]
1.16 ± 0.01 (1.16)	0.84 ± 0.06 (0.87)	28 ± 2 (30)	0.27 ± 0.02 (0.30)	-	122 ± 5
1.12 ± 0.01 (1.14)	1.55 ± 0.24 (1.91)	37 ± 1 (35)	**0.64 ± 0.08 (0.76)**	160 °C, 10 min	128 ± 5

The active layer thickness of the solar cell summarized in the Table S5 are slightly lower than the ones included in the Table 4 of the manuscript. However, since thermal annealing was profitable for the efficiencies of the cells shown in the Table S5, annealing was done also for the cells with a layer thickness of 142 nm.

Table S6. Photovoltaic parameters and layer thicknesses of solar cells based on a **7d**:PBDB-T blend; average values, best values in brackets.

V_{OC} [V]	J_{SC} [mA cm^{-2}]	FF [%]	PCE [%]	Annealing	Thickness [nm]
0.80 ± 0.05 (0.85)	1.38 ± 0.11 (1.52)	48 ± 2.26 (45)	**0.51 ± 0.05 (0.58)**	-	144 ± 5
0.69 ± 0.19 (0.83)	1.01 ± 0.15 (1.16)	38 ± 5.95 (43)	0.28 ± 0.13 (0.41)	160 °C, 10 min	129 ± 5

Table S7. Photovoltaic parameters and layer thicknesses of solar cells based on a **7f**:PBDB-T blend; average values; best values in brackets.

V_{OC} [V]	J_{SC} [mA cm^{-2}]	FF [%]	PCE [%]	Annealing	Thickness [nm]
1.18 ± 0.01 (1.18)	1.83 ± 0.11 (1.84)	28 ± 2.32 (29)	0.60 ± 0.03 (0.62)	-	140 ± 5
1.11 ± 0.01 (1.12)	3.90 ± 0.16 (3.97)	46 ± 0.56 (46)	**1.97 ± 0.04 (2.02)**	160 °C, 10 min	

Table S8. Photovoltaic parameters and layer thicknesses of solar cells based on a **7g**:PBDB-T blend, average values; best values in brackets.

V_{OC} [V]	J_{SC} [mA cm^{-2}]	FF [%]	PCE [%]	Annealing	Thickness [nm]
1.00 ± 0.02 (1.02)	4.40 ± 0.26 (4.68)	38 ± 2.38 (35)	1.65 ± 0.03 (1.68)	-	136 ± 5
0.99 ± 0.02 (1.00)	6.79 ± 0.47 (7.46)	43 ± 0.55 (43)	**2.86 ± 0.23 (3.17)**	160 °C, 10 min	

References

[1] D. Baran, A. Balan, S. Celebi, B. Meana Esteban, H. Neugebauer, N. S. Sariciftci, L. Toppare, *Chem. Mater.* **2010**, *22*, 2978–2987.

[2] T. Nishinaga in *Organic redox systems*. *Synthesis, properties, and applications* (Eds.: T. Nishinaga), John Wiley & Sons, Ltd, Hoboken, New Jersey, **2016,** p. 5.

[3] M. Burian, C. Meisenbichler, D. Naumenko, H. Amenitsch, *ArXiv* **2020**.

[4] APEX2 and SAINT, Bruker AXS Inc. Madison, Wisconsin, **2012**.

[5] a) G. M. Sheldrick, *SADABS;* Univ. Göttingen, Ger, **1996**; b) R. H. Blessing, *Acta Crystallogr. A* **1995**, *51*, 33–38;

[6] a) G. M. Sheldrick, *Acta Crystallogr. C* **2015**, *71*, 3–8; b) G. M. Sheldrick, *Acta Crystallogr. A* **2015**, *71*, 3–8;

[7] C. B. Hübschle, G. M. Sheldrick, B. Dittrich, *J. Appl. Cryst.* **2011**, *44*, 1281–1284.

[8] a) A. L. Spek, *J. Appl. Crystallogr.* **2003**, *36*, 7–13; b) A. L. Spek, *Acta Crystallogr. D* **2009**, *65*, 148–155;

[9] F. H. Allen, O. Johnson, G. P. Shields, B. R. Smith, M. Towler, *J. Appl. Crystallogr.* **2004**, *37*, 335–338.

[10] O. V. Dolomanov, L. J. Bourhis, R. J. Gildea, J. A. K. Howard, H. Puschmann, *J. Appl. Crystallogr.* **2009**, *42*, 339–341.

[11] C. F. Macrae, I. Sovago, S. J. Cottrell, P. T. A. Galek, P. McCabe, E. Pidcock, M. Platings, G. P. Shields, J. S. Stevens, M. Towler, P. A. Wood, *J. Appl. Crystallogr.* **2020**, *53*, 226–235.

[12] Y. Geerts, H. Quante, H. Platz, R. Mahrt, M. Hopmeier, A. Böhm, K. Müllen, *J. Mater. Chem.* **1998**, *8*, 2357–2369.
